# Circularly Polarized Luminescent and Melt‐Processable Copper(I)‐Organic Glasses Based on 2,2′‐Bis(diphenylphosphino)‐1,1′‐binaphthyl

**DOI:** 10.1002/anie.9766332

**Published:** 2026-05-07

**Authors:** Zeyu Fan, Indranil Sen, Roman Pallach, Guo‐Qiang Li, Andreas Steffen, Sebastian Henke

**Affiliations:** ^1^ Anorganische Chemie, Fakultät für Chemie und Chemische Biologie Technische Universität Dortmund Dortmund Germany

**Keywords:** metal–organic glass, glass formation, vitrification, thermally activated delayed fluorescence, circular polarized luminescence

## Abstract

The melting and vitrification behavior of metal–organic materials endows them with high processability, enabling the formation of bulk solids with potential in optical applications. Nevertheless, obtaining metal–organic glasses with chiroptical properties, such as circularly polarized luminescence (CPL), remains challenging due to the limited thermal and mechanical stability of current chiral metal‐organic materials. Here, we report a pair of homochiral copper(I) cyanide complexes based on 2,2′‐bis(diphenylphosphino)‐1,1′‐binaphthyl (BINAP) that can be readily vitrified by either melt‐quenching or desolvation. Structural analysis reveals that vitrification disrupts only the intermolecular stacking of the complexes, while preserving the local coordination environment. The resulting melt‐quenched glasses are CPL‐active and exhibit yellow–orange triplet excited state decay via thermally activated delayed fluorescence (TADF) at room temperature, which is environment‐dependent due to an additional ^3^BINAP↔^3^(Cu→BINAP) CT (CT = charge transfer) equilibrium. Centimeter‐sized monoliths and thin films can be fabricated by molding and thermal annealing, highlighting their excellent processability. Moreover, the glasses can be recrystallized by solvent vapor treatment, which triggers distinct changes in luminescent properties. These results establish BINAP‐based copper(I) cyanide complexes as a versatile platform for the design of CPL‐active metal–organic glasses and point to their promise in optoelectronic applications.

## Graphical abstract

Homochiral Cu(I) cyanide complexes based on 2,2’‐bis(diphenylphosphino)‐1,1’‐binaphthyl (BINAP) form melt‐quenched and desolvation‐derived metal–organic glasses that exhibit circularly polarized thermally activated delayed fluorescence (TADF) at room temperature, enabling processable chiroptical materials.

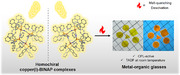

## 1 Introduction

Chirality is a fundamental feature of nature. Chiral substances, including proteins, DNA and sugars, are indispensable to the evolution and functioning of living systems on Earth [1, 2, 3]. Understanding the assembly and behavior of chiral substances not only provides insights into the origin of life but also drives advances in functional materials, particularly optoelectronics. Considerable effort has therefore been devoted to circularly polarized luminescence (CPL), a chiroptical phenomenon that arises from the photoemission of chiral luminophores [4]. CPL‐active materials have attracted broad interest due to their potential applications in optoelectronic displays [5, 6, 7, 8, 9], information storage [10, 11, 12], and chiral sensing and probing [13, 14, 15, 16, 17]. Metal–organic materials, including metal complexes [18, 19, 20, 21, 22, 23, 24], cages [25, 26, 27, 28, 29], coordination polymers (CPs) [30, 31, 32, 33], and metal–organic frameworks (MOFs) [34, 35, 36, 37, 38], which are constructed from metal ions or clusters and organic ligands, have emerged as particularly promising CPL candidates. Their modular design allows for a versatile combination of inorganic nodes and organic moieties, endowing them with highly tunable structures and photophysical properties [39, 40, 41, 42]. However, most reported systems are in the form of small single crystals and polycrystalline powders. The crystalline nature limits processability, preventing the formation of bulk materials with defined shapes. In addition, light scattering at grain boundaries and particle surfaces/interfaces reduces optical transparency, leading to significant energy loss during optical transport [43].

Glasses are amorphous inorganic, (metal–)organic or metallic solids with broad technological applications. Certain crystalline CPs/MOFs and metal complexes have been shown to form glassy states by melt‐quenching, mechanical milling, or solvent removal [44, 45]. The dynamic structure, porosity, and processability of these glasses open opportunities for gas separation, ion transport, and catalysis [46, 47, 48, 49]. Importantly, vitrification imparts processability, enabling the fabrication of bulk materials suitable for device integration [50]. The optical transparency and isotropic character of glasses also suppress light scattering, making them attractive for photonic applications [51]. Despite these advantages, the development of CPL‐active metal–organic glasses remains in its infancy, and no such systems have yet been realized via melt‐quenching or mechanical milling [52, 53]. Conventional organic ligands used to construct chiral metal–organic materials often rely on central chirality, as in histidine‐ or camphoric acid‐derived systems, where chirality originates from different substituents around a stereogenic center (Figure S1A). However, such substituents (e.g., amino or carboxylate groups) can compromise the thermal and mechanical stability of the materials, thereby hindering melting and glass formation [54]. Furthermore, vitrification of framework materials typically reduces porosity, limiting the use of host–guest chemistry to generate CPL [55].

BINAP (2,2′‐bis(diphenylphosphino)‐1,1′‐binaphthyl) represents a prototypical CPL‐active molecule featuring axial chirality [56]. Its chirality originates from restricted rotation between two naphthalene planes, enforced by the steric hindrance of the bulky ─PPh_2_ groups (Figure S1B). The electron‐rich phosphorus(III) atoms provide coordination sites for soft metal centers [57, 58, 59, 60, 61]. The non‐centrosymmetry and nonplanar geometry of BINAP favors the crystallization of low‐symmetry structures, which may be more susceptible to melting and vitrification [62, 63, 64]. Moreover, BINAP exhibits an exceptionally high racemization barrier (∆*G* ^‡^ > 200 kJ mol^−1^), ensuring the retention of its chiroptical properties upon high‐temperature treatment [65].

Guided by these considerations, we report two homochiral metal–organic complexes based on *R‐* or *S*‐BINAP and CuCN. Crystals of both complexes can be vitrified by either melt‐quenching or desolvation, yielding transparent glasses that exhibit yellow–orange thermally activated delayed fluorescence (TADF) from ^1/3^MLCT (metal‐to‐ligand charge transfer) states at room temperature. Experimental and calculation results demonstrate that the environment‐dependent equilibrium between ^3^LC↔^3^MLCT (LC = ligand centered) plays a key role in the photoluminescent properties. The structure of crystalline and glassy phases is comprehensively characterized using infrared and nuclear magnetic resonance (NMR) spectroscopy, as well as X‐ray diffraction, total scattering and absorption spectroscopy. Notably, vapor treatment of the glasses induces recrystallization and leads to distinct changes in their luminescence properties. To the best of our knowledge, this work represents the first demonstration of CPL‐active metal–organic glasses showing TADF obtained via melt‐quenching, establishing such complexes as a promising platform for chiroptical materials and opening new avenues for their integration in optoelectronic devices.

## 2 Results and Discussion

### 2.1 Crystal Structure and Thermal Behavior

**FIGURE 1:**
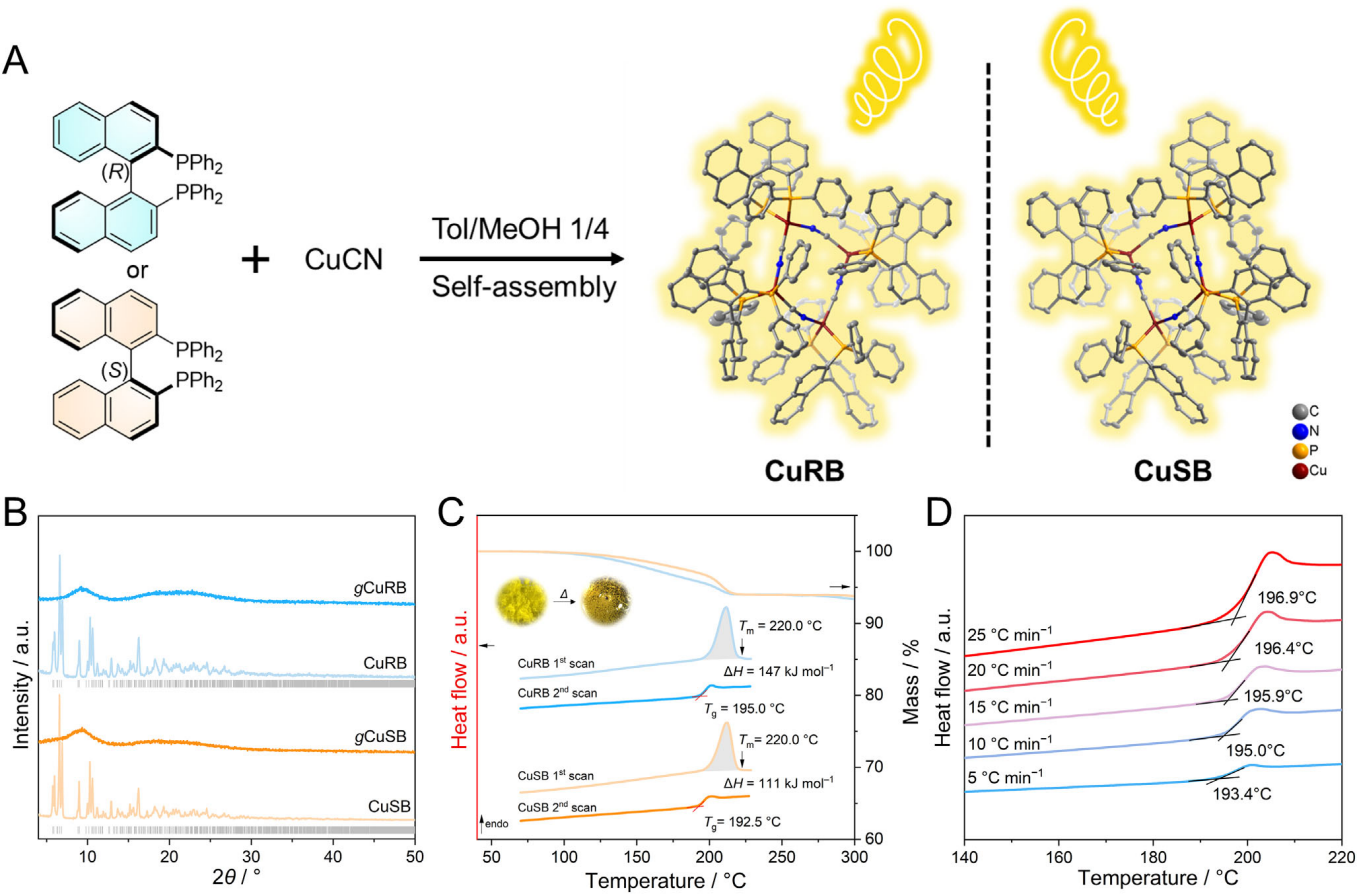
(A) Synthesis and crystal structure of the homochiral metal complexes **CuRB** and **CuSB**. H atoms are omitted for clarity. Ellipsoids are shown at the 50% probability level. (B) PXRD patterns of **CuRB**, **CuSB**, ***g*CuRB,** and ***g*CuSB**. (C) TGA (right vertical scale) and DSC (left vertical scale) profiles of **CuRB** and **CuSB**. For DSC, the samples were cycled from 30 to 230°C with a heating/cooling rate of 10°C min^−1^. The inset shows the significant morphological change of **CuRB**, indicating the melting behavior. (D) DSC upscans of ***g*CuRB** with variable heating rates from 5 to 25°C min^−1^.

The homochiral tetranuclear copper(I) complexes Cu_4_(CN)_4_(*R*‐BINAP)_4_·guest (denoted as **CuRB**) and Cu_4_(CN)_4_(*S*‐BINAP)_4_·guest (denoted as **CuSB**) were obtained as yellow single crystals by slow diffusion of methanol into a toluene solution containing *R*‐ or *S*‐BINAP and CuCN (Figure 1A, see experimental section). Single‐crystal X‐ray diffraction (SCXRD) analyses revealed **CuRB** and **CuSB** are enantiomers and crystallize in the non‐centrosymmetric triclinic space group *P*1 (Tables S1 and S2). In these crystal structures, each copper(I) atom adopts a distorted tetrahedral coordination environment, being bound to two phosphorus atoms from one BINAP ligand and two carbon/nitrogen atoms from cyanide ligands. Four Cu‐BINAP units are connected by four cyanide bridges to form a metallocyclic square with a diameter of ∼2.2 nm (Figure S2). Toluene and H_2_O molecules are located in the crystal cavities as guest species. Slow diffusion of toluene solution containing CuCN/*R*‐BINAP into another containing CuCN/*S*‐BINAP yields the *meso*‐complex Cu_4_(CN)_4_(*R*‐BINAP)_2_(*S*‐BINAP)_2_·3toluene (denoted as ***meso*CuB**), which crystallizes in the centrosymmetric tetragonal space group *I*4_1_/*a* (Figure S3; Table S3). ***meso*CuB** shares the same molecular connectivity as the chiral structures, but the *R*‐ and *S*‐BINAP ligands arrange alternately around the Cu_4_(CN)_4_ square so that opposite positions are occupied by the same enantiomer. The microcrystalline powders of the homochiral complexes were obtained as phase‐pure samples, as confirmed by Pawley refinements of their powder X‐ray diffraction (PXRD) patterns (Figure 1B and S4 and S5; Table S5).

Thermogravimetric analysis (TGA) of **CuRB** and **CuSB** shows an initial mass loss of ∼6% below 220°C, attributable to the release of two toluene molecules per Cu_4_(CN)_4_(BINAP)_4_ unit (Figures S6 and S7). No further mass loss is observed until decomposition above 300°C. Variable‐temperature PXRD demonstrates that both complexes gradually lose crystallinity upon heating and become fully amorphous by 220°C, indicating complete loss of crystallinity upon desolvation (Figures S8 and S9). In differential scanning calorimetry (DSC), pronounced endothermic peaks with offset temperatures at 220°C correspond to the desolvation process (Figure 1C). The narrower temperature interval assigned to desolvation in DSC (190 to 220°C) compared with that in TGA (∼100 to 220°C) arises from differences in sample environment (an open alumina crucible for TGA versus a sealed aluminum pan with a small pinhole in DSC), which retard solvent escape and shift the desolvation endotherm to higher temperatures in DSC. Concomitantly, optical microscopy reveals that the particles coalesce into fused morphologies showing clear evidence of flow (Figure 1C insert), suggesting that desolvation and melting occur in close succession. The enthalpy changes (Δ*H*) of the desolvation‐driven liquefaction amount to 147 and 111 kJ mol^−1^ for **CuRB** and **CuSB**, respectively, which substantially exceeds twice the sublimation enthalpy of toluene at standard conditions (∆*H*_sub_° = 43.1 kJ mol^−1^) [66]. The excess endothermic enthalpy of the liquefaction process (relative to 2 × Δ*H*_sub_° of toluene) reflects not only evaporation of confined solvent guests but also the enthalpic cost of disrupting host‐guest interactions and intermolecular packing (*π–π* and CH–*π* contacts), together with the latent heat associated with the solid‐liquid transition.

Temperature‐modulated DSC (mDSC) measurements provide further insights into the coupled desolvation‐melting process (Figures S10 and S11) [67]. In mDSC, a small sinusoidal temperature modulation (±1°C, 120 s period) is superimposed on the linear heating ramp (see Supplementary Information for details). This approach separates reversible processes (those that instantaneously follow the temperature modulation) from kinetic or non‐reversible events, which lag behind. In the present case, the non‐reversing heat flow exhibits a sharp endothermic peak corresponding to the combined desolvation and melting enthalpies of the complexes, whereas the reversing heat flow shows a concurrent step change reflecting the increase in heat capacity (Δ*C*_p_) accompanying the solid‐liquid transition. Notably, the peak temperature of the non‐reversing signal is only slightly lower than the midpoint of the Δ*C*_p_ step, demonstrating that desolvation and melting are nearly synchronous processes. These observations confirm that guest release directly triggers structural collapse into a viscous, supercooled liquid rather than producing an intermediate solid phase.

Cooling the liquid obtained at 230°C to room temperature yields transparent amorphous phases denoted ***g*CuRB** and ***g*CuSB** (***g*** = glass), exhibiting distinct glass transitions (*T*_g_) at 195.0 and 192.5°C, respectively, in the second DSC upscans (Figure 1C). PXRD confirmed the complete loss of long‐range order in these vitrified materials (Figure 1B). The Δ*C*_p_ at *T*_g_ are 0.31 and 0.27 J g^−1^°C^−1^ for ***g*CuRB** and ***g*CuSB**, respectively (Figure S12). These values are considerably higher than those typically reported for MOF glasses (∼0.1 J g^−1^°C^−1^), indicating large configurational freedom and comparatively weak topological constraints in the supercooled liquids of the metal complexes [68]. The stability of the glass transition is verified by five consecutive DSC heating‐cooling cycles (Figures S13 and S14), which show no change in *T*_g_.

Additional DSC experiments with heating rates between 5 and 25°C min^−1^ were performed to determine the calorimetric fragility indices (*m*); a measure of the rate at which viscosity changes with temperature near *T*_g_ (Figures 1D and S15). Fragility indices (*m*) of 95 and 91 are derived for ***g*CuRB** and ***g*CuSB**, respectively; values that far exceed those of conventional silica glasses (*m* = 20) and ZIF glasses (*m* = 20–40) (Figures S16 and S17) [69, 70]. Such high fragility indices reflect the steep temperature dependence of viscosity around *T*_g_ and imply weak intermolecular interactions and high molecular mobility in these metal–organic liquids [71, 72]. Notably, no exothermic crystallization peak is observed between 220 and 300°C (Figure S18), indicating that the chiral complexes do not recrystallize from the liquid phase prior to decomposition. This pronounced kinetic stability likely arises from the low molecular symmetry of the complexes and the absence of solvent molecules required to fill intrinsic voids within the crystal lattice, leading to inefficient molecular packing and suppression of crystallization.

The glassy states are also accessible by solvent removal below the materials’ *T*_g_. Heating **CuRB** and **CuSB** at 190°C under dynamic vacuum for 24 h yields the amorphous solids ***ds*CuRB** and ***ds*CuSB** (Figure S19, ***ds*** = desolvated). TGA confirms the absence of further mass loss up to 300°C, while DSC reveals *T*_g_ signals at 200.6 and 200.9°C, slightly higher than those of the melt‐quenched glasses, together with pronounced enthalpy recovery (Figures S20–S23). Finally, the *meso*‐complex ***meso*CuB** also forms a glass via desolvation‐driven liquefaction followed by cooling (Figures S24–S26), with melting completed at 215°C and a *T*_g_ at 194.7°C in the second upscan.

### 2.2 Structural Analysis

Solution‐state ^1^H, ^13^C and ^31^P NMR spectroscopy was used to verify the chemical composition of the materials (Figures S27–S45). In the NMR spectra of the crystalline samples, resonances at 2.3, 7.1 and 7.2 ppm correspond to toluene guest molecules. The integrated ratio of metal complexes to toluene is 1:2, consistent with the crystal structures and TGA data. In contrast, these toluene signals are absent in the NMR spectra of the glasses, confirming complete solvent removal by the high‐temperature desolvation‐driven liquefaction process. No shifts or additional resonances indicative of decomposition are observed, demonstrating that apart from desolvation, the chemical composition of the complexes remains unchanged upon vitrification. This is also supported by elemental analysis of these complexes in both crystalline and glassy states (Table S6). Diffusion‐ordered NMR spectroscopy (DOSY) of ***ds*CuRB** and ***ds*CuSB** shows that all signals of the complexes feature a single diffusion coefficient (*D*) of ∼3.8 × 10^−10^ and ∼4.2 × 10^−10^ m^2^ s^−1^, corresponding to hydrodynamic diameters of ∼2.2 and ∼2.1 nm, respectively (Figures S46 and S47). These values agree well with the molecular dimensions obtained from the crystal structures, indicating that the metal complexes remain intact in the solution without dissociation (Figure S2).

**FIGURE 2:**
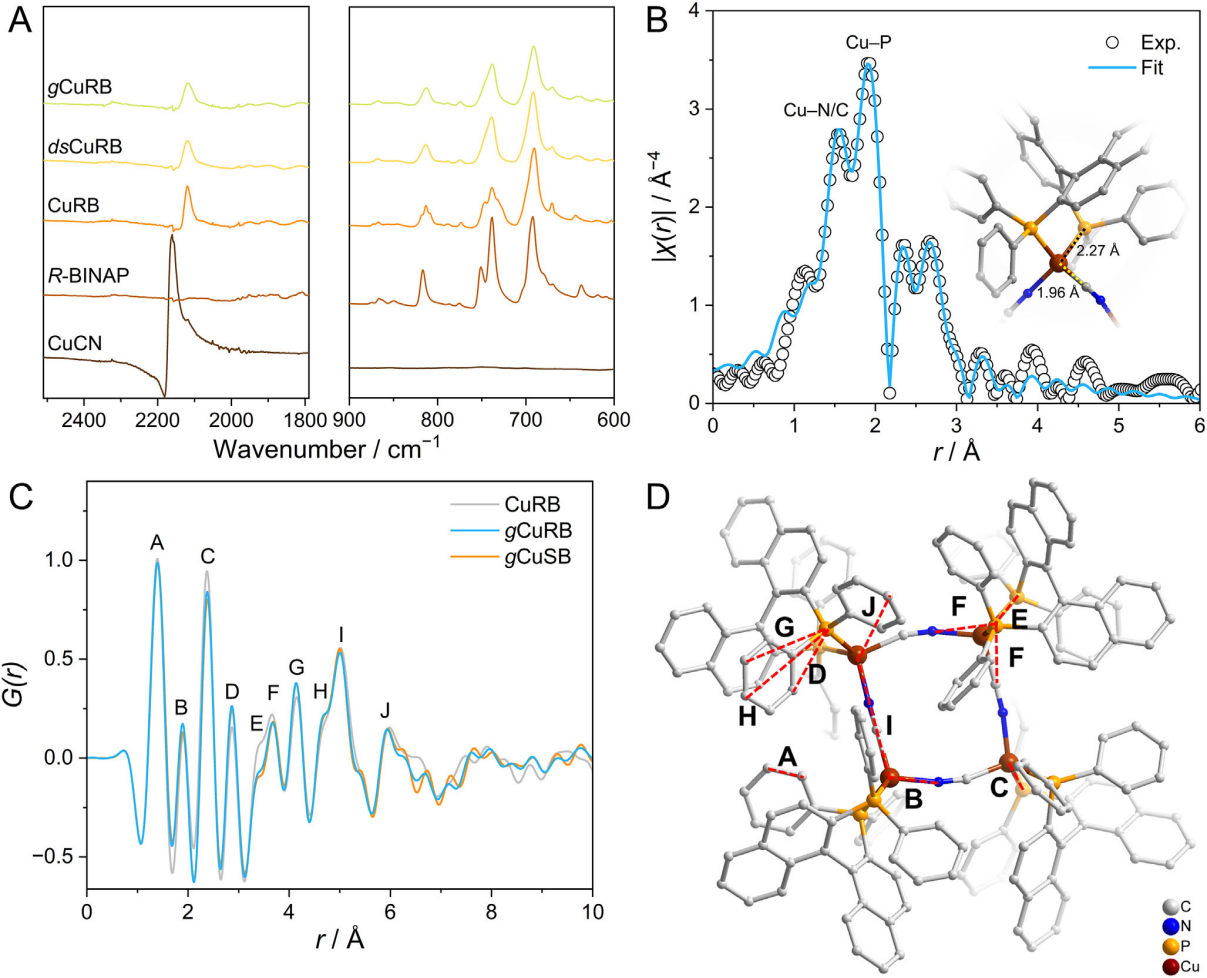
(A) Zoomed FT‐IR spectra of CuCN, *R*‐BINAP, **CuRB**, ***ds*CuRB,** and ***g*CuRB** from 2500 to 1800 cm^−1^ and 900 to 600 cm^−1^. (B) EXAFS fitting of ***g*CuRB** in the range of 1–3 Å by using the single crystal data of **CuRB**. (C) PDF profiles of **CuRB**, ***g*CuRB,** and ***g*CuSB** derived from the X‐ray total scattering data. The peaks A–J correspond to the distances shown in (D). (D) Examples of atomic pair correlations corresponding to peaks A–J in the PDF profile in (C). H atoms are omitted for clarity.

Solid‐state Fourier‐transform infrared (FT‐IR) spectroscopy further elucidates the structural features of the materials. In the spectra of **CuRB** and **CuSB**, a resonance at 2120 cm^−1^ is assigned to the cyanide stretching vibration (Figure 2A and S48 and S49). Upon vitrification, this band broadens markedly (from FWHM ∼20 cm^−1^ in the crystals to FWHM ∼30 cm^−1^ in the glasses), reflecting the distribution of CN–vibrational environments in the glassy states (Table S7). In addition, the bands at 817, 738, and 692 cm^−1^, corresponding to the C–H wagging vibrations of the naphthyl and phenyl rings of BINAP, also broaden significantly upon glass formation (Figures 2B and S48 and S49) [73]. The consistent band positions but broadened envelopes indicate preservation of molecular connectivity with increased structural disorder in the glasses.

X‐ray absorption spectroscopy (XAS) at the Cu *K*‐edge provides insight into the local coordination environment of the Cu^+^ centers in both crystalline and glassy states. The X‐ray absorption near‐edge structure (XANES) spectra exhibit very similar pre‐edge features, confirming that Cu remains monovalent and that its coordination environment is non‐centrosymmetric (Figures S50 and S51). The Fourier‐transformed extended X‐ray absorption fine structure (EXAFS) profiles display two peaks at 1.4 and 1.8 Å for the first coordination shell, corresponding to Cu─C/N and Cu─P coordination, respectively. The EXAFS spectra of the glasses can be well reproduced by fitting the data from 1–3 Å by using the single crystal structure of **CuRB** as the model, indicating that the local coordination geometry is preserved in the vitrified state (Figures 2C and S52–S56; Table S8–S13).

X‐ray total scattering measurements probe the short‐ and medium‐range structural correlations in the glasses relative to their crystalline analogues (Figure S57). Fourier transformation of the X‐ray total scattering structure factor *S*(*Q*) yields the pair distribution function (PDF), *G*(*r*), which provides atom–atom correlation information [74, 75]. The experimental PDF of **CuRB** matches well with the simulated profile derived from its crystal structure (Figures S58 and S59). The PDFs of the crystalline and glassy phases exhibit nearly identical features in the range 1–6 Å (Figures 2D and S60). Peaks at 1.9 and 2.3 Å correspond to Cu─C/N and Cu─P coordination bonds, consistent with the EXAFS results. The region beyond 2.3 Å is dominated by P⋯P, P⋯C, and C⋯C correlations of the BINAP ligands (Figures 2C,D and S60). A distinct peak at 5.0 Å corresponds to the distance between adjacent Cu atoms bridged by cyanide ligands, whereas weaker features near 6.0 Å arise from Cu⋯C distances from the copper(I) centers to the BINAP ligand. Collectively, these results demonstrate that vitrification preserves the primary coordination structure of the Cu_4_(CN)_4_(BINAP)_4_ complexes, while primarily disrupting long‐range molecular packing.

### 2.3 Photoluminescence and Chiroptical Properties

**FIGURE 3:**
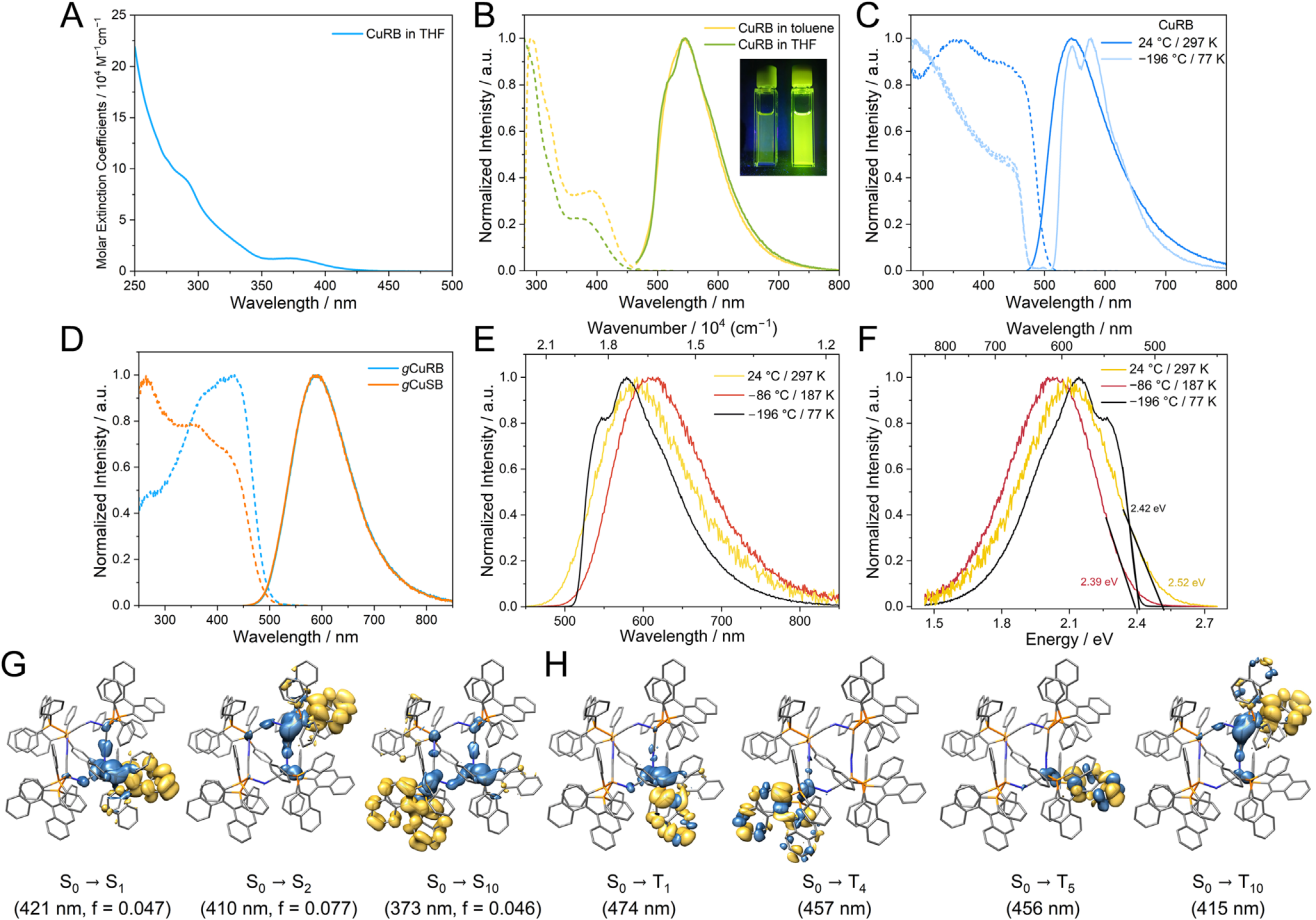
(A) Absorption spectrum of **CuRB** in THF (solid blue). (B) Normalized emission (*λ*_ex_ = 375 nm, solid) and excitation (*λ*_em_ = 550 nm, dashed) spectra in THF (green) and toluene (orange) of **CuRB** at room temperature; inset shows photographs of the luminescence in THF (left, 2.9 × 10^−5^ M) and toluene (right, 3.1 × 10^−5^ M) under inert atmosphere. (C) Normalized emission (*λ*_ex_ = 375 nm, solid) and excitation (*λ*_em_ = 550 nm, dashed) spectra of **CuRB** in the solid state at room temperature (dark blue) and at −196°C (77 K) (light blue) under inert atmosphere. (D) Normalized emission (*λ*_ex_ = 375 nm, solid) and excitation (*λ*_em_ = 590 nm, dashed) spectra of ***g*CuRB** and ***g*CuSB** in the solid state at room temperature under inert atmosphere. (E) Temperature dependent emission spectra of ***g*CuRB**. (F) Determination of the 0‒0 transition energy of the lowest emissive excited state of ***g*CuRB** at 24°C (297 K), −106°C (167 K), and –196°C (77 K), estimated from the high‐energy onset of the emission spectra. Calculated transition electron density differences for the (G) vertical spin‐allowed excitations S_0_→S_1_/S_2_/S_10_ and (H) vertical spin‐forbidden excitations S_0_→T_1_/T_4_/T_5_/T_10_ (D3BJ‐PBE0/ZORA/def2‐SVP, hydrogen atoms omitted for clarity; loss of electron density shown in blue, gain in yellow; f = oscillator strength).

We first studied the photophysical properties of **CuRB** in tetrahydrofuran (THF) solution. The UV‒vis absorption spectrum shows generally very broad bands, which can be attributed to the connection of four [Cu(CN)(BINAP)] units and a resulting high density of excited states (DOES) that are very similar in energy (Figure 3A, Table S14). Consequently, a broad lowest energy absorption band between *λ*_abs_ = 350–450 nm is observed and can be assigned to pseudohalide/metal‐to‐ligand charge transfer (XMLCT) of Cu(CN)→BINAP character (Figures 3G and S61). Our time‐dependent density functional theory (TD‐DFT) studies indicate that a multitude of vertical spin‐allowed singlet excitations of very similar nature leads to overlapping bands (Table S14), thus enhancing the observed extinction coefficients to appreciable values of *ε* ∼ 12,000 M^−1^ cm^−1^, although such CT bands in monocopper(I) complexes are typically of much lower oscillator strength [76]. We note that the steric congestion of the BINAP ligands prevents the formation of cuprophilic excited states typically observed in related luminescent copper(I) cyanide bis(phosphine) assemblies [77, 78]. The structureless high energy absorption with a maximum at 290 nm and high *ε* ∼ 92,000 M^−1^ cm^−1^ originates from various ligand‐centered (LC) ^1^ ππ* transitions of the PPh_2_ and naphthyl moieties.

The luminescence of **CuRB** in THF solution at room temperature exhibits a broad emission band peaking at *λ*_max_ = 545 nm, but with signs of vibrational progression, which suggests either the presence of a single CT state with significant LC character or two overlapping bands of simultaneously emitting states with the indicated nature (Figure 3B). We observed a bi‐exponential time‐resolved intensity decay with nearly identical contributions of *τ*_1_ and *τ*_2_ of 175 and 287 µs, respectively, which would support the latter assignment (Figure S62; Table 1). The average lifetime of 229 µs and moderate quantum yield of *Φ* = 0.07 provide a radiative rate constant *k*_r_ = 3.1 × 10^2^ s^−1^ that is typical for phosphorescence from the triplet excited state T_1_ in copper(I) complexes with moderate spin‐orbit coupling (SOC), where exothermic intersystem‐crossing (ISC) S_1_→T_1_ outcompetes prompt fluorescence S_1_→S_0_. Interestingly, the emission in less polar toluene is much more intense (*Φ* = 0.45) and the lifetimes change favorably toward the shorter component of the bi‐exponential decay, but the emission energy is insensitive to the polarity difference (Figures 3B and S63). Thus, the major effect of the 7‐fold luminescence enhancement stems from the greatly increased *k*_r_ = 2.8 × 10^3^ s^−1^ and to a lesser extent due to reduced *k*_nr_, that is, direct solvent coordination to the copper(I) center is not the cause of the observed low luminescence quantum yield in THF (Table 1). The change of *k*_r_ by one order of magnitude upon changing the solvent polarity is highly unusual, and the spectral appearance in toluene argues for less LC character in the emission as the vibrational shoulders are less pronounced.

**TABLE 1:** Photophysical properties of selected samples in this study.

Sample	*T* / °C	λ_em_ / nm	τ / µs	τ_av_ / µs	*Φ*	*k*_r_ (×10^3^) / s^−1^	*k*_nr_ (×10^3^) / s^−1^
**CuRB** (THF)	25	545	175 (0.52), 287 (0.48)	229	0.07	0.31	4.06
**CuRB** (Toluene)	25	545	143 (0.80), 232 (0.20)	161	0.45	2.8	3.41
**CuRB** (microcrystalline)	25	545	36 (0.60), 78 (0.39), 446 (0.01)	53.5	0.05	0.93	17.8
	−196	545	678 (0.37), 1594 (0.58), 3106 (0.05)	1327			
		577	1078 (0.50), 2042 (0.49), 5517 (0.01)	1576	0.61	0.39	0.25
		645	1386 (0.70), 2421 (0.29) 78289 (0.01)	1789			
***g*CuRB**	25	590	21 (0.90), 59 (0.09), 313 (0.01)	25.5	0.07	2.8	37.8
	−196	548	665.4 (0.41), 2227 (0.51), 6283 (0.08)	1895			
		578	929.6 (0.46), 2666.5 (0.49), 7320 (0.05)	2119	0.42	0.18	0.25
		630	1078.3 (0.37), 2797 (0.57), 7393 (0.05)	2412			
***g*CuSB**	25	591	21 (0.91), 59 (0.08), 342 (0.01)	24.8	0.08	3.23	37.2
	−196	547	1084 (0.57), 3161 (0.40), 9491 (0.03)	2187			
		580	1198 (0.57), 3331 (0.40), 10042 (0.03)	2305	0.43	0.19	0.25
		630	1410 (0.52), 3483 (0.46), 10456 (0.02)	2607			
***ds*CuRB** film	25	590	20 (0.90), 64 (0.09), 458 (0.01)	25.4			

Previous work on [Cu(carbazolate)(BINAP)] complexes showed that the vibrationally resolved ^3^LC state of the BINAP ligand is found between 490–800 nm with fundamental maxima at 500, 545, and 585 nm, which can overlap spectrally with MLCT or LLCT (LL = ligand‐to‐ligand) states in the same energy region [58]. While the ^3^ππ* LC state is generally insensitive to the environment polarity, the choice of the solvent can impact the energy of the CT states and thus change its contribution and SOC of the vertical transitions. Apparently, non‐polar toluene facilitates better energetic mixing of the CT states to mediate stronger SOC for spin‐forbidden emission processes in solution.

Many coinage metal complexes with d^10^ electron configuration featuring CT states exhibit TADF due to a small energy gap Δ(S_1_‐T_1_) < 0.25 eV [79, 80, 81, 82]. In such a scenario, sufficient energy is available at room temperature for endothermic reverse (r)ISC T_1_→S_1_ to occur and a Boltzmann equilibrium between the two states is established, allowing for delayed spin‐allowed fluorescence to bypass the slow phosphorescence process. With this strategy, ultrafast indirect radiative triplet excited state decay can be achieved with *k*_r_ up to 5 × 10^6^ and 2 × 10^6^ s^−1^ for gold(I) and copper(I) complexes, respectively, much faster than observed for traditional phosphorescence in commercial Ir^III^ or Pt^II^ emitter materials applied in OLEDs [82]. The comparatively small *k*_r_ values observed for **CuRB** in solution argue against TADF and in favor of phosphorescence at first glance (see below).

Microcrystalline powder of **CuRB** also shows a broad CT state emission band with *λ*_max_ = 545 nm, of which the efficiency (*Φ* = 0.05) and low *k*_r_ = 9.3 × 10^2^ s^−1^ resemble the behavior in THF solution (Figures 3C and S64; Table 1). Surprisingly, the rigid crystalline environment does not increase the luminescence quantum yield, but rather enhances the non‐radiative decay rate *k*_nr_ = 1.8 × 10^4^ s^−1^ in comparison to the solution state. Upon cooling to −196°C (77 K), a vibrational progression with enhanced *Φ* = 0.61 and elongated lifetimes of 1.3 to 1.8 ms is observed, typical for excited states of dominant ^3^ππ* LC character (Figure S65; Table 1). The CT luminescence maximum of the glassy state of ***g*CuRB** and ***g*CuSB** is bathochromically shifted to ca. 590 nm, although the band onset is nearly identical to the crystalline state, hinting at a larger excited state distortion (Figure 3D). Interestingly, *k*_nr_ is further enhanced to 3.7 × 10^4^ s^−1^ and *k*_r_ also experiences an increase to ca. 3 × 10^3^ s^−1^ at room temperature in comparison to the crystalline solid state (Figures S66 and S67, Table 1). The emission at −196°C (77 K) again originates from a ^3^ππ* state of the BINAP ligand with a very low *k*_r_ of only 2.4 × 10^2^ s^−1^ as observed in THF solution (Figures S68 and S69; Table 1).

A detailed steady‐state and time‐resolved variable temperature (VT) luminescence study of ***g*CuRB** disentangles the seemingly conflicting photophysical data observed in solution and in the solid state (Figures 3E,F and S70; Table S15). While at room temperature a luminescence lifetime of 26 µs is observed at *λ*_max_ = 590 nm, the band onset shifts bathochromically by ca. 115 meV (*λ*_max_ = 630 nm) with a much longer lifetime of 430 µs upon cooling to −106°C (167 K) (Table S15). Such a behavior is typical for TADF involving ^1/3^MLCT states [76]. Further cooling reveals the presence of the ^3^BINAP state with a lifetime in the millisecond regime, which is nearly isoenergetic with the ^3^MLCT state. ***ds*CuRB** exhibits similar photophysical properties as ***g*CuRB** (Figure S71–S73; Table S16).

**FIGURE 4:**
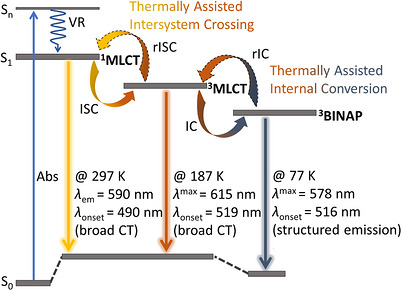
Franck–Condon diagram showing the proposed excited state decay of **CuRB** and **CuSB**.

The following excited state decay scheme is proposed for the luminescence of **CuRB** at room temperature (Figure 4). The ^3^LC state of the BINAP ligand thermally populates the ^3^MLCT state by reverse internal conversion (rIC), which then undergoes thermally assisted rISC to the ^1^MLCT state to allow for spin‐allowed fluorescence to occur. While the LC states are environment and polarity independent, the MLCT states are rather sensitive to such effects with regard to their energetic position. Consequently, and in contrast to classical coinage metal based TADF emitters [58, 76], the presence of an additional temperature‐dependent excited state equilibrium ^3^LC↔^3^MLCT in **CuRB** and **CuSB** leads to drastic environmental effects on *k*_r_ and *k*_nr_ (Table 1). Indeed, our TD‐DFT calculations performed at the geometry of the ground state S_0_ suggest the presence of triplet excited states of ^3^MLCT character and those of dominantly ^3^BINAP nature, which are very close in energy and of which the final energy after relaxation will depend on packing effects in the solid state and polarity effects of the environment (Figure 3H).

**FIGURE 5:**
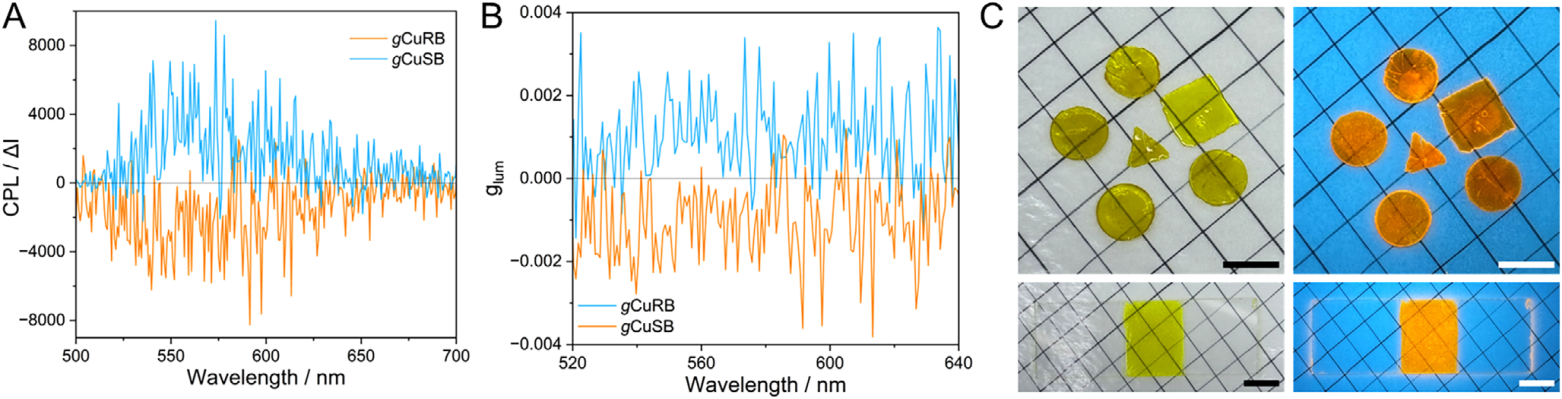
CPL spectra (A) and dissymmetry factors *g*_lum_ of the photoluminescence (B) of ***g*CuRB** and ***g*CuSB**. (C) Photographs of bulk glass monoliths with different shapes and a glass film of ***ds*CuRB**. Scale bar is 10 mm.

The chirality of the ground state of the metal complexes was examined by circular dichroism (CD). CD spectra of **CuRB** and **CuSB** feature mirrored Cotton effects at 253, 290, 330, and 380 nm, which is consistent with their opposite chirality (Figure S74). The opposite ellipticity of the MLCT band at 380 nm also suggests significant dissymmetry of the lowest energy excited states. The crystalline complexes exhibit a CPL response from 480 to 600 nm in the solid state, giving a luminescence dissymmetry factor (*g*_lum_) of 1 × 10^−3^ (Figures S75 and S76), which is lower than reported for binuclear [(BINAP)_2_Cu_2_(μ‐X)_2_] (X = Cl, I) complexes (∼1 × 10^−2^) [57, 60]. We attribute this difference in CPL response to the higher ^3^BINAP character of the dimer luminescence in comparison to our tetrameric arrangement, as well as to the different packing in the solid state as the crystalline dicopper(I) BINAP complexes dispersed in solvents show very similar *g*_lum_ values as observed in our systems. Importantly, CPL activity is retained upon vitrification. ***g*CuRB** and ***g*CuSB** showed opposite CPL responses from 500 to 700 nm with a dissymmetry of higher magnitude (*g*_lum_ = 2 × 10^−3^, Figure 5A,B). Considering that the intramolecular interactions change in the glass state in comparison to the crystalline samples as evident from the bathochromic luminescence shift, it is reasonable to assume that also the electronic and magnetic transition dipole moments differ and thus result in different CPL responses. The relatively mild melt‐quenching process, together with the high racemization barrier of the BINAP ligand, are critical for obtaining a CPL‐active metal–organic glass.

### 2.4 Fabrication of Bulk Glass Monoliths and Thin Films

The high processability and luminescent properties of the Cu_4_(CN)_4_(BINAP)_4_ glasses motivated the fabrication of transparent glass monoliths by thermal annealing. The desolvated glass ***ds*CuRB** was selected as a representative system as its solvent‐free character prevents bubble formation during molding at high temperature.

In contrast, the desolvation‐driven liquefaction of crystalline **CuRB** tends to trap residual solvent vapors, leading to bubble formation in the resulting bulk glasses (Figure 1C, insert). Powdered ***ds*CuRB** was first pressed into pellets of various shapes, including circular, triangular, and square forms (Figure S77). These pellets were then annealed slightly above *T*_g_ (here 220°C, corresponding to ∼1.04·*T*_g_) on a hot plate inside an Ar‐filled glovebox. Within a few minutes, the pressed powders coalesce into highly transparent, crack‐free glass monoliths (Figure 5C). The resulting glass monoliths emit intense yellow–orange photoluminescence under UV irradiation. In addition, uniform luminescent, transparent glass films with a thickness of ∼70 µm can be prepared by dispersing the powdered ***ds*CuRB** onto borosilicate glass substrates, followed by identical annealing treatment (Figure S78). Notably, the luminescence properties of ***ds*CuRB** are maintained in the glass films with *λ*_max_ = 590 nm and a tri‐exponential lifetime decay, of which the respective components are very similar (Table 1; Figures S79 and S80). The transmittance of the glass film reaches 65% in the region from 500 to 800 nm (Figure S81). Below 500 nm, the transmittance of the film rapidly decreases to 0%, consistent with the yellow color of ***ds*CuRB**.

### 2.5 Glass to Crystal Phase Transition by Vapor Treatment

Controlling glass‐to‐crystal phase transitions is essential for tuning the properties of functional glasses in optoelectronic applications [83, 84]. The glass powder ***g*CuRB** was treated with vapors (pure toluene, pure methanol, and mixed toluene/methanol) to investigate the recrystallization behavior and its impact on photoluminescence. For this purpose, a vial containing the glass powder was sealed inside a 120 mL bottle with 20 mL solvent; for the mixed solvent system, the toluene/methanol volume ratio was 1:4, identical to that used for crystal synthesis. The resulting samples are denoted ***g*CuRB‐Tol**, ***g*CuRB‐MeOH,** and ***g*CuRB‐Tol/MeOH**, respectively.

After 2 days of vapor exposure, ***g*CuRB‐MeOH** remains fully amorphous, whereas ***g*CuRB‐Tol** and ***g*CuRB‐Tol/MeOH** undergo crystallization (Figure S83). The PXRD pattern of ***g*CuRB‐Tol/MeOH** matches that of the crystalline complex **CuRB**, confirming successful reformation of the original phase. A ***ds*CuRB** glass film was also exposed to mixed toluene/MeOH vapor following the procedure described above. The initially transparent glass film became opaque and exhibited significant peeling and fragmentation, indicating substantial volume and morphology changes during the transition from the solvent‐free glassy to the solvent‐containing crystalline state. Hence, the fracture of the film can be attributed to the expansion of the material due to solvent uptake during crystallization (Figure S82). In contrast, ***g*CuRB‐Tol** displays a distinct PXRD pattern, suggesting that the complexes adopt a different molecular packing motif in the presence of only toluene as the guest species. The PXRD pattern of ***g*CuRB‐Tol** matches the simulated pattern of [Cu_4_(CN)_4_(*R*‐BINAP)_4_]·7toluene·MeOH (denoted as **CuRB‐Tol**), which was obtained by diffusion of a smaller amount of MeOH (relative to the synthesis of **CuRB**) into a toluene solution containing CuCN and *R*‐BINAP (Figure S84). **CuRB‐Tol** crystallizes in the monoclinic space group *P*2_1_ (Table S4, see experimental section). It exhibits the same molecular connectivity as **CuRB**; however, the altered crystallization conditions lead to differences in solvent content, as well as variations in the dihedral angle between the naphthalene units and the orientation of the phenyl rings of the BINAP ligands (Figure S85 and Table S17).

The photophysical properties further reflect these structural variations. ***g*CuRB‐Tol/MeOH** exhibits the same emission maximum at 546 nm as **CuRB**, but a shorter photoluminescent lifetime (18.60 vs 56.6 µs for **CuRB**), likely due to structural defects introduced during vapor‐induced recrystallization (Figures S86–S89; Table S16). In comparison, ***g*CuRB‐Tol** and ***g*CuRB‐MeOH** emit at 570 and 595 nm, respectively (Figure S86). Relative to the parent ***g*CuRB** (*λ*_em_ = 586 nm), the opposite shifts observed for ***g*CuRB‐Tol** (blueshift) and ***g*CuRB‐MeOH** (redshift) are attributed to differences in solvent polarity and the resulting non‐covalent interactions affecting the excited‐state environment.

## 3 Conclusion

We report homochiral metal−organic glasses composed of copper(I) cyanide complexes with *R*‐ and *S*‐BINAP ligands, which can be prepared either by a desolvation‐driven‐liquefaction/melt‐quenching process or by desolvation below *T*_g_. Structural analyses confirm that the molecular structure of the complexes is preserved upon vitrification. The resulting glasses exhibit intense yellow–orange TADF from ^1/3^MLCT states, which involves an unusual environment‐ and temperature‐dependent additional excited state equilibrium ^3^LC↔^3^MLCT at room temperature, while the robust BINAP ligands maintain structural chirality and CPL activity upon glass formation. The high fragility and processability of these materials enable the fabrication of luminescent glass thin films and bulk monoliths of diverse shapes by simple thermal annealing. Moreover, vapor‐induced glass‐to‐crystal transitions modulate the emission properties, demonstrating reversible control over the luminescent response. The combination of multiple vitrification methods, high processability, strong resistance to recrystallization in solvent‐free conditions, and tunable phase behavior establishes these CPL‐active metal–organic glasses as a promising platform for future optoelectronic applications.

## Author Contributions

**Zeyu Fan**: conceptualization, investigation, funding acquisition, writing – original draft, validation, formal analysis, visualization, writing – review and editing, methodology. **Indranil Sen**: investigation, writing – review and editing, visualization, validation, formal analysis. **Roman Pallach**: investigation, formal analysis. **Guo‐Qiang Li**: investigation, formal analysis. **Andreas Steffen**: writing – review and editing, funding acquisition, conceptualization, supervision, resources, formal analysis. **Sebastian Henke**: conceptualization, funding acquisition, writing – review and editing, supervision, resources, project administration, formal analysis.

## Conflicts of Interest

The authors declare no conflicts of interest.

## Supporting information




**Supporting File 1**: Details on materials synthesis, experimental, and computational methods; further single crystal and powder X‐ray diffraction (XRD) patterns; synchrotron radiation variable temperature PXRD (VT‐PXRD) patterns; X‐ray total scattering data; X‐ray absorption fine structure spectroscopy (XAFS); simultaneous thermogravimetric analysis (TGA) and differential scanning calorimetry (DSC) data; elemental analysis data; FTIR spectra; ^1^H, ^13^C, and ^31^P NMR spectra; photoluminescence data. The raw X‐ray total scattering data supporting this article are available at doi.org/10.15151/ESRF‐ES‐2123020263.
CCDC 2486564 (***meso*CuB**), 2486565 (**CuRB**), 2486566 (**CuSB**) and 2543814 (**CuRB‐Tol**) contain the supplementary crystallographic data for this paper. These data can be obtained free of charge via www.ccdc.cam.ac.uk/data_request/cif, or by emailing data_request@ccdc.cam.ac.uk, or by contacting The Cambridge Crystallographic Data Centre, 12 Union Road, Cambridge CB2 1EZ, UK; fax: +44 1223 336033.
The authors have cited additional references within the Supporting Information [74, 75, 85–101].

**Supporting File 2**: anie72529‐sup‐0002‐cif.zip.

## Data Availability

The data that support the findings of this study are available in the Supporting Information of this article.
