# Supplementary material for: Circularly Polarized Luminescent and Melt‐Processable Copper(I)‐Organic Glasses Based on 2,2′‐Bis(diphenylphosphino)‐1,1′‐binaphthyl
Source: Angew Chem Int Ed Engl. 2026 May 7;65(26):e9766332. doi: 10.1002/anie.9766332 (PMC13285472; doi:10.1002/anie.9766332)
Supplement: Supplementary file 1 — Supporting File 1: Details on materials synthesis, experimental, and computational methods; further single crystal and powder X‐ray diffraction (XRD) patterns; synchrotron radiation variable temperature PXRD (VT‐PXRD) patterns; X‐ray total scattering data; X‐ray absorption fine structure spectroscopy (XAFS); simultaneous thermogravimetric analysis (TGA) and differential scanning calorimetry (DSC) data; elemental analysis data; FTIR spectra; 1H, 13C, and 31P NMR spectra; photoluminescence data. The raw X‐ray total scattering data supporting this article are available at doi.org/10.15151/ESRF‐ES‐2123020263. CCDC 2486564 ( mesoCuB), 2486565 (CuRB), 2486566 (CuSB) and 2543814 (CuRB‐Tol) contain the supplementary crystallographic data for this paper. These data can be obtained free of charge via www.ccdc.cam.ac.uk/data_request/cif, or by emailing data_request@ccdc.cam.ac.uk, or by contacting The Cambridge Crystallographic Data Centre, 12 Union Road, Cambridge CB2 1EZ, UK; fax: +44 1223 336033. The authors have cited additional references within the Supporting Information [74, 75, 85–101]. [file ANIE-65-e9766332-s002.pdf]

Supporting information for

Circularly Polarized Luminescent and Melt-Processable Copper(I)-Organic Glasses based on 2,2′-Bis(diphenylphosphino)-1,1′-binaphthyl

Zeyu Fan*, Indranil Sen, Roman Pallach, Guo-Qiang Li, Andreas Steffen*, Sebastian Henke*

Anorganische Chemie, Fakultät für Chemie und Chemische Biologie, Technische Universität Dortmund, Otto-Hahn-Straße 6, 44227 Dortmund, Germany

E-mail: zeyu.fan@tu-dortmund.de; andreas.steffen@tu-dortmund.de; sebastian.henke@tu-dortmund.de

Experimental Section

1. Materials and experimental methods

All reagents and chemicals were obtained from commercial sources and used without further purification. CuCN (99%) was purchased from Sigma-Aldrich. (*R*)-2,2'-Bis(diphenylphosphino)-1,1'-binaphthalene (*R*-BINAP, 99%, 99.5%ee) and (*S*)-2,2'-Bis(diphenylphosphino)-1,1'-binaphthalene (*S*-BINAP, 99%, 99.5%ee) were purchased from BLDpharm. MeOH and Toluene with HPLC grade were purchased from Fisher Scientific.

Synthesis of $[Cu_4(CN)_4(R\text{-BINAP})_4]\cdot 2toluene\cdot xH_2O$ (CuRB)

CuCN (0.5 mmol) and *R*-BINAP (0.5 mmol) were added to 30 mL of toluene. The mixture was sonicated until the solid dissolved, giving a yellow solution. 120 mL of MeOH was added dropwise to the above solution with stirring. The mixture was stirred for 1 day at room temperature to give microcrystalline powder of **CuRB** (yield: 285 mg, 72.3% based on Cu).

Single crystals of **CuRB** were obtained by a slow diffusion method. CuCN (0.1 mmol) and *R*-BINAP (0.1 mmol) were dissolved in 10 mL of toluene by sonication. 0.5 mL of the solution was then layered with 2 mL of MeOH. Crystals suitable for single crystal diffraction were obtained after a few days.

Synthesis of $[Cu_4(CN)_4(S\text{-BINAP})_4]\cdot 2toluene\cdot xH_2O$ (CuSB)

The synthetic processes for microcrystalline powder and single crystals were the same as for **CuRB** while using *S*-BINAP instead of *R*-BINAP (the yield of the microcrystalline powder synthesis was 300 mg, 77.5% based on Cu).

Synthesis of $[Cu_4(CN)_4(BINAP)_4]\cdot 3toluene$ (*meso*CuB)

Single crystals of ***meso*CuB** were obtained by a slow diffusion method. CuCN (0.1 mmol) and *R*-BINAP or *S*-BINAP (0.1 mmol) were dissolved in 10 mL of toluene by sonication, giving solution **A** (with *R*-BINAP) or **B** (with *S*-BINAP). Crystals suitable for single crystal diffraction were fabricated by layering 0.2 mL of solution **A** on 0.2 mL of solution **B** with 0.4 mL of toluene in between as buffer. Crystals were ground into powder for thermal analysis.

Synthesis of $[Cu_4(CN)_4(R\text{-BINAP})_4]\cdot 7toluene\cdot MeOH$ (CuRB-Tol)

The synthetic process for single crystals was the same as for **CuRB,** except that 0.5 mL of the stock solution was layered with 1 mL of MeOH (instead of 2 mL used for **CuRB**).

Melt-quenched glass formation

Microcrystalline powders of **CuRB** and **CuSB** were heated in a DSC machine (heating rate 10 °C·min^{-1}, maximum temperature 230 °C) under N_2 atmosphere to give ***g*CuRB** and ***g*CuSB**.

Glass formation by desolvation

Microcrystalline powders of **CuRB** and **CuSB** were heated to 190 °C under vacuum for 1 day to give ***ds*CuRB** and ***ds*CuSB**.

2. Physical characterization

Single crystal X-ray diffraction (SCXRD) was carried out on a Bruker D8 Venture diffractometer using MoKα or CuKα radiation. An Oxford cryostream (N_2) was used for cooling the sample to 100 K.

Powder X-ray diffraction (PXRD) patterns were recorded on a Bruker D8 diffractometer in Bragg−Brentano geometry with CuKα radiation in the range from 4° to 50° (step size 0.02°). Samples were deposited on a zero-background single-crystalline silicon holder.

Variable temperature (VT) PXRD patterns were recorded at beamline 9 of DELTA (Dortmund, Germany) with a monochromatic X-ray beam (λ = 0.4592 Å) using a MAR345 image plate detector. Samples were sealed in quartz glass capillaries and placed on a Linkam heating stage. Temperature calibration of the hot stage was performed by reference PXRD measurements of α-quartz.

Simultaneous thermogravimetric and differential thermal analysis (TG/DTA) measurements were conducted on a SDT650 instrument from

TA Instruments (USA) under a constant N_2 flow (6 L h^{-1}). Heating rate is 10 °C·min^{-1}.

Differential scanning calorimetry (DSC) measurements were conducted on a DSC25 instrument from TA Instruments (USA) under N_2 atmosphere using aluminium pans with a pinhole for the sample compartment and heating/cooling rates between ±5 and ±25 °C min^{-1}. Heat capacity measurements were performed with temperature-modulated (m)DSC, where a sinusoidal temperature modulation with an amplitude of ±1 °C and a modulation period of 120 s was overlaid on a linear heating ramp with an average heating rate of 2 °C min^{-1}. Baseline and sapphire reference scans were collected before the sample scan using the same temperature program. The total heat flow is composed of two components. One component is a function of the sample's heat capacity and rate of temperature change, and the other is a function of temperature and time:

$$\frac{dH}{dt} = C_p\beta + f(T,t)$$

where $\frac{dH}{dt}$ is total heat flow, C_p is heat capacity, β is heating rate, $f(T,t)$ is the heat flow from kinetic (temperature and time dependent) processes. mDSC is able to determine the total, as well as these two individual heat flow components, from the time-dependent heat flow signal, which arises due to the time-dependent modulation of the temperature ramp.

Fourier-transform infrared (FTIR) spectroscopy of the mid-IR range ($\tilde{v}$ = 400 cm^{-1} - 4000 cm^{-1}) and far-IR range ($\tilde{v}$ = 100 cm^{-1} - 700 cm^{-1}) was carried out on a PerkinElmer Spectrum 3 FT-IR spectrometer from Perkin Elmer in reflection mode using a GladiATR diamond ATR (attenuated total reflectance) unit from Pike Technologies.

X-Ray total scattering data were collected at beamline ID22 at European Synchrotron Radiation Falicity (ESRF, France) using a monochromatic X-ray beam (λ = 0.17715143 Å, 70keV). The finely ground samples were placed in 1mm (outer diameter) quartz glass capillaries. Background

subtraction was performed with scattering data collected from an empty capillary. Data were collected for the background, empty capillary, and all samples to a Q_{max} of 23 Å^{-1}. The total scattering data $I_m(Q)$ was processed by using PDFgetX3 software.[1] Background subtraction was performed, followed by extraction of the coherent scattering intensity $I_c(Q)$ using an automated polynomial correction. The structure factor $S(Q)$ was then calculated from $I_c(Q)$ and converted into the pair distribution function $G(r)$ via a sine Fourier transform[2-3]:

$$G(r) = \left(\frac{2}{\pi}\right)\int_{Q_{min}}^{Q_{max}} Q[S(Q)-1]\sin(Qr)\,dQ$$

X-ray absorption spectroscopy (XAS) was performed at beamline 10 of DELTA (Dortmund, Germany). All samples were measured in Ar at 25 °C. The raw absorption spectra were processed using the Athena software module of Demeter software packages[4], including pre-edge background subtraction and normalization to the edge step, resulting in normalized $\mu(E)$ spectra. In order to determine local atomic structure around Cu atom, the extended X-ray absorption fine structure (EXAFS) analysis was performed. The analysis procedure began with extracting EXAFS function $\chi(E)$, by removing the smooth atomic background $\mu_0(E)$ from normalized $\mu(E)$, according to:

$$\chi(E) = \frac{\mu(E) - \mu_0(E)}{\Delta\mu_0}$$

where $\Delta\mu_0$ is the edge step. The energy axis was then converted to photoelectron wavevector space (k-space) using:

$$k = \sqrt{\frac{2m(E-E_0)}{\hbar^2}}$$

where E_0 is the absorption edge energy, m is the electron mass, and $\hbar$ is the reduced Planck constant. The resulting $\chi(k)$ function, in the k range from 3 to 13 Å^{-1}, was weighted by k^3 to enhance high-k oscillations and subsequently Fourier transformed to obtain the real-space structure function $\chi(R)$:

$$\chi(R) = \frac{1}{\sqrt{2\pi}}\int_{k_{min}}^{k_{max}} \mathrm{W}(k)k^2\,\chi(k)e^{2ikR}\mathrm{d}k$$

where $W(k)$ is a window function applied to reduce Fourier transform artifacts. The resulting $\chi(R)$ reflects the radial distribution of neighboring atoms around the absorbing Cu atom, modulated by photoelectron scattering phase shifts and amplitude functions.

Quantitative fitting of the EXAFS data was also performed using the Artemis module of the software package Demeter to extract local structural parameters.[4] The k^3-weighted $\chi(R)$ spectrum was fitted over the R-range of 1.0 to 3.5 Å using theoretical scattering paths generated by the program FEFF, based on the single-crystal structure of **CuRB**. The fitting results are summarized in **Table S6-11**.

Nuclear magnetic resonance (NMR) spectroscopy was performed with Bruker DPX-300, DPX 500 or Agilent DD2 500 spectrometers using THF-d_8 (0.6 mL) as solvent. The data were processed with the MestReNova software. For **diffusion ordered spectroscopy (DOSY)**, samples were also dissolved in THF-d_8. Data was processed by TopSpin (v4.1.4) and dynamic center software. The Stokes radius r_h was calculated by Stokes–Einstein–Sutherland equation as shown below:

$$D = \frac{k_B T}{6\pi\eta r_h}$$

Where D is the diffusion coefficient of the spherical particle, k_B is Boltzmann constant, T is the absolute temperature, η is the dynamic viscosity of solvent.

The fitting was done by using the software Dynamics Center from Bruker with the following equation:

$$I(q) = I_0 \cdot e^{-q^2 D\left(\Delta - \frac{\delta}{3}\right)}$$

Where q is gradient strength parameter, I is the observed NMR signal intensity, I_0 is the reference signal intensity, Δ is diffusion time, δ is the duration of the gradient pulse.

UV-vis spectra were performed on an Agilent Cary 5000 spectrophotometer using standard 1 cm pathlength quartz cells.

Excitation and emission spectra were recorded on an Edinburgh Instrument FLS1000 spectrometer, equipped with a 450 W Xenon arc lamp. The emission was collected at right angle to the excitation source with the emission wavelength selected using a double grated monochromator for the excitation and emission pathways and detected by and a red-sensitive photomultiplier (PMT-980) as detector.

Luminescence lifetimes were measured using a µF2 pulsed 60 W Xenon microsecond flashlamp, with a repetition rate of 100 Hz and a multichannel scaling (MCS) module or with VPLED-450 (449.6 nm with 37 mW in CW mode), with 100 ns pulse width and an MCS module, depending on the time range. The low temperature experiments were performed using liquid nitrogen cooled OptistatDN-V cryostat from Oxford Instruments.

Quantum yields were measured by use of an integrating sphere or an EI cryosphere (integrating sphere mounted on an Oxford Instruments Microstat N_2, coupled to the spectrometer with optical fibers for excitation and emission) with an Edinburgh Instrument FLS1000 spectrometer. Solutions state quantum yields were determined on a Hamamatsu Quantaurus system (model type C11347-11), equipped with a 150 W Xenon arc lamp. The error margin is 0.02.

Polarization-sensitive measurements were carried out with Edinburgh Instrument's CPL option for the FLS1000 (linear polarizer in the excitation beam, photoelastic modulator (PEM) in the emission pathway). The linear polarizer is kept at a 45° angle to the quarter-wave plate in the PEM, which is rotated by 90° with a switching frequency of 50 kHz.

DFT and TD-DFT calculations were performed with the ORCA 6.0.1 program suite with tight SCF convergence criteria.[5] Geometry optimizations (gas-phase) omitting the respective anions were carried out with the BP86 functional[6] as implemented in ORCA and a frequency analysis ensuring that the optimized structures correspond to energy minima. The def2-SVP[7] basis set was used for all atoms together with the auxiliary basis set SARC/J[8-12] in order to accelerate the computations within the framework of RI approximation. Relativistic effects

were accounted for by employing the ZORA method as implemented for the def2-SVP basis set in Orca and by employing the SARC-ZORA-TZVP[9, 13] basis set for the metal atoms. Van der Waals interactions have been considered by an empirical dispersion correction (Grimme-D3BJ).[14-15] TD-DFT calculations for the first 40 singlet and 15 triplet excited states were performed with the same basis sets, but the PBE0 functional[16-17] was used. Representations of electronic transition differences at isovalues of 0.005 were produced with orca_plot as provided by ORCA 6.0.1 and with Chimera.[18]

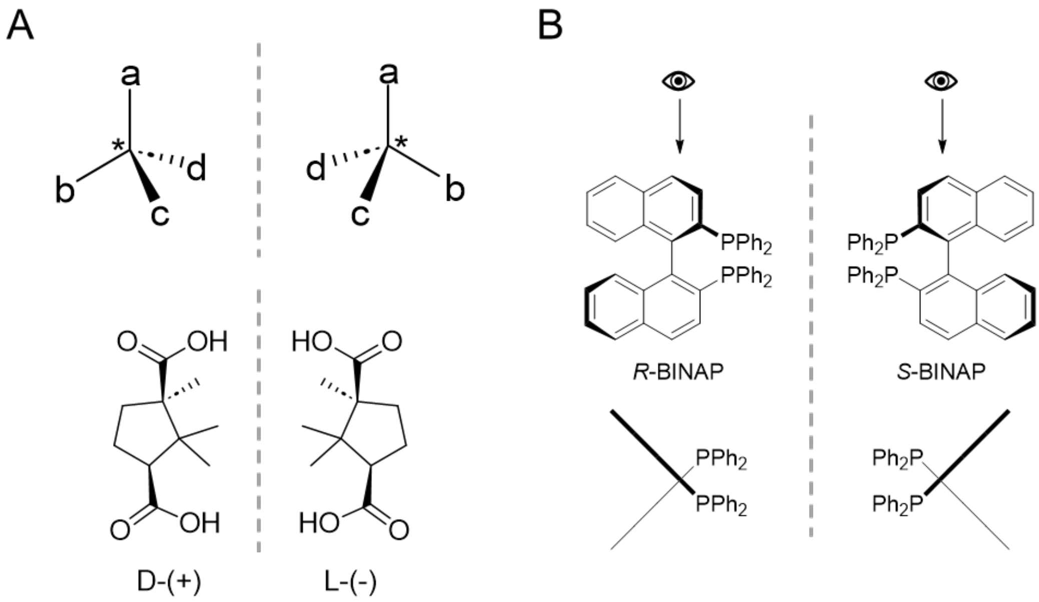

Figure S1. (A) Conventional chiral ligands for synthesizing metal-organic materials. a, b, c, d represents different substituents to achieve central chirality of the molecule. D-(+)- and L-(-)-camphoric acid is shown as an example. (B) Axial chirality of BINAP. The chirality originates from the different spatial orientations between the two naphthalene planes

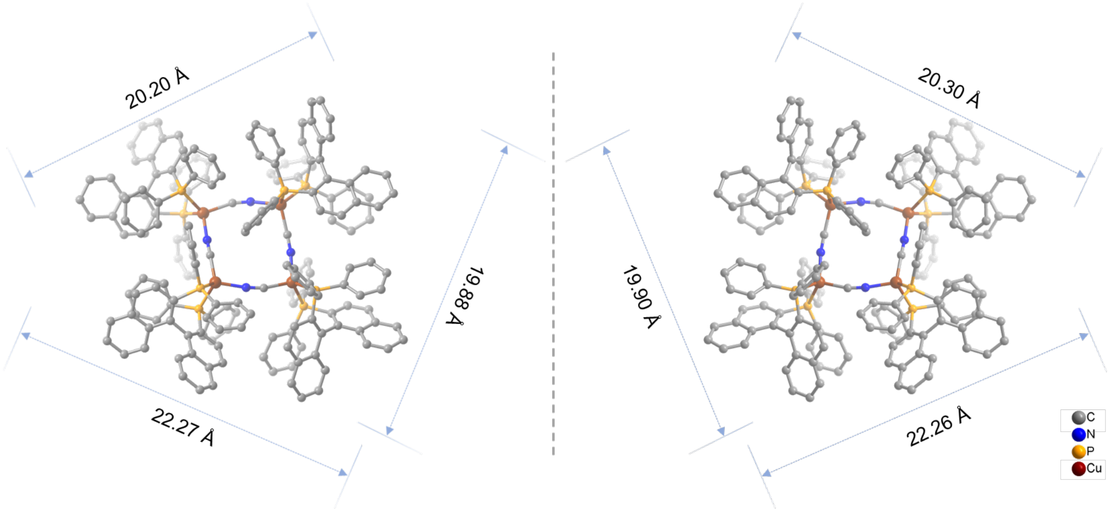

Figure S2. Measured molecular size of **CuRB** (left) and **CuSB** (right) from crystal structures. H atoms and guest molecules are omitted for clarity.

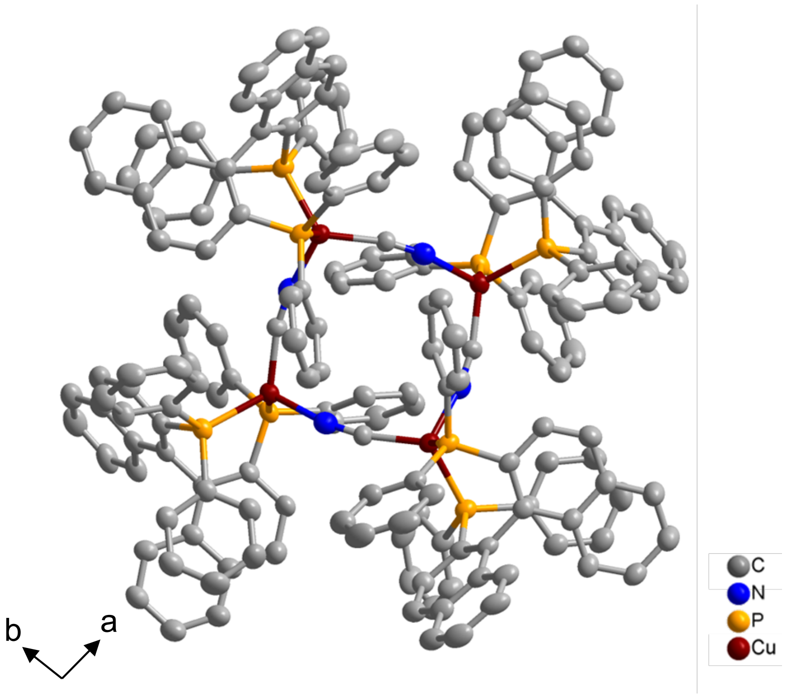

Figure S3. Single crystal structure of ***meso*CuB** (ellipsoids shown at 50% probability). Guest toluene molecules and hydrogen atoms are omitted for clarity.

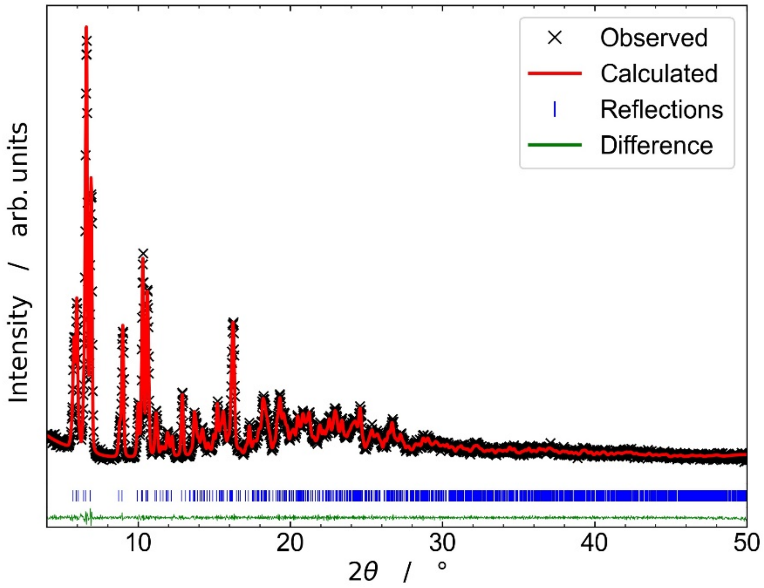

Figure S4. Pawley fit performed on the PXRD pattern of as-synthesized microcrystalline **CuRB**. The corresponding crystallographic data can be found in Table S4.

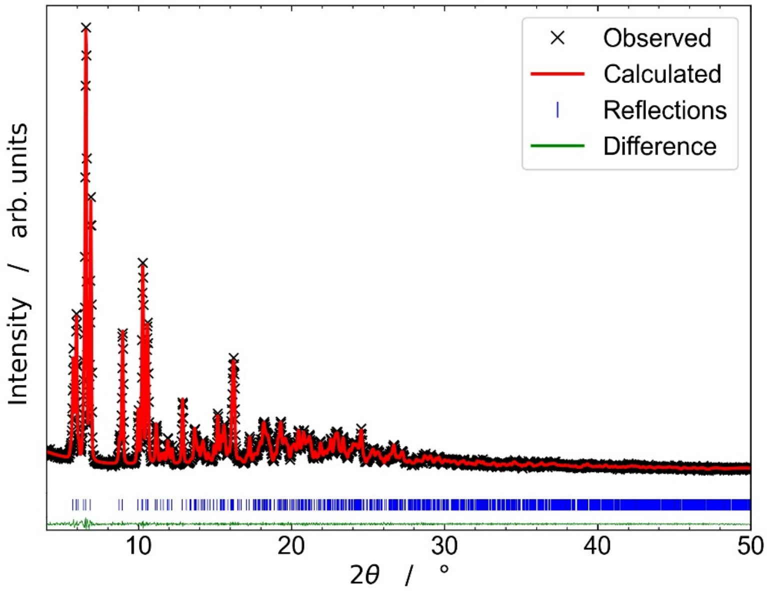

Figure S5. Pawley fit performed on the PXRD pattern of as-synthesized microcrystalline **CuSB**. The corresponding crystallographic data can be found in Table S4.

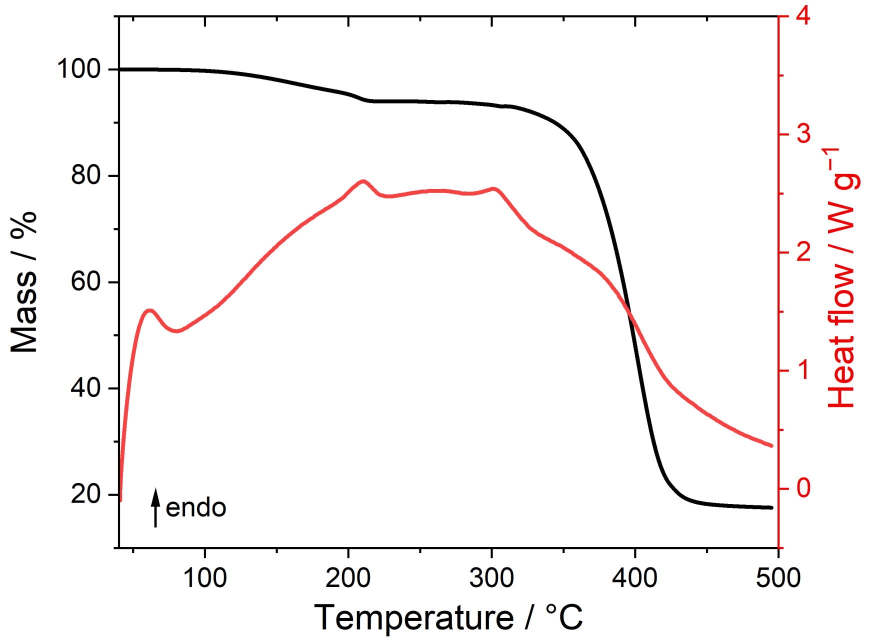

Figure S6. TGA profile of **CuRB**.

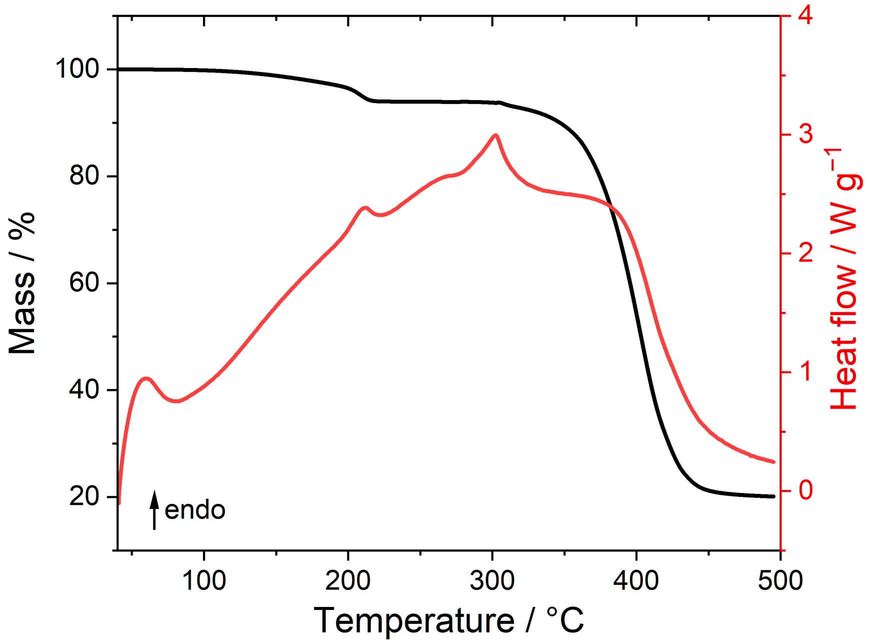

Figure S7. TGA profile of **CuSB**.

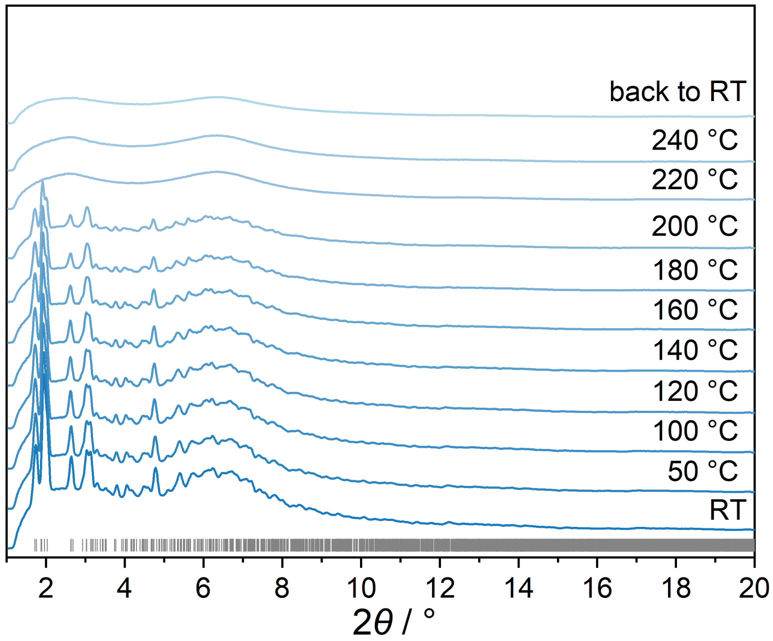

Figure S8. VT-PXRD of **CuRB** from RT to 240 °C, λ = 0.4592 Å.

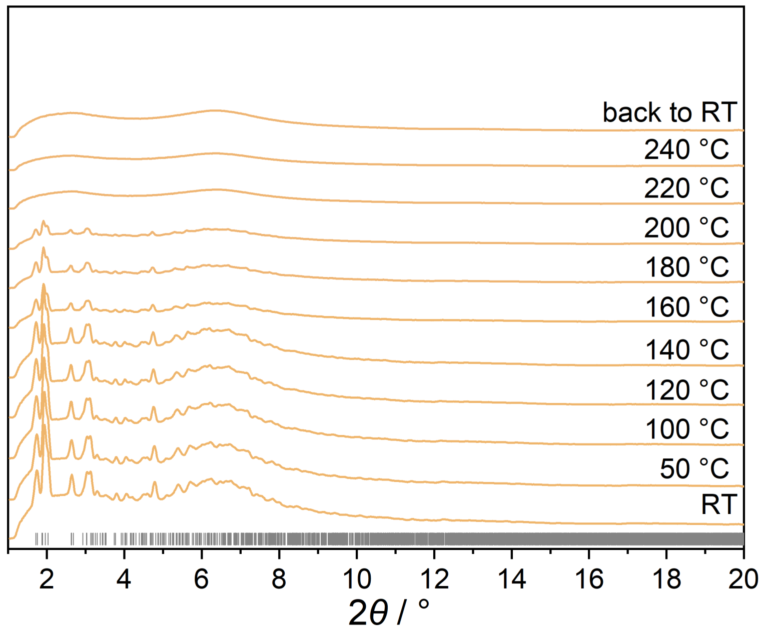

Figure S9. VT-PXRD of **CuSB** from RT to 240 °C, λ = 0.4592 Å.

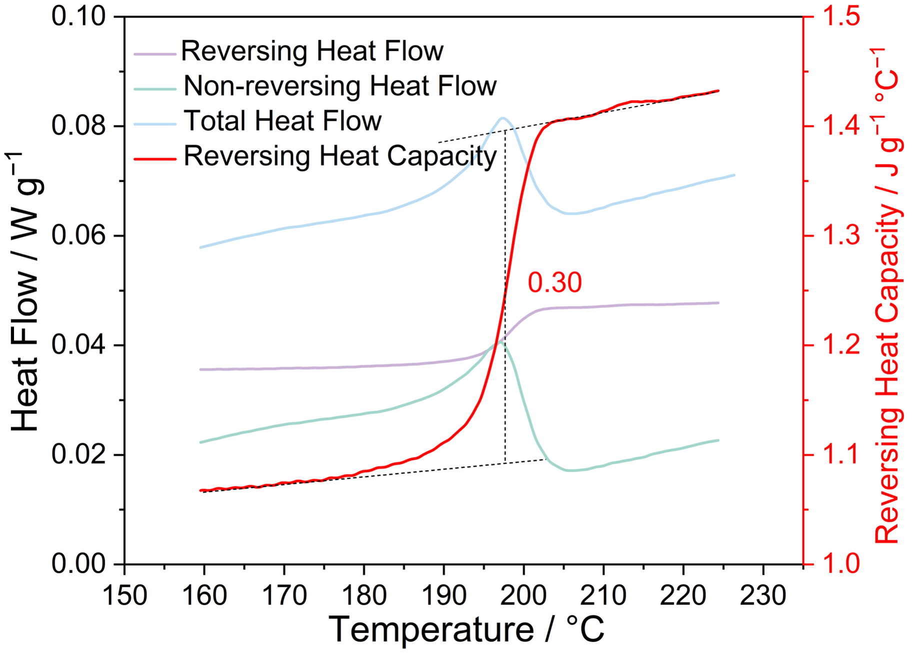

Figure S10. mDSC profiles of **CuRB**. Heating rate is 2 °C·min^{-1}.

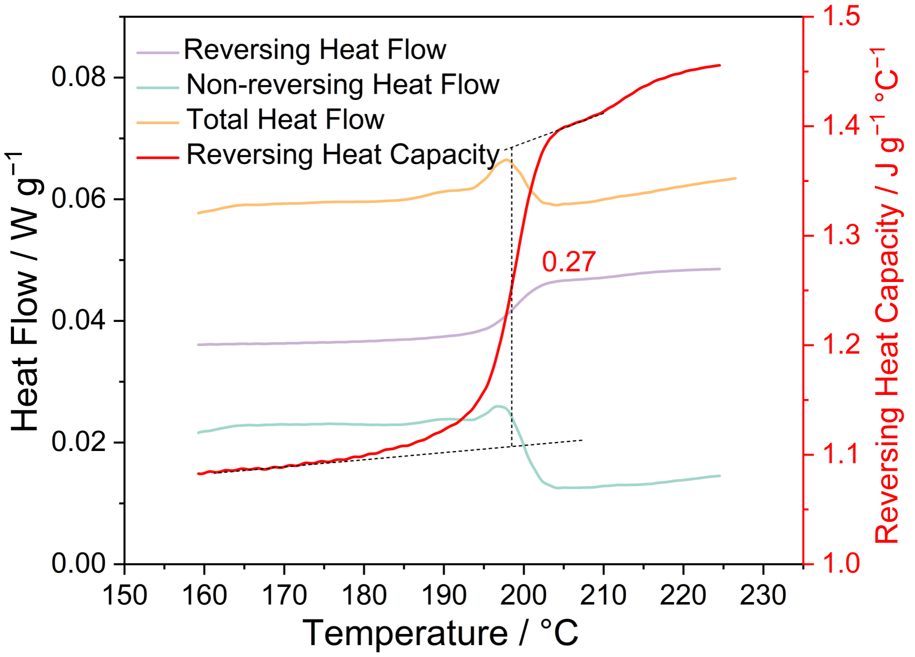

Figure S11. mDSC profiles of **CuSB**. Heating rate is 2 °C·min^{-1}.

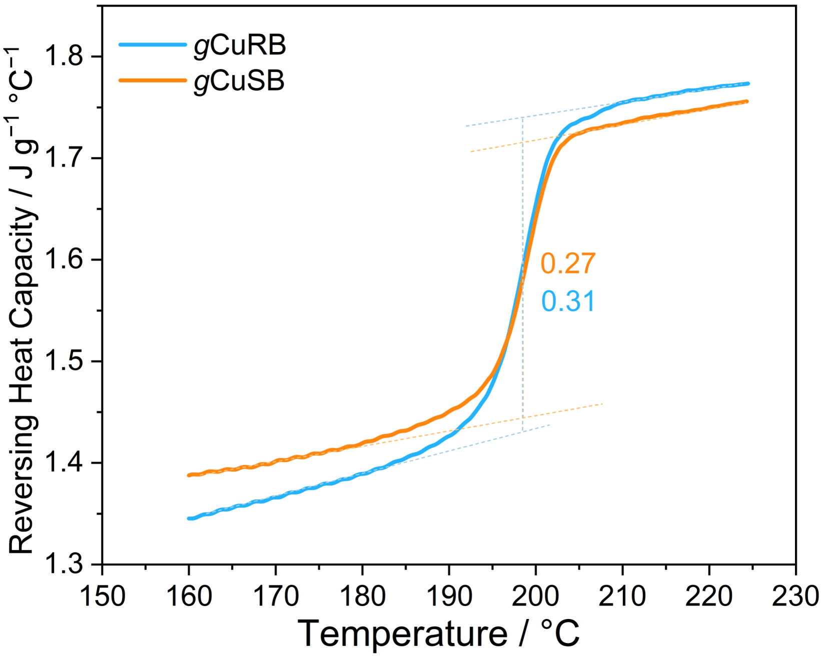

Figure S12. Heat capacity data of ***g*CuRB** and ***g*CuSB** extracted from the mDSC measurements. The step is the heat capacity change ΔC_p at the glass transition. Heating rate is 2 °C·min^{-1}.

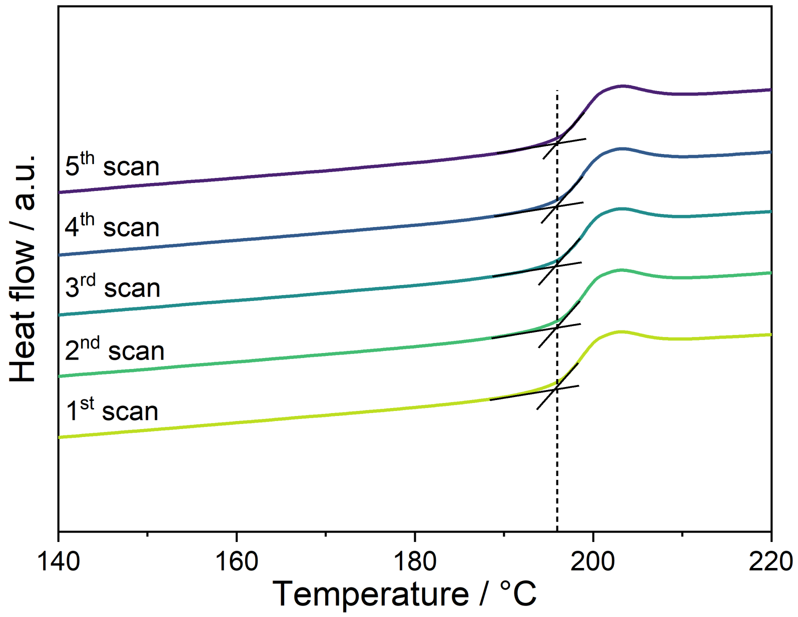

Figure S13. Five consecutive DSC upscan profiles of ***g*CuRB** showing the stability of T_g. Heating rate is 10 °C·min^{-1}.

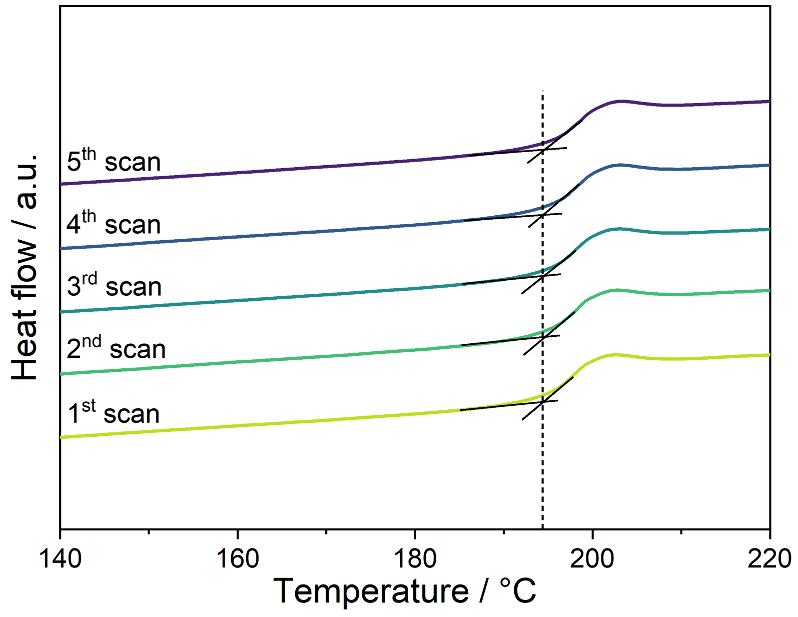

Figure S14. Five consecutive DSC upscan profiles of ***g*CuSB** showing the stability of T_g. Heating rate is 10 °C·min^{-1}.

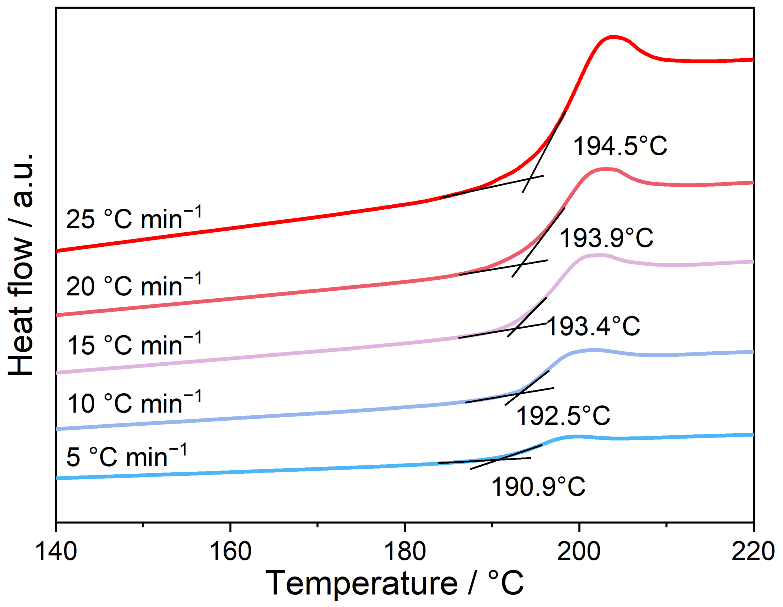

Figure S15. DSC profiles of ***g*CuSB** with heating rate from 5 to 25 °C·min^{-1}.

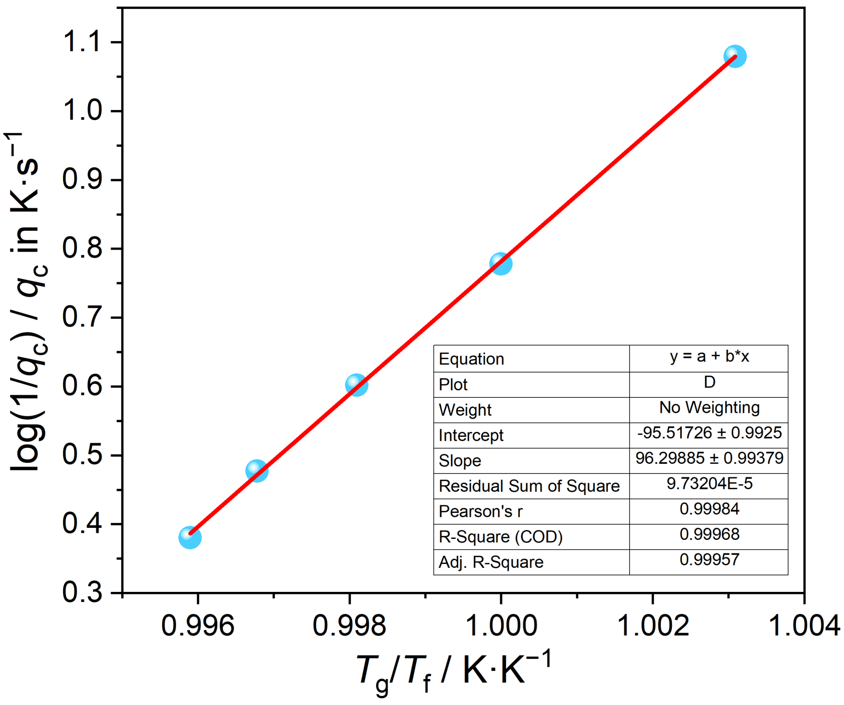

Figure S16. Determination of the calorimetric fragility index *m* of ***g*CuRB** by a linear fit of log(1/q_c) against T_g/T_f (q_c = cooling rate of the DSC scan prior to the upscan for determination of T_f; the heating rate of the corresponding upscan is identical to q_c).

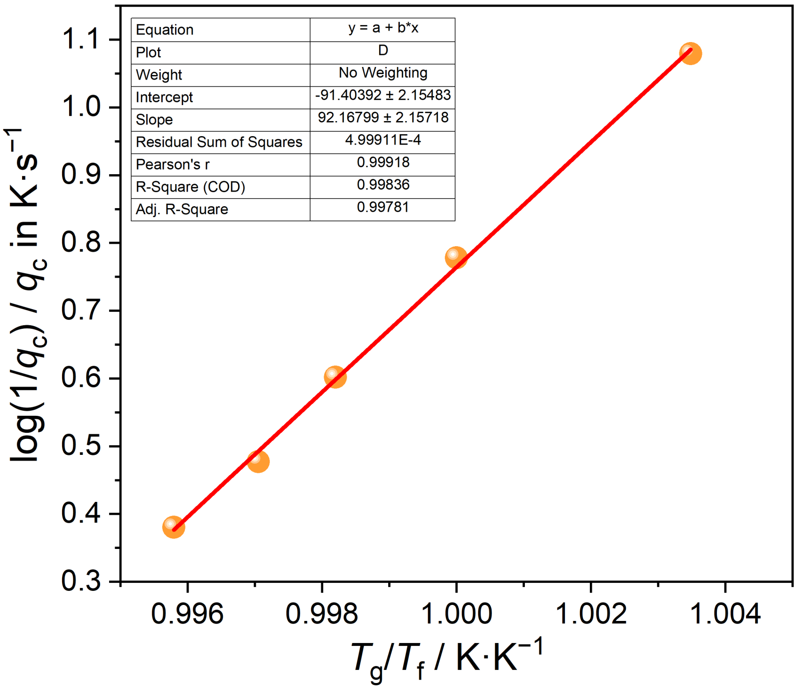

Figure S17. Determination of the calorimetric fragility index *m* of ***g*CuSB** by a linear fit of log(1/q_c) against T_g/T_f (q_c = cooling rate of the DSC scan prior to the upscan for determination of T_f; the heating rate of the corresponding upscan is identical to q_c).

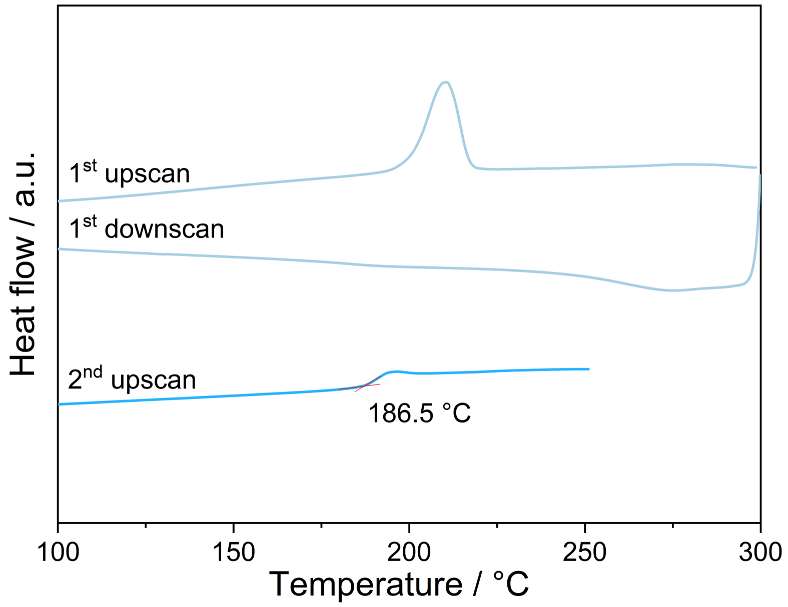

Figure S18. DSC profiles of **CuRB** up to 300 °C. The second upscan indicates the T_g at 186.5 °C.

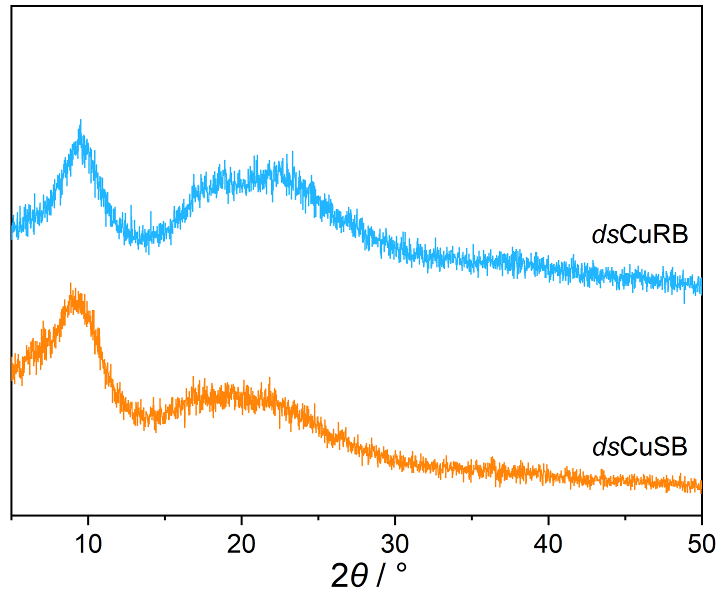

Figure S19. PXRD patterns of ***ds*CuRB** and ***ds*CuSB**.

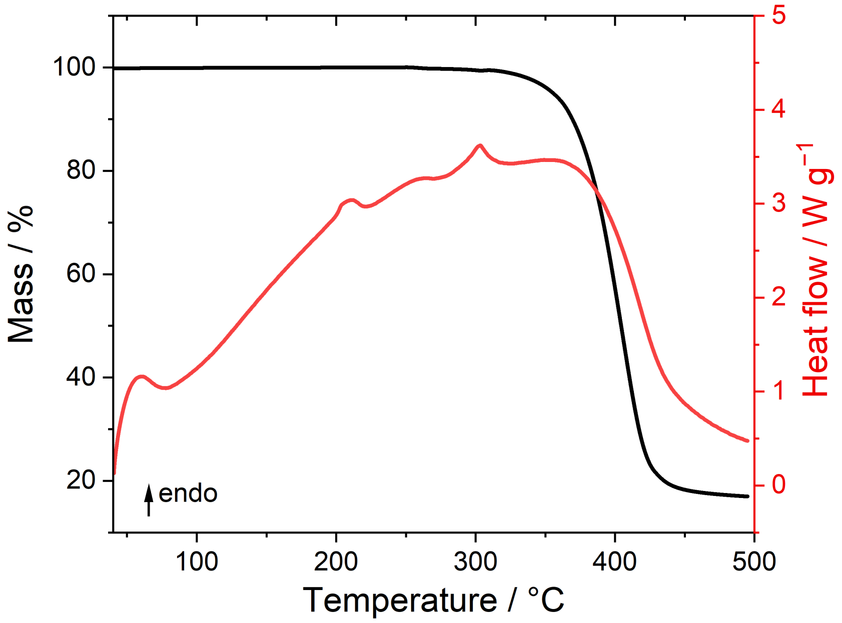

Figure S20. TGA profile of ***ds*CuRB**.

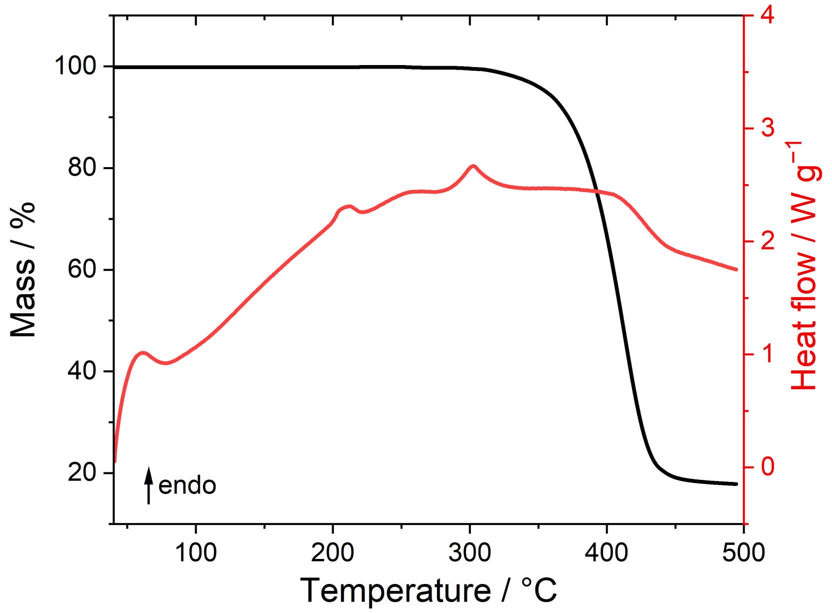

Figure S21. TGA profile of ***ds*CuSB**.

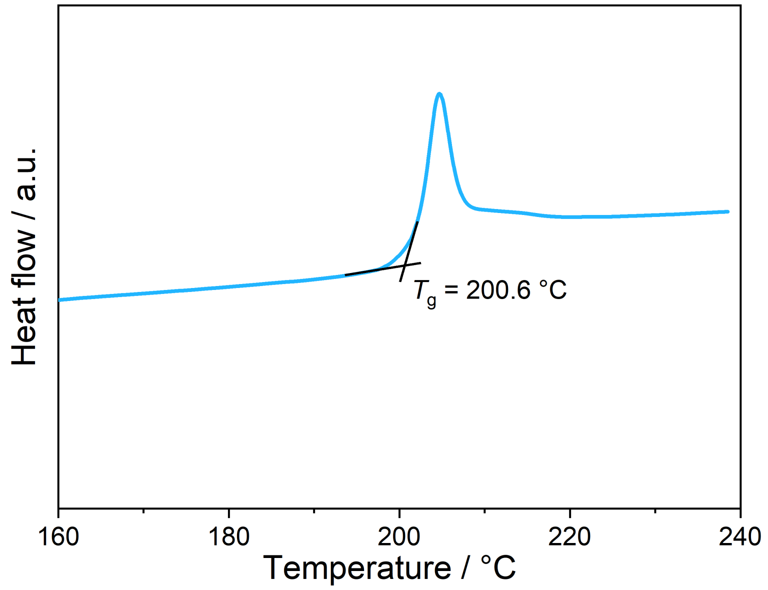

Figure S22. DSC profile of ***ds*CuRB**. Heating rate is 10 °C·min^{-1}. The endothermic overshoot following T_g arises from structural relaxation of the supercooled liquid, reflecting enthalpy recovery of the relaxed glass obtained by desolvating **CuRB** at 190 °C for 24 h.

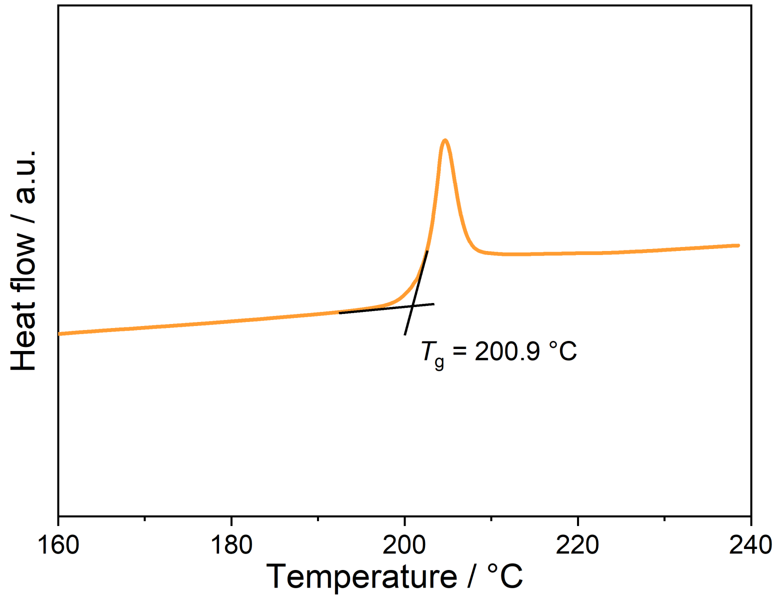

Figure S23. DSC profile of ***ds*CuSB**. Heating rate is 10 °C·min^{-1}. The endothermic overshoot following T_g arises from structural relaxation of the supercooled liquid, reflecting enthalpy recovery of the relaxed glass obtained by desolvating **CuSB** at 190 °C for 24 h.

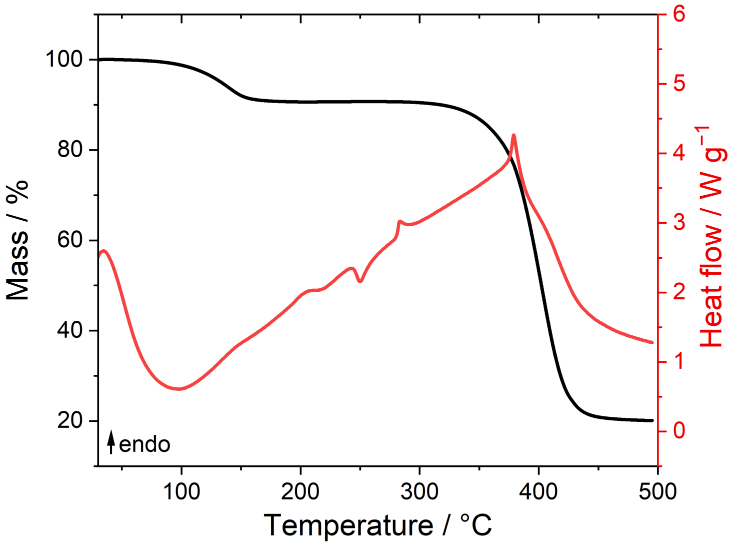

Figure S24. TGA profile of ***meso*CuB**.

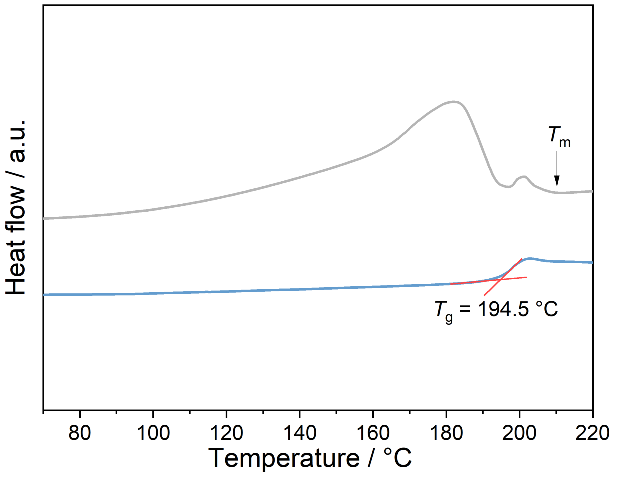

Figure S25. DSC profile of ***meso*CuB** .

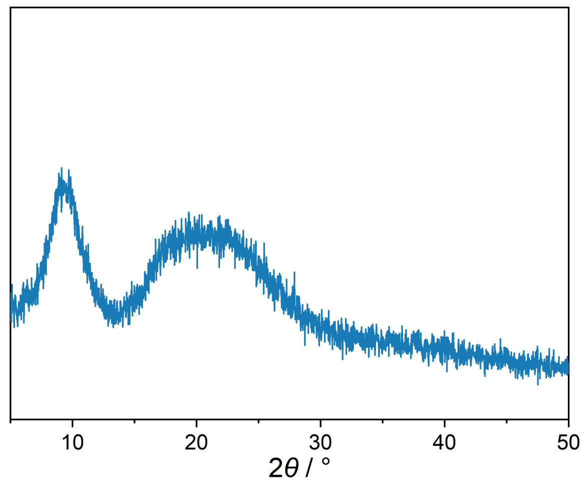

Figure S26. PXRD of ***gmeso*CuB** .

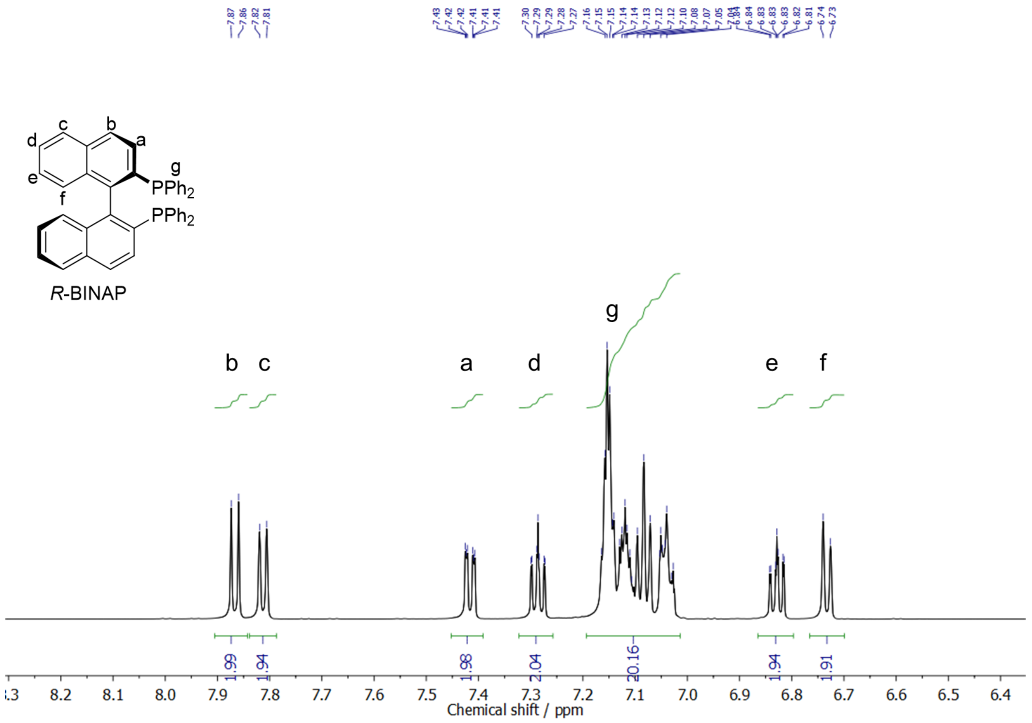

Figure S27. ^{1}H NMR spectrum of *R*-BINAP dissolved in THF-d_8.

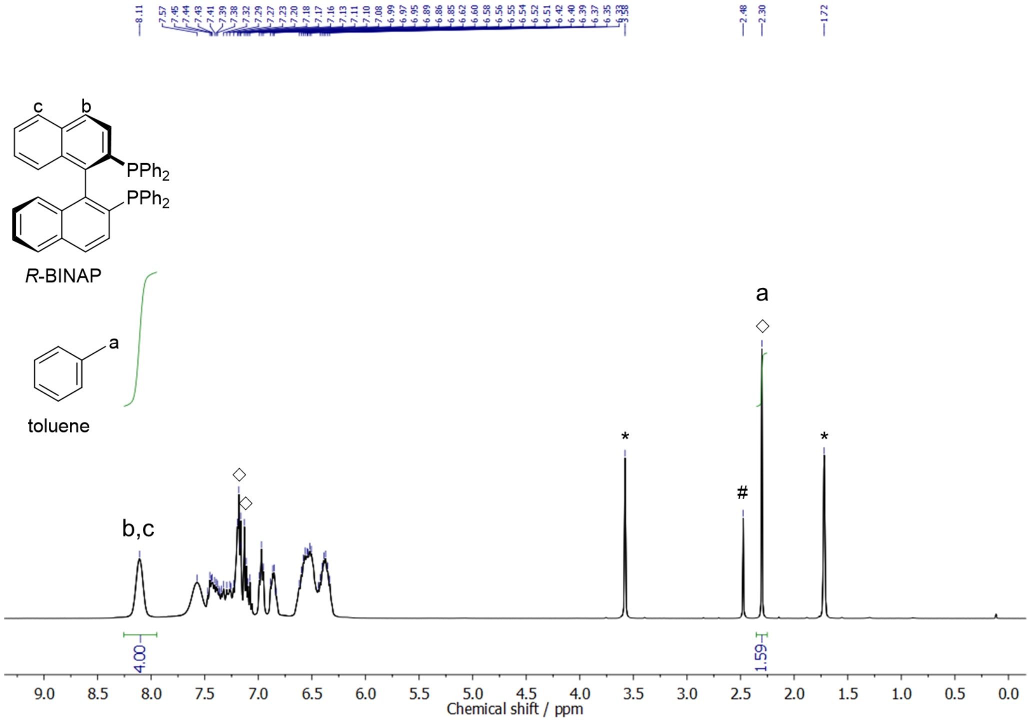

Figure S28. ^{1}H NMR spectrum of **CuRB** dissolved in THF-d_8. Protons of THF, toluene, H_2O were marked with *, ◊ and #, respectively.

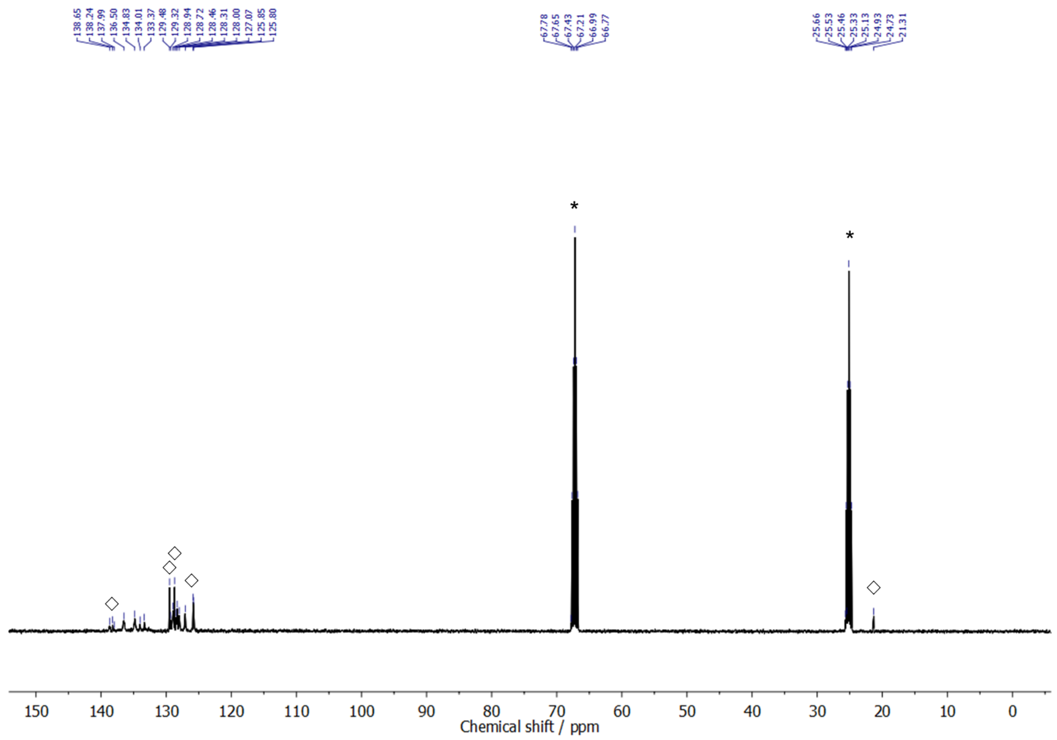

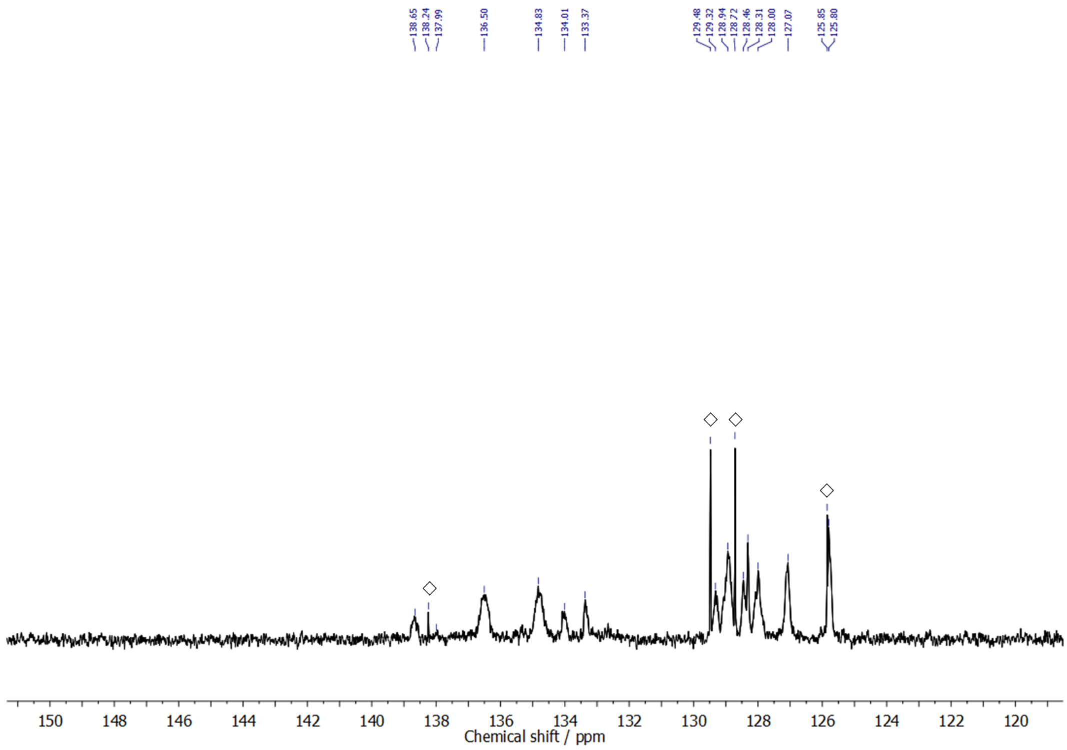

Figure S29. ^{13}C NMR spectrum of **CuRB** dissolved in THF-d_8. Carbons of THF and toluene were marked with * and ◊, respectively.

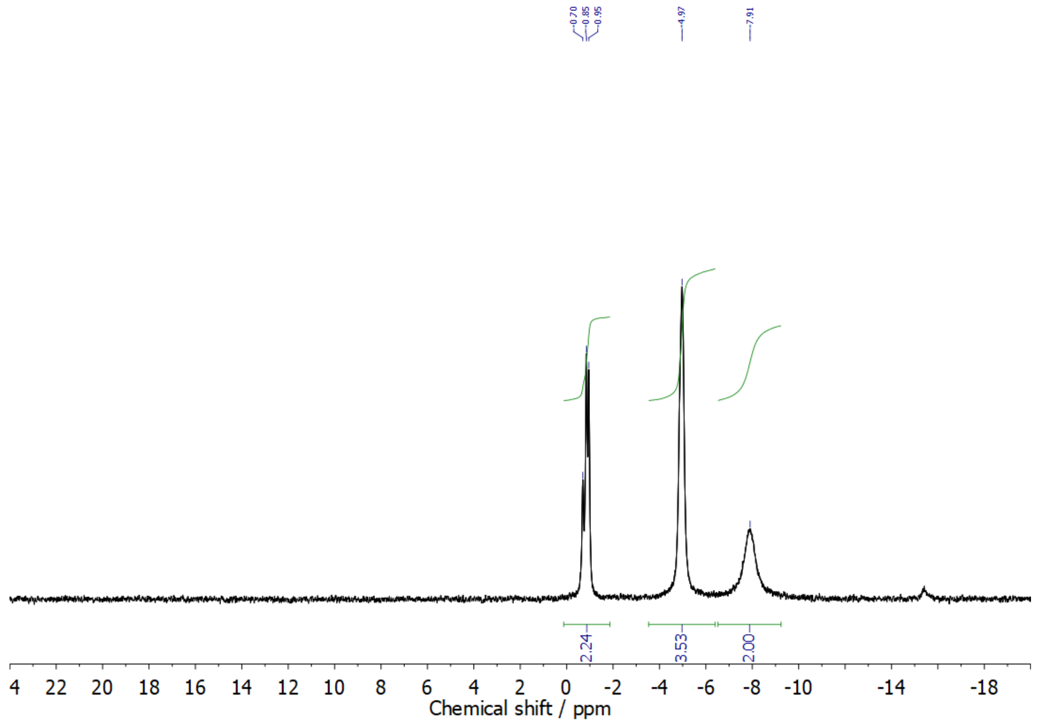

Figure S30. ^{31}P NMR spectrum of **CuRB** dissolved in THF-d_8.

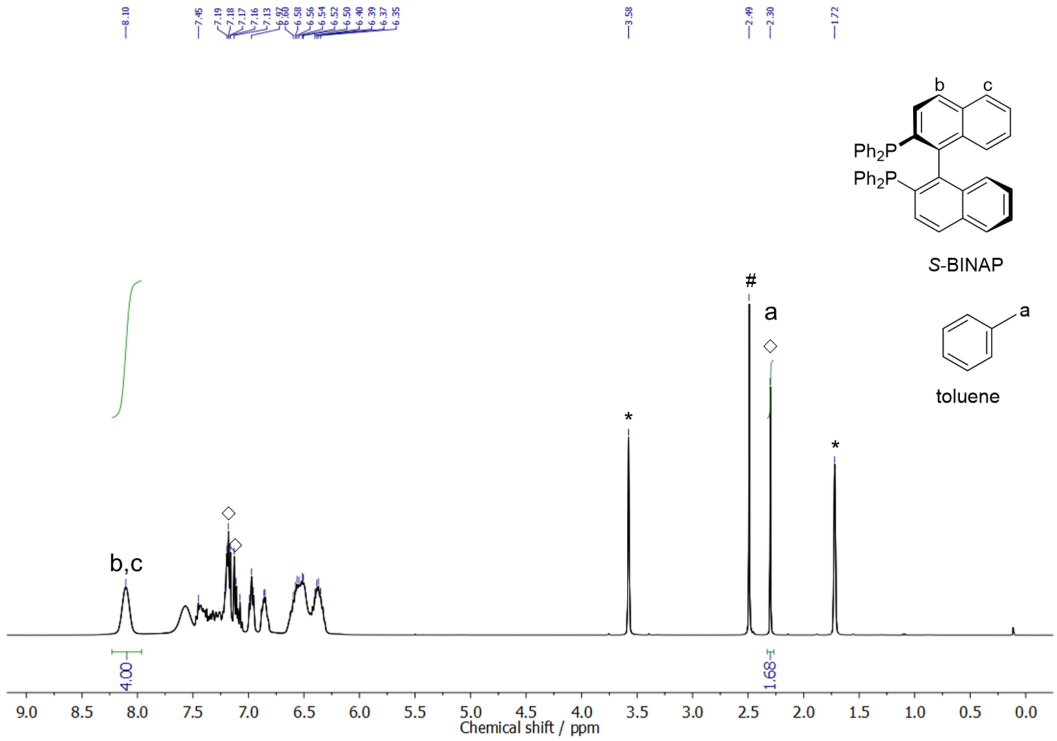

Figure S31. ^{1}H NMR spectrum of **CuSB** dissolved in THF-d_8. Protons of THF, toluene, H_2O were marked with *, ◊ and #, respectively.

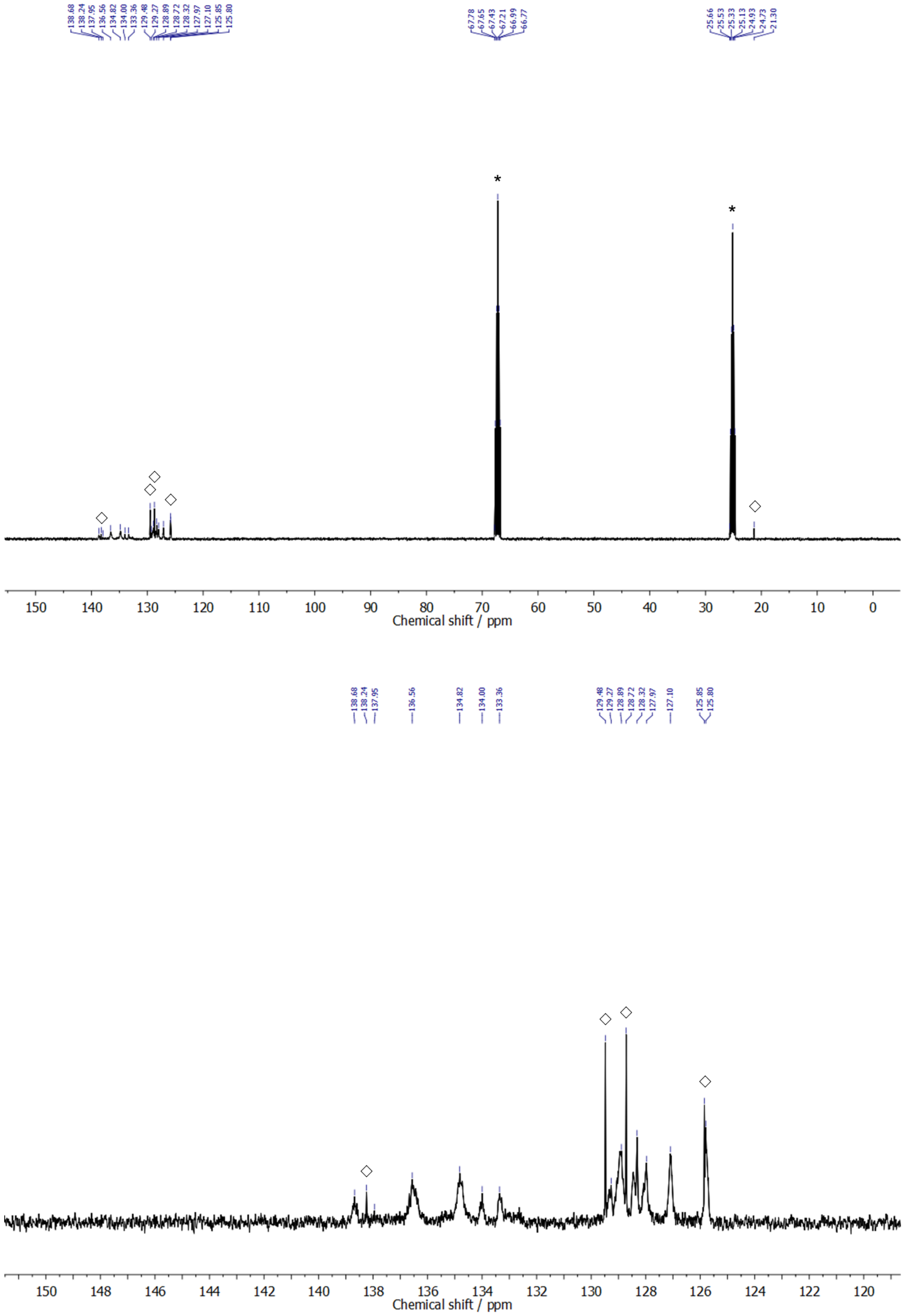

Figure S32. ^{13}C NMR spectrum of **CuSB** dissolved in THF-d_8. Carbons of THF and toluene were marked with * and ◊, respectively.

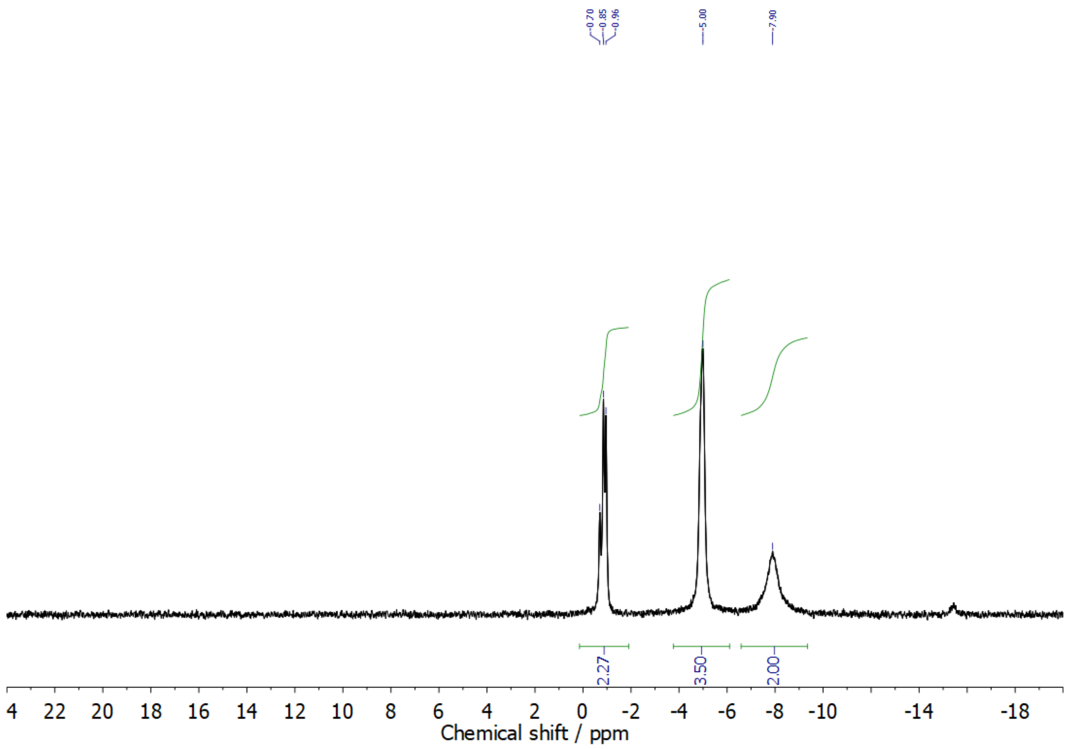

Figure S33. ^{31}P NMR spectrum of **CuSB** dissolved in THF-d_8.

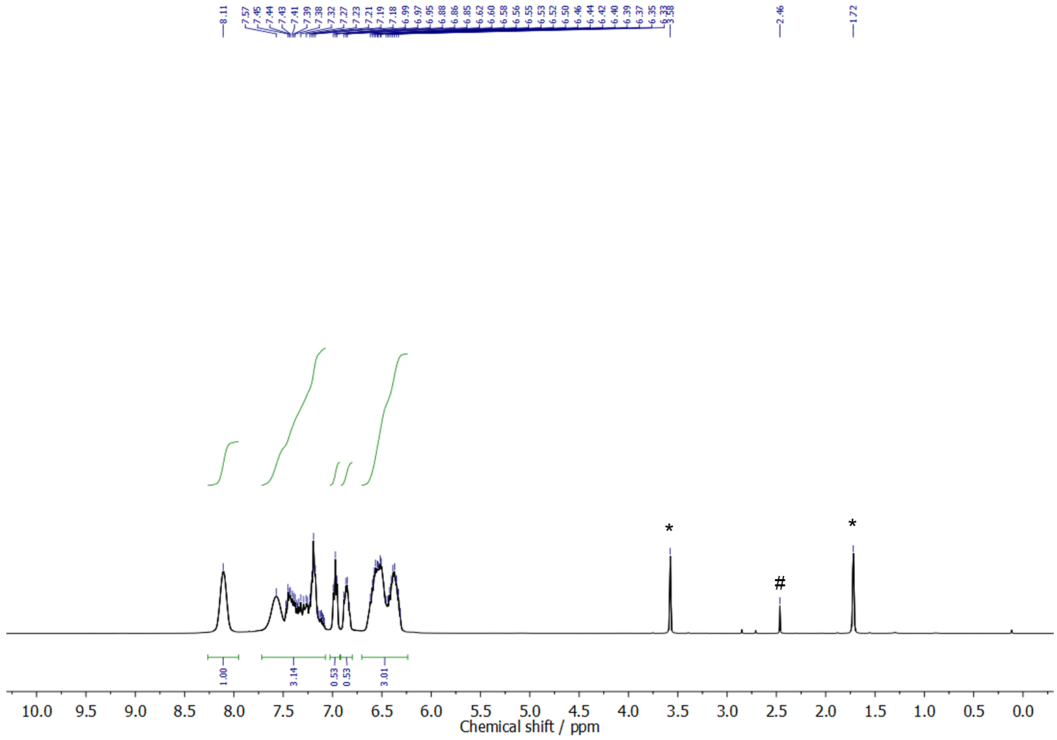

Figure S34. ^{1}H NMR spectrum of ***ds*CuRB** dissolved in THF-d_8. Protons of THF and H_2O were marked with * and #, respectively.

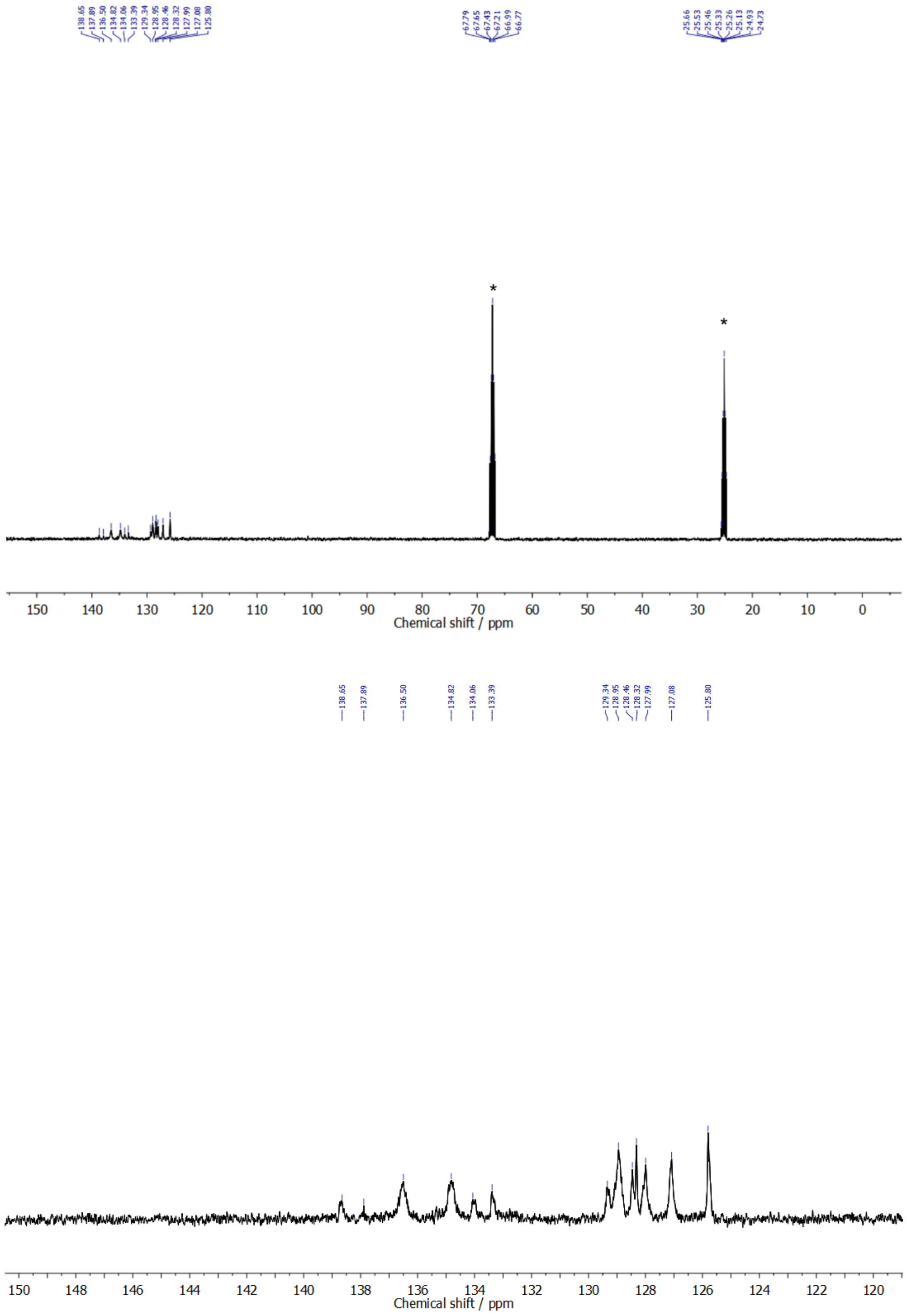

Figure S35. ^{13}C NMR spectrum of ***ds*CuRB** dissolved in THF-d_8. Carbons of THF were marked with *.

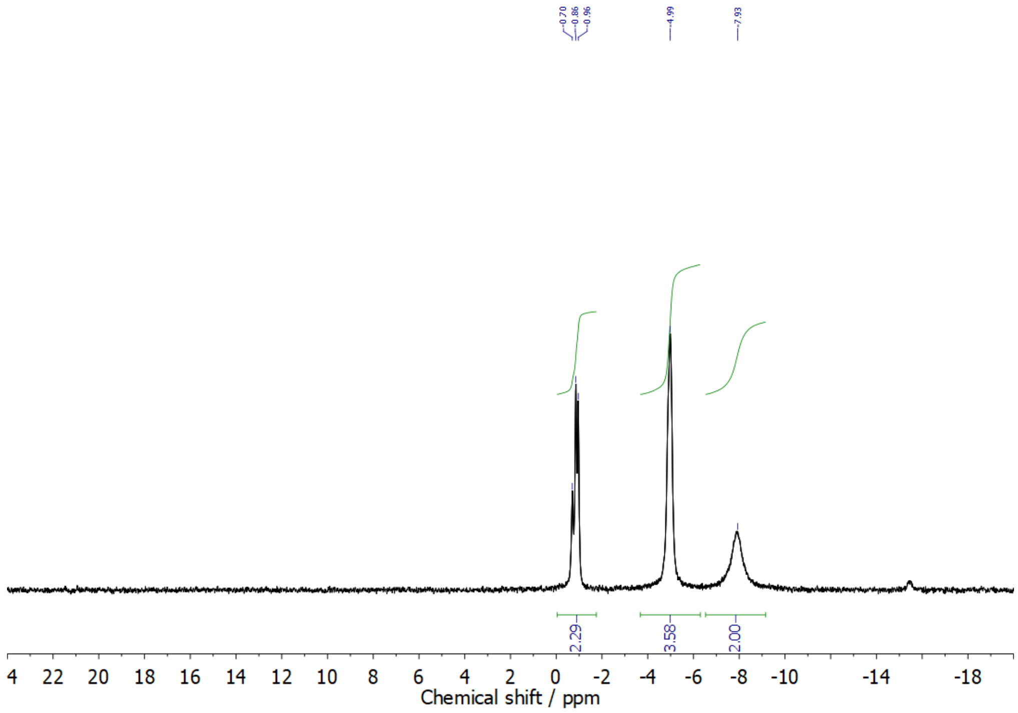

Figure S36. ^{31}P NMR spectrum of ***ds*CuRB** dissolved in THF-d_8.

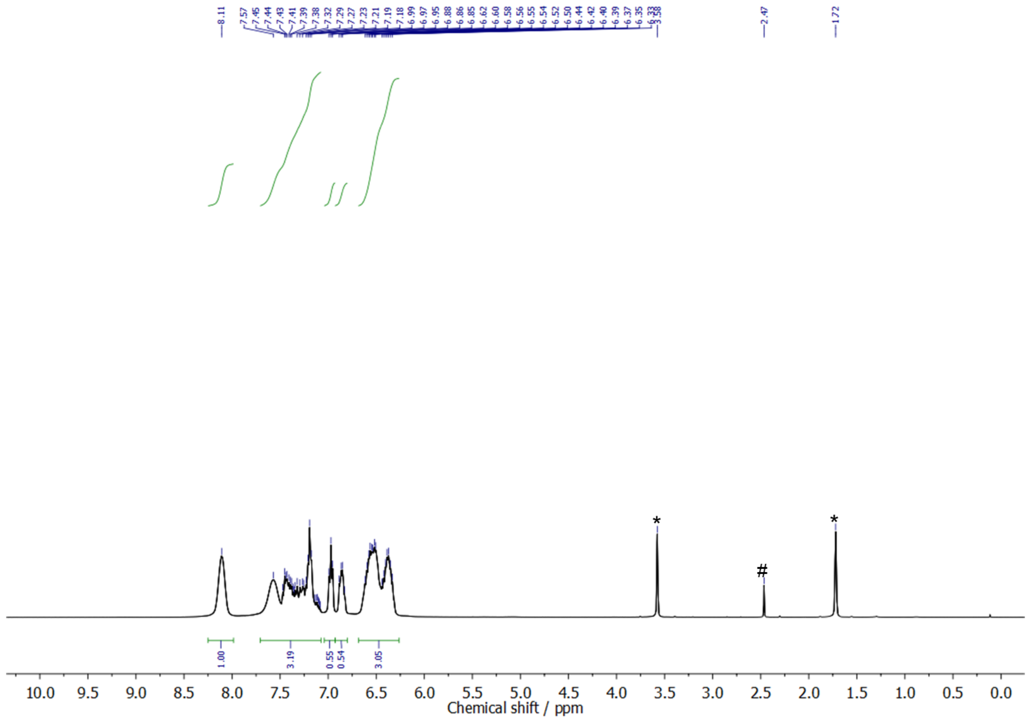

Figure S37. ^{1}H NMR spectrum of ***ds*CuSB** dissolved in THF-d_8. Protons of THF and H_2O were marked with * and #, respectively.

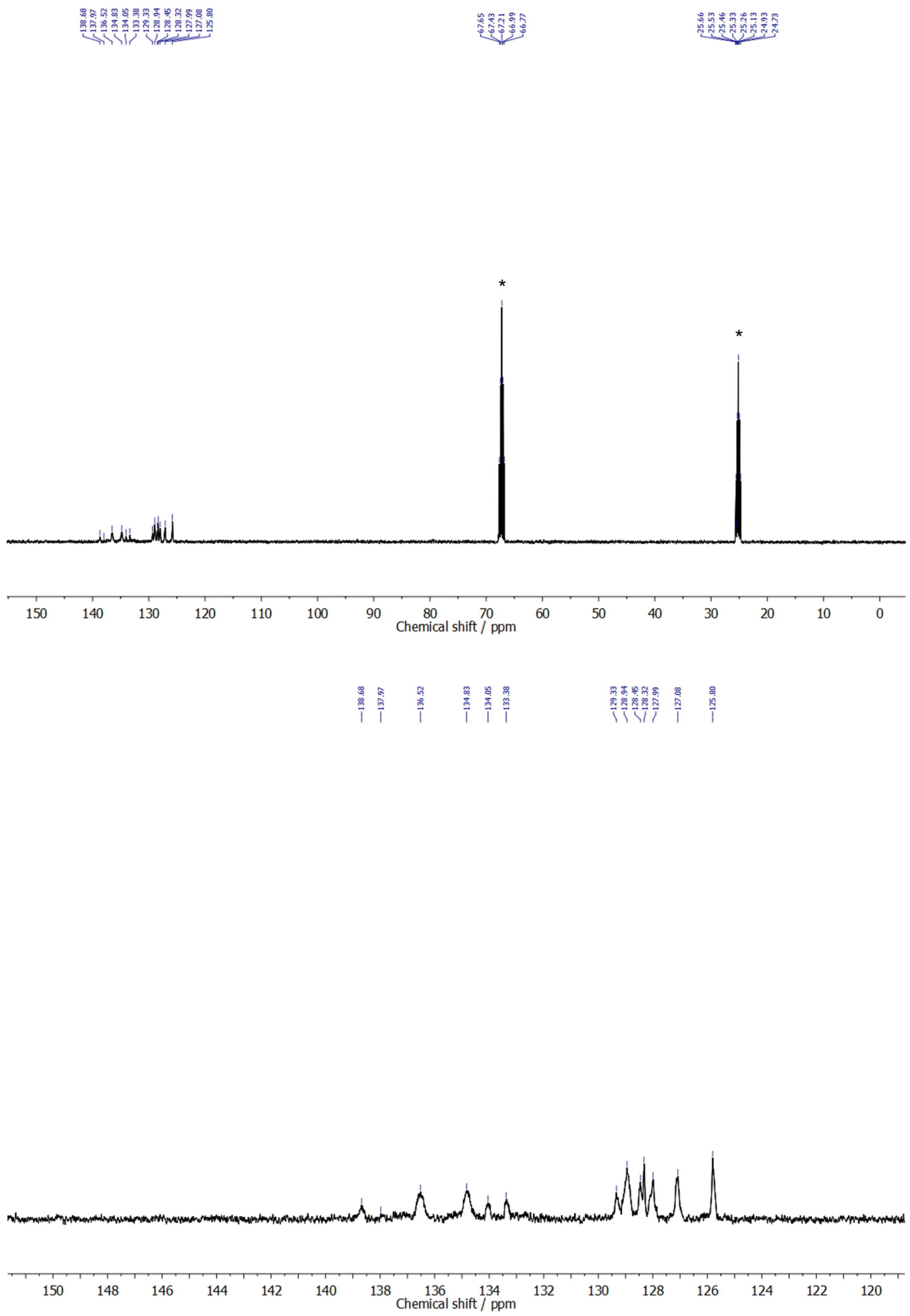

Figure S38. ^{13}C NMR spectrum of ***ds*CuSB** dissolved in THF-d_8. Carbons of THF were marked with *.

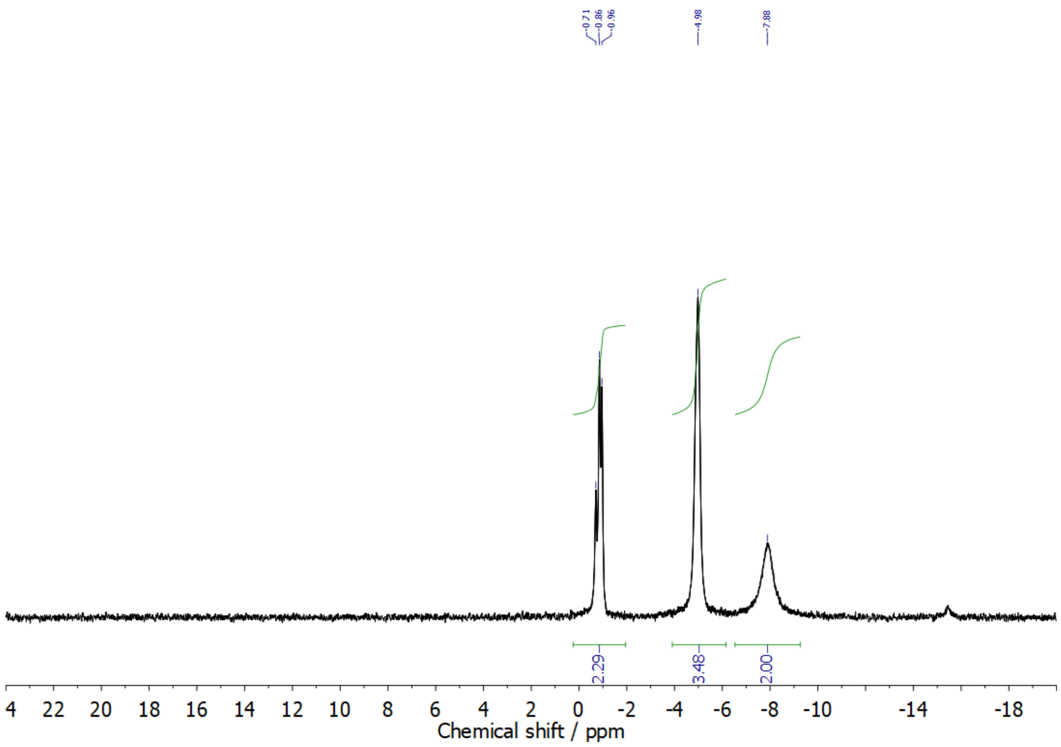

Figure S39. ^{31}P NMR spectrum of ***ds*CuSB** dissolved in THF-d_8.

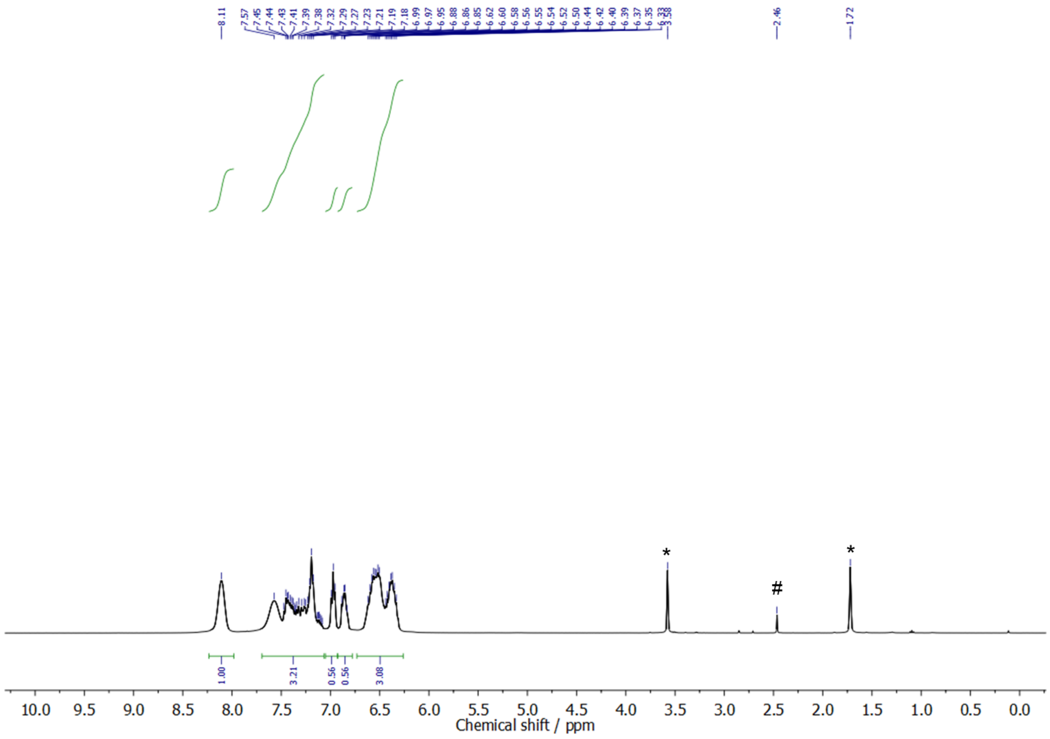

Figure S40. ^{1}H-NMR spectra of ***g*CuRB** dissolved in THF-d_8. Protons of THF and H_2O were marked with * and #, respectively.

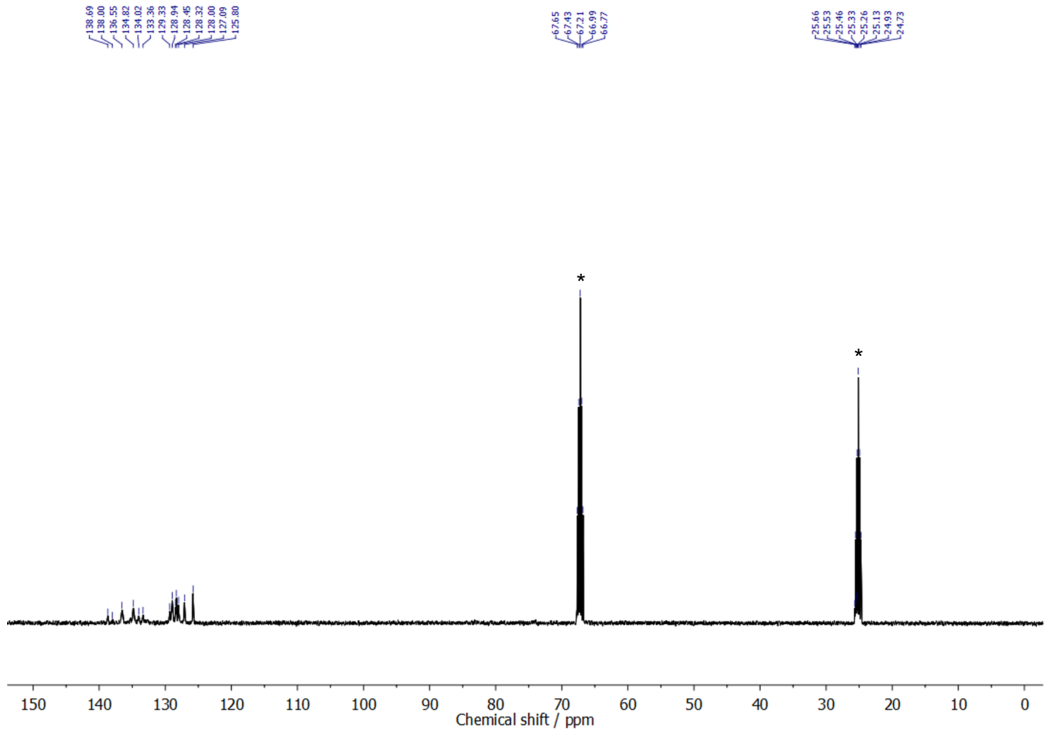

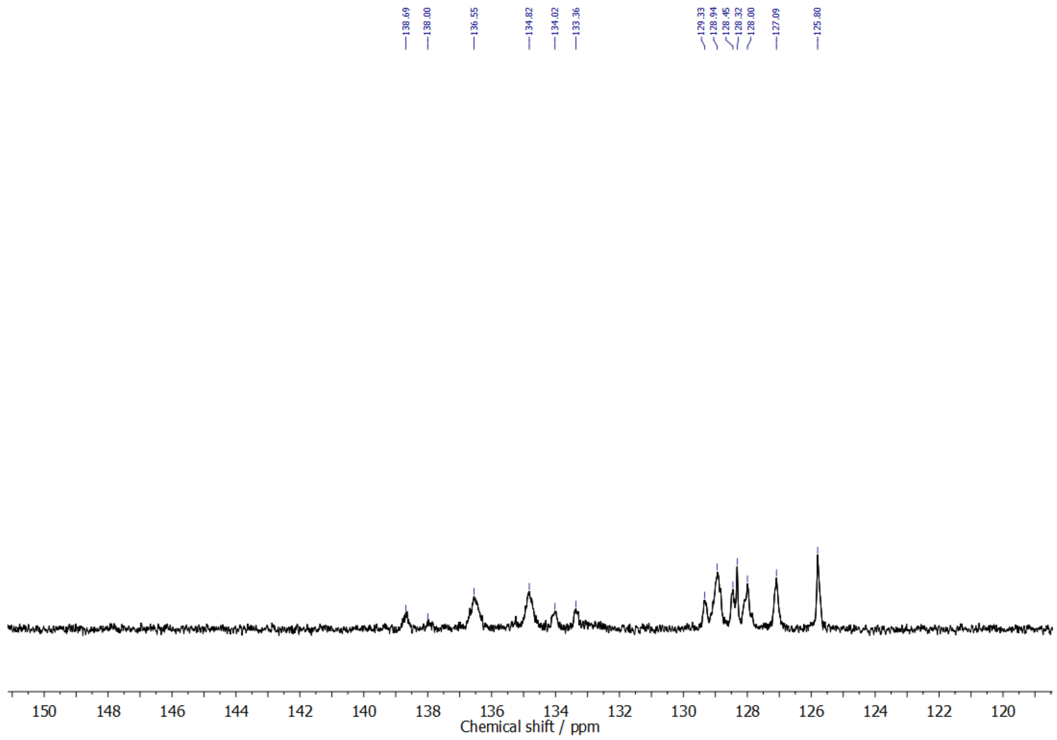

Figure S41. ^{13}C NMR spectrum of ***g*CuRB** dissolved in THF-d_8. Carbons of THF were marked with *.

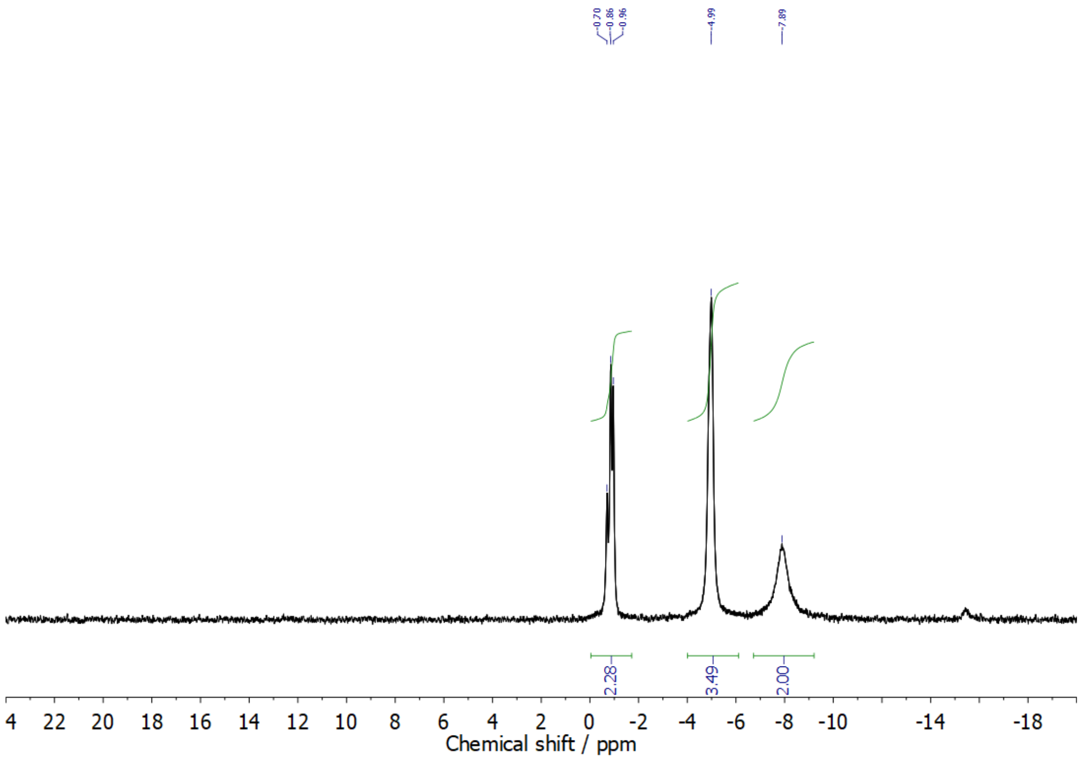

Figure S42. ^{31}P NMR spectrum of ***g*CuRB** dissolved in THF-d_8.

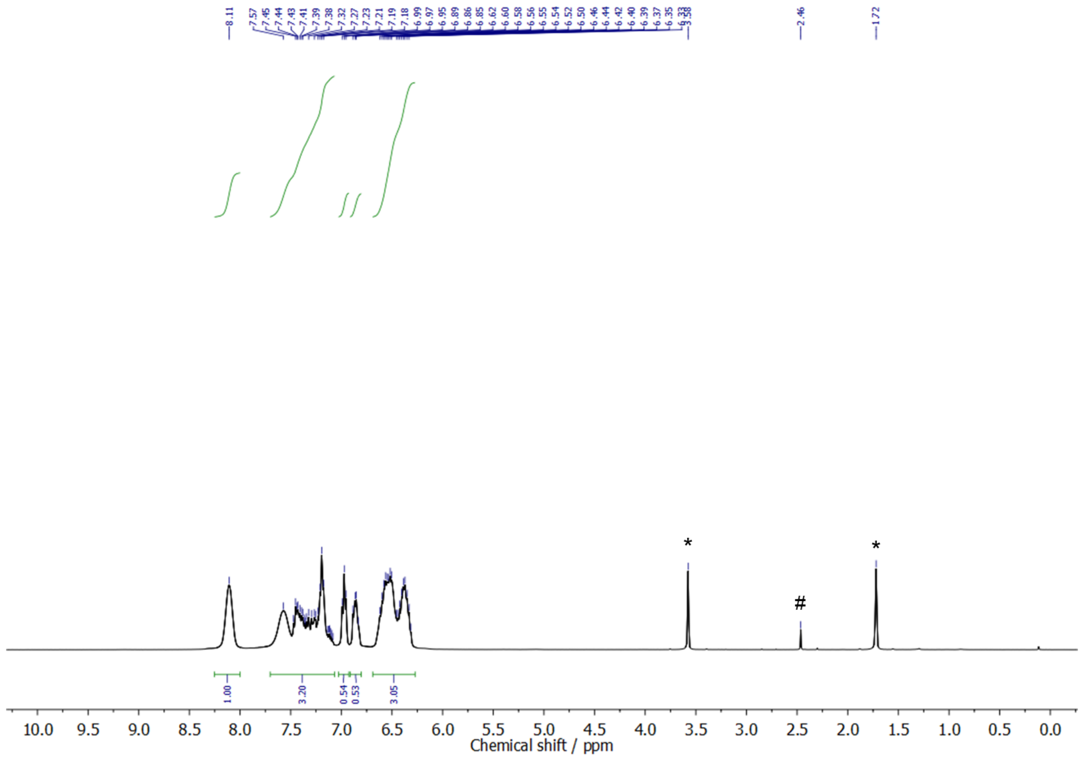

Figure S43. ^{1}H NMR spectrum of ***g*CuSB** dissolved in THF-d_8. Protons of THF and H_2O were marked with * and #, respectively.

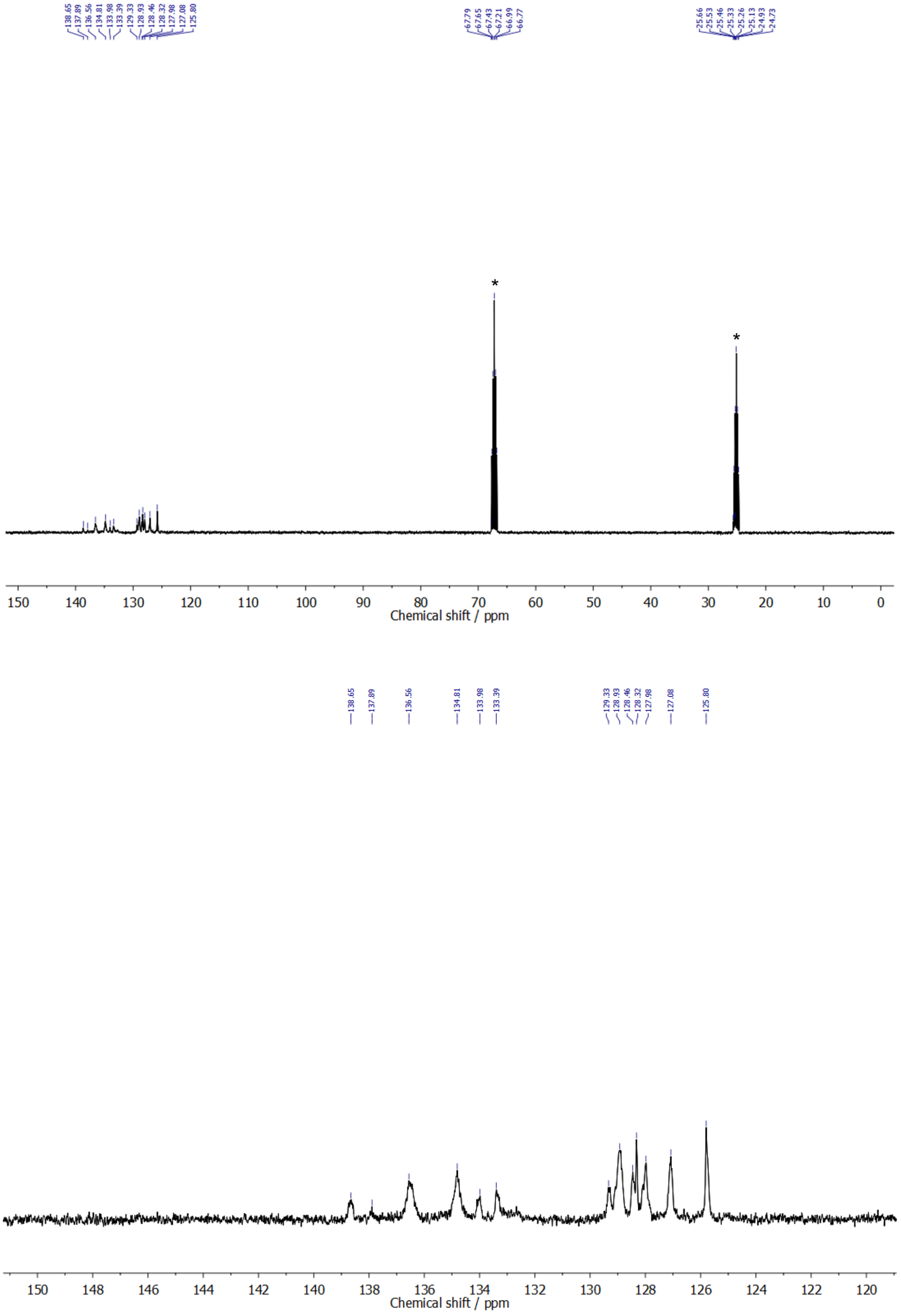

Figure S44. ^{13}C NMR spectrum of ***g*CuSB** dissolved in THF-d_8. Carbons of THF were marked with *.

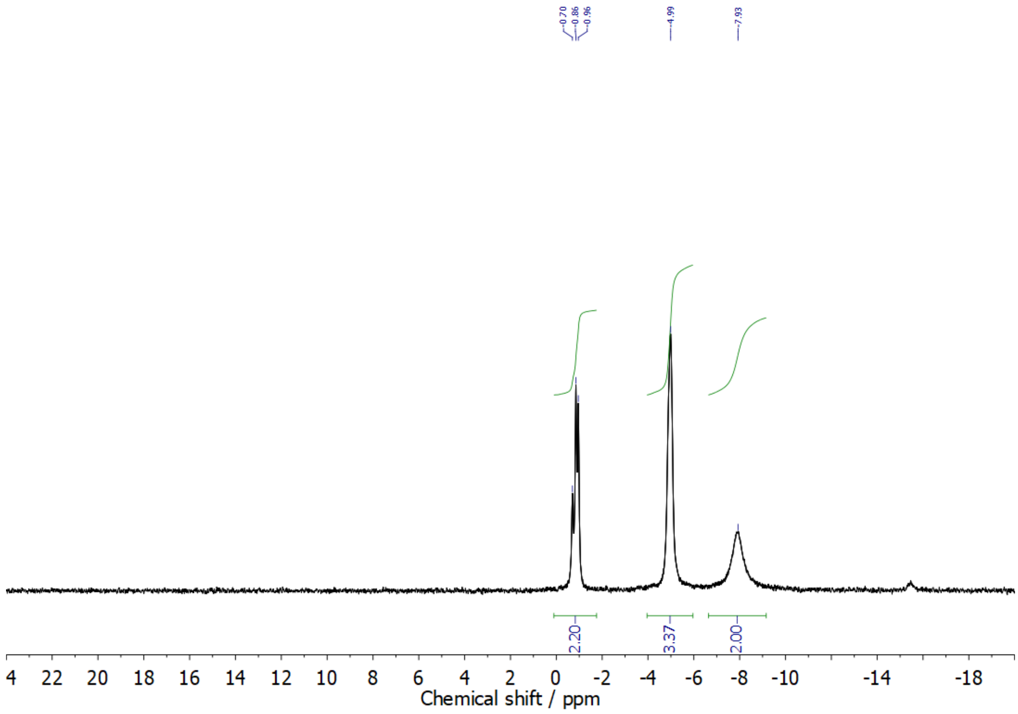

Figure S45. ^{31}P NMR spectrum of ***g*CuSB** dissolved in THF-d_8.

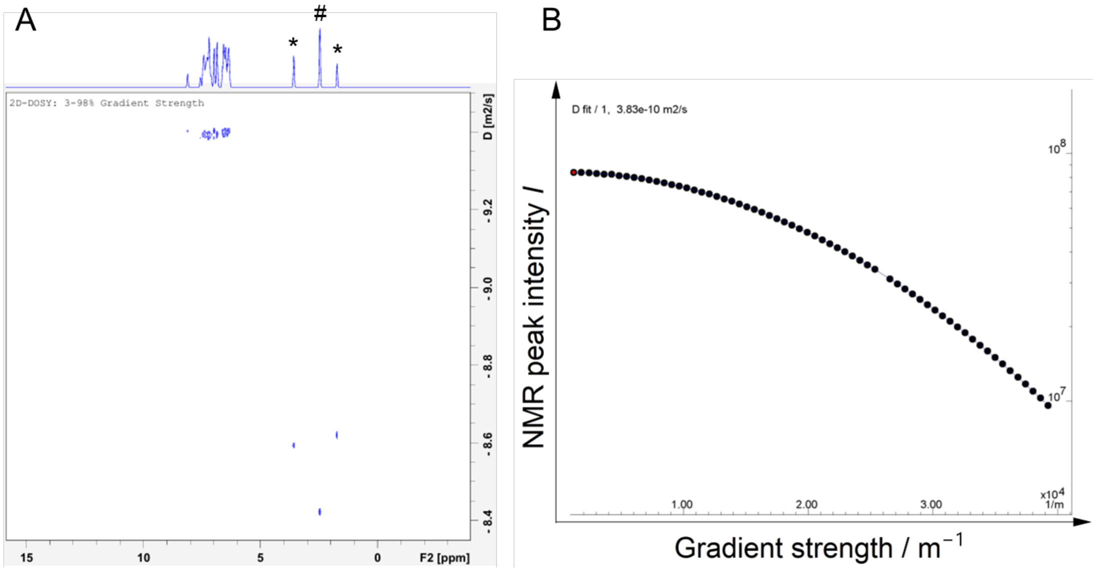

Figure S46. (A) 2D DOSY spectra of ***ds*CuRB** dissolved in THF-d_8. (B) Diffusion coefficient at 8.16 ppm. Residual signals of THF and H_2O were marked with * and #, respectively.

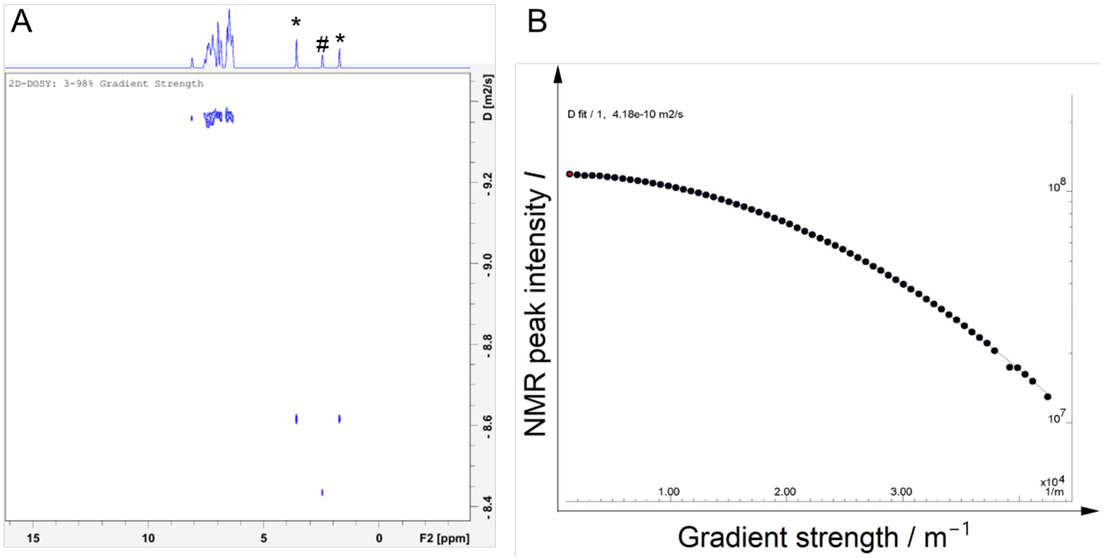

Figure S47. (A) 2D DOSY spectra of ***ds*CuSB** dissolved in THF-d_8. (B) Diffusion coefficient at 8.16 ppm. Residual signals of THF and H_2O were marked with * and #, respectively.

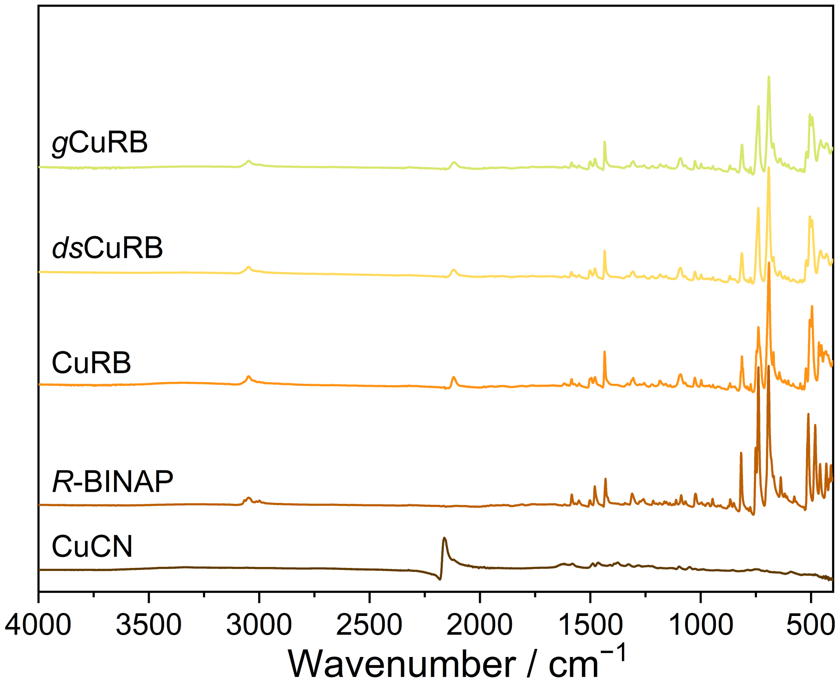

Figure S48. FT-IR spectra of CuCN, *R*-BINAP, **CuRB**, ***ds*CuRB** and ***g*CuRB**.

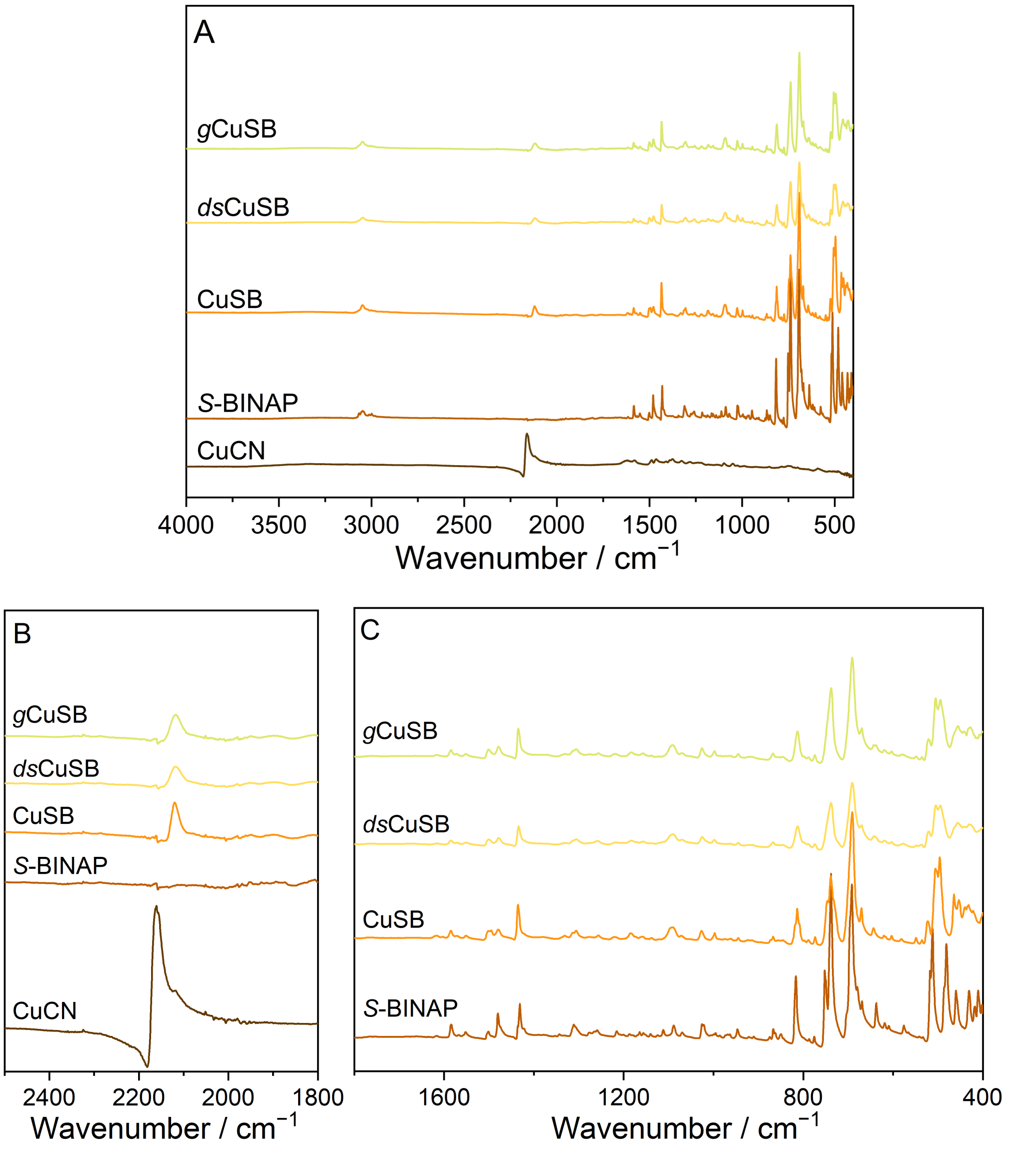

Figure S49. (A) FT-IR spectra of CuCN, *S*-BINAP, **CuSB**, ***ds*CuSB** and ***g*CuSB**. (B) Zoomed in the region of 2500 to 1800 cm^{-1}. (C) Zoomed in the region of 1800 to 400 cm^{-1}.

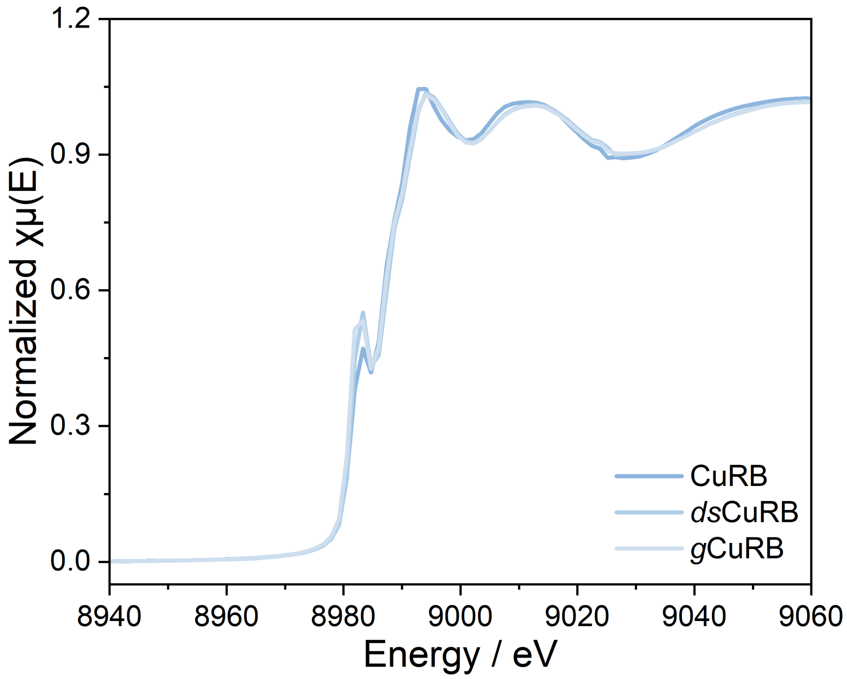

Figure S50. XANES of **CuRB**, ***ds*CuRB** and ***g*CuRB**.

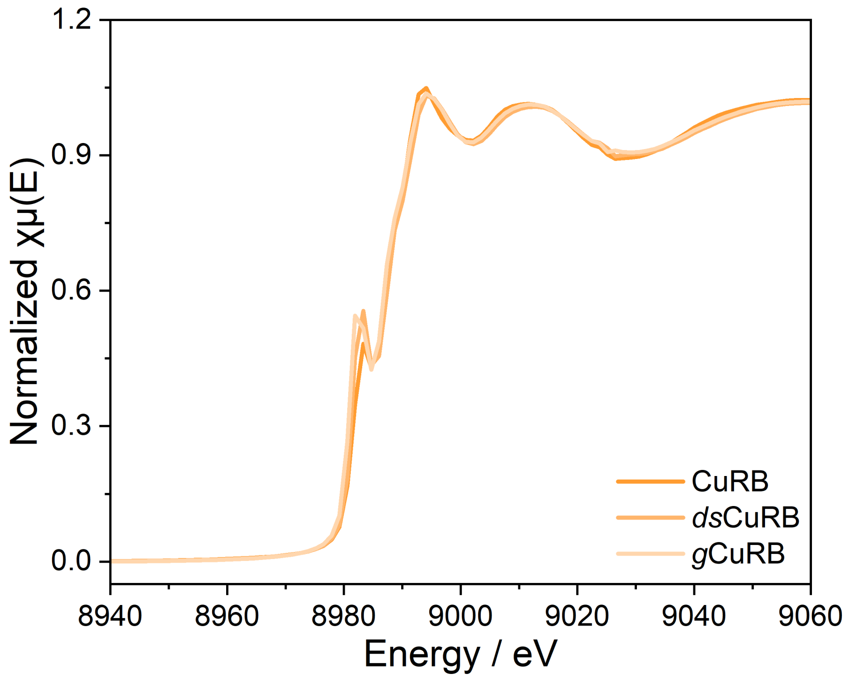

Figure S51. XANES of **CuSB**, ***ds*CuSB** and ***g*CuSB**.

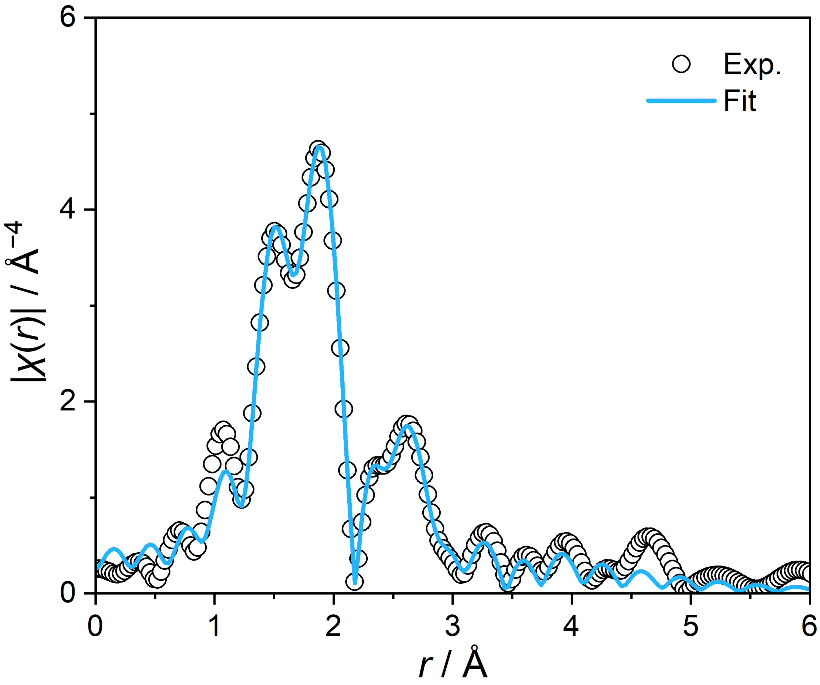

Figure S52. EXAFS of **CuRB**.

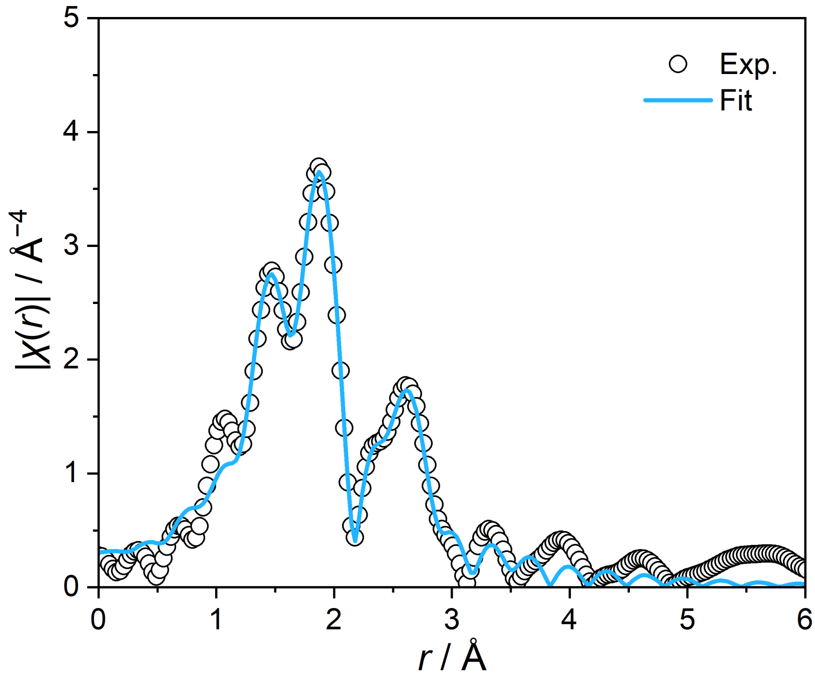

Figure S53. EXAFS of ***ds*CuRB**.

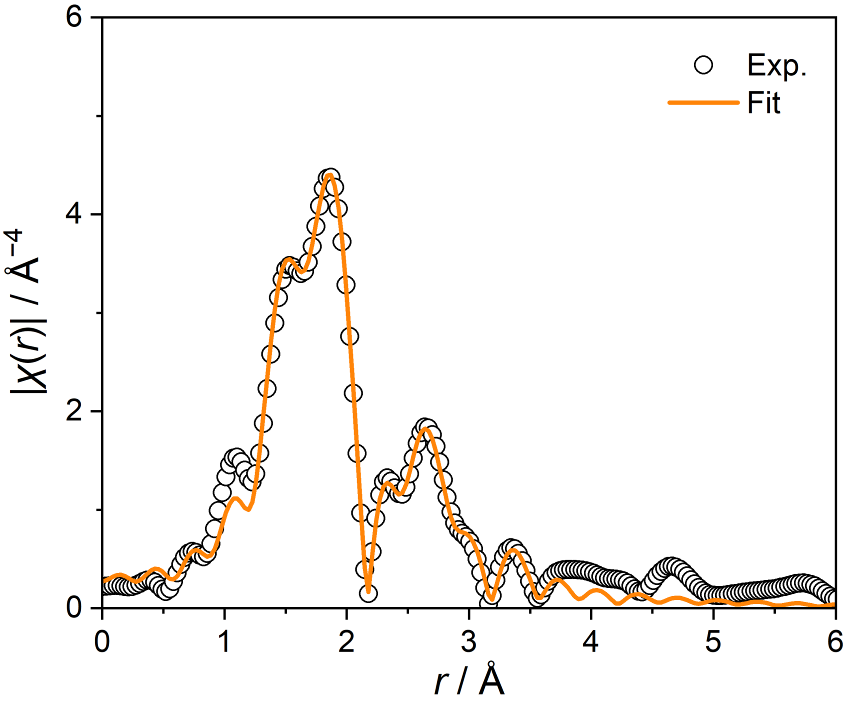

Figure S54. EXAFS of **CuSB**.

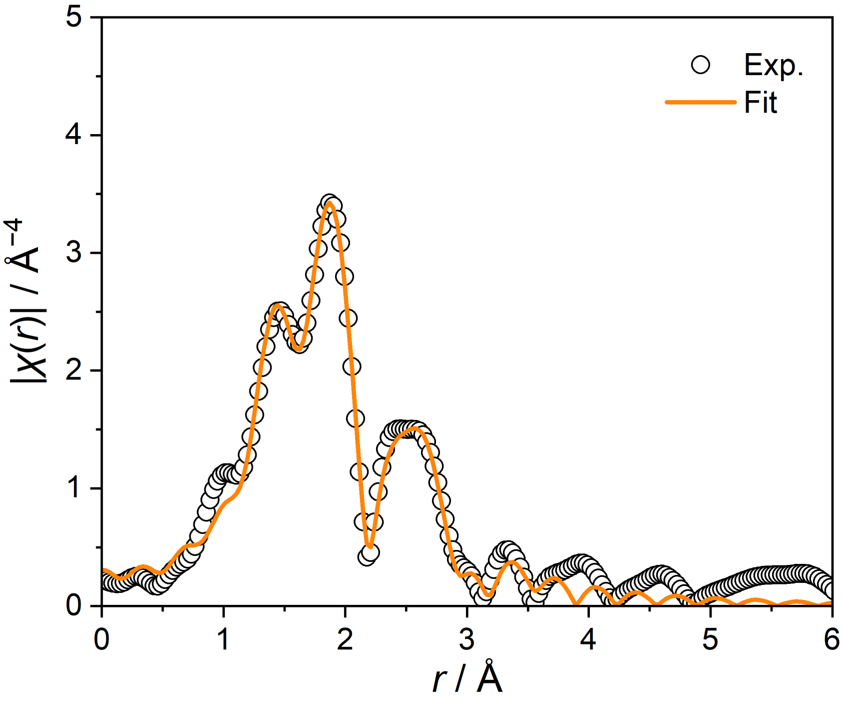

Figure S55. EXAFS of ***ds*CuSB**.

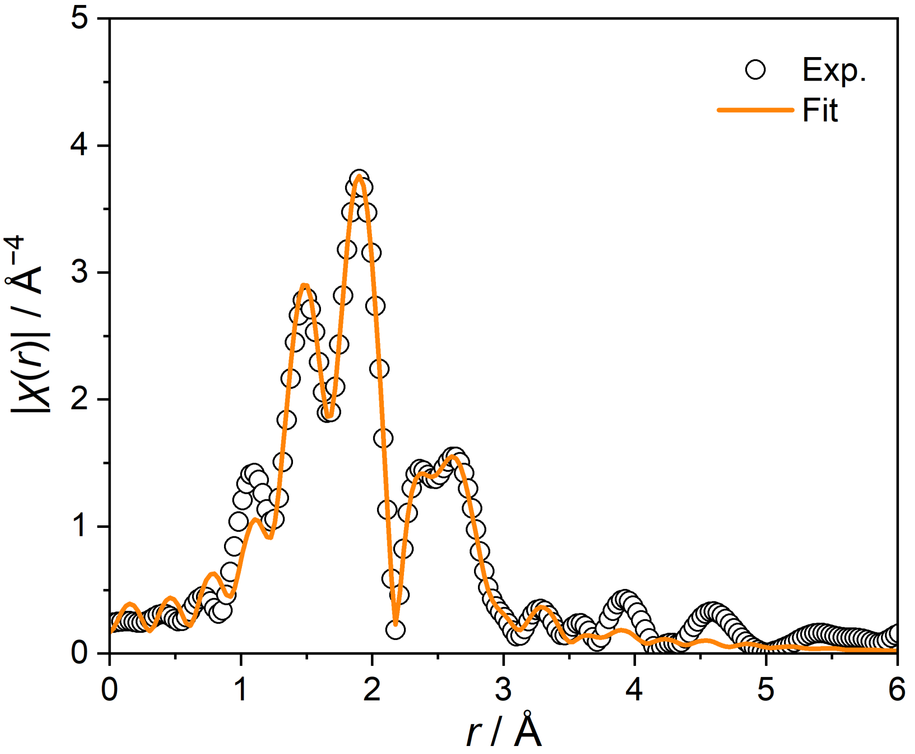

Figure S56. EXAFS of ***g*****CuSB**.

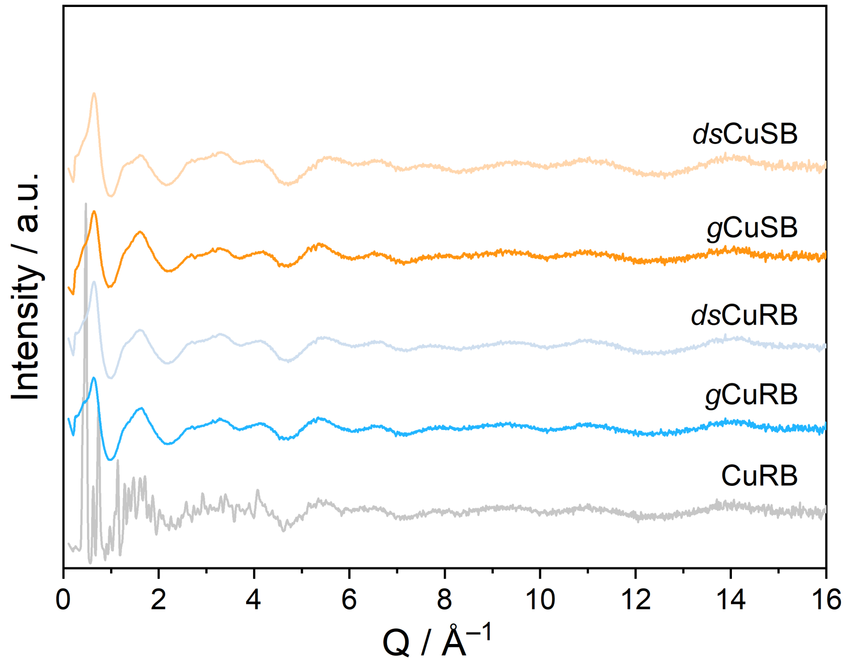

Figure S57. X-ray total scattering data in the form $S(Q)$ of crystalline and glassy samples in this study.

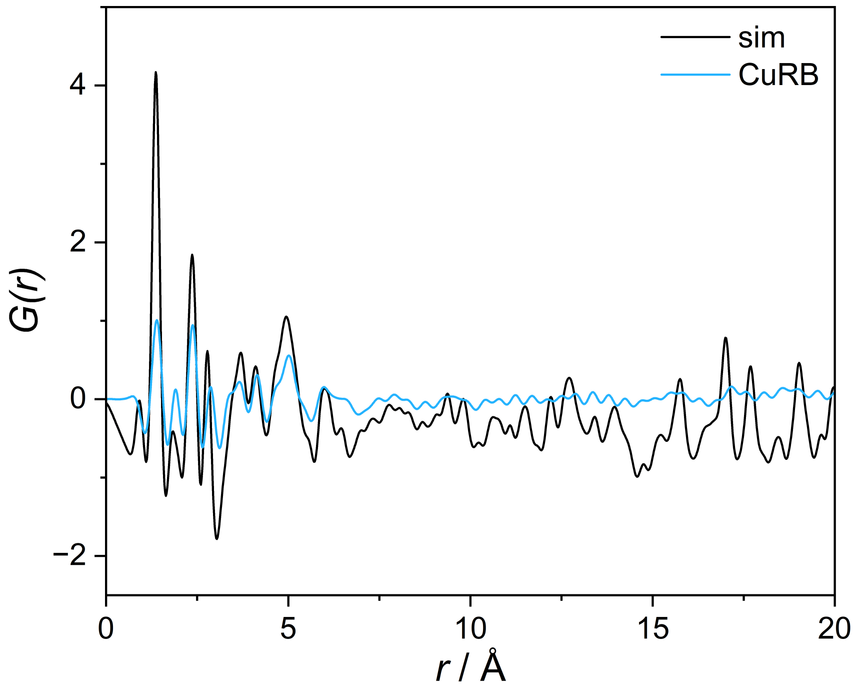

Figure S58. Simulated and experimental PDF profile of **CuRB** in the form of $G(r)$. Simulation was done by the script xPDFsim.[19]

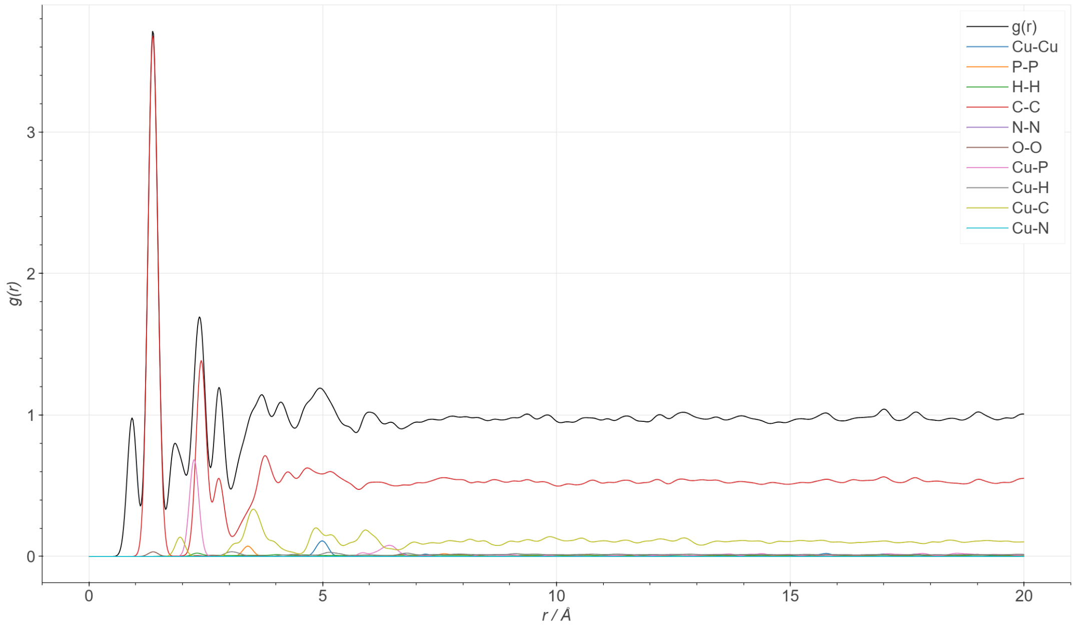

Figure S59. Simulated PDF profile of **CuRB** in the form of $g(r)$. Simulation was done by the script xPDFsim.[19]

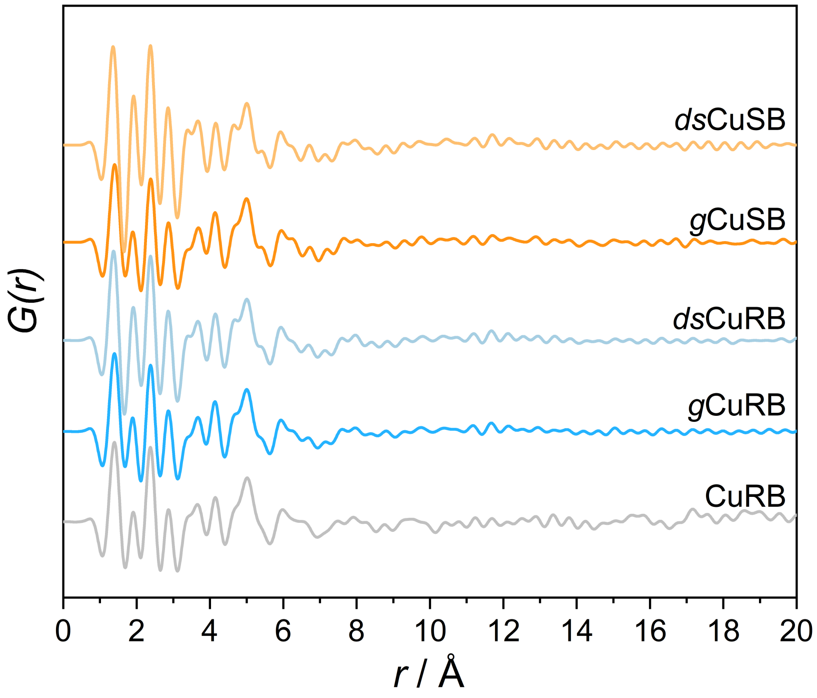

Figure S60. PDF profiles of of crystalline and glassy samples in this study.

$S_0 \rightarrow S_1$	$S_0 \rightarrow S_2$
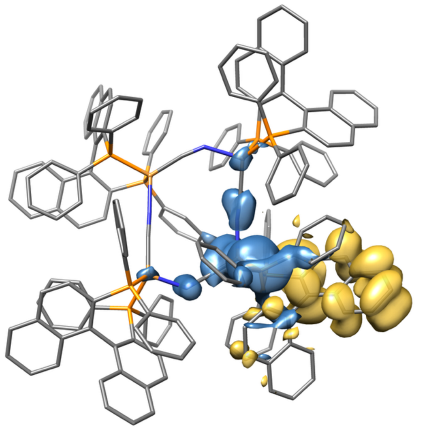	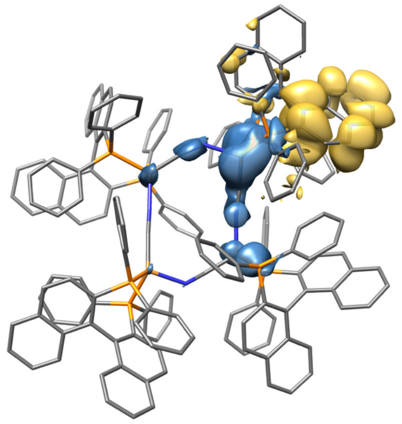
$S_0 \rightarrow S_{10}$	
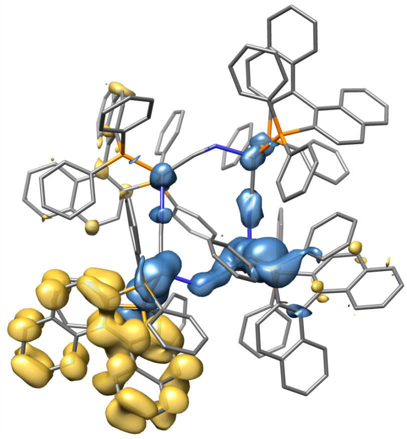	

$S_0 \rightarrow T_1$	$S_0 \rightarrow T_2$
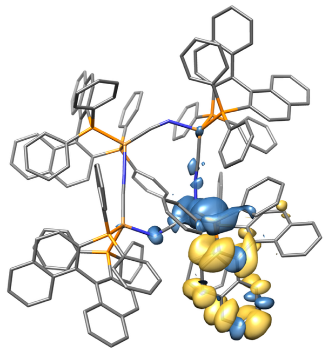	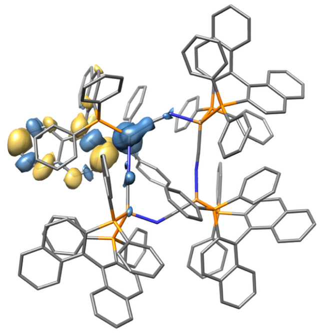
$S_0 \rightarrow T_4$	$S_0 \rightarrow T_5$
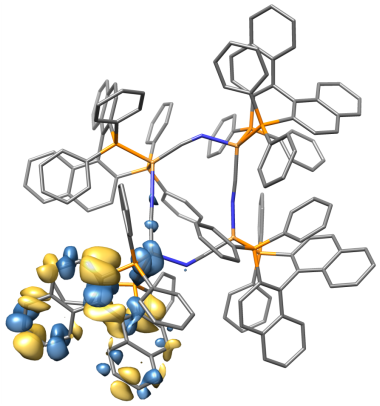	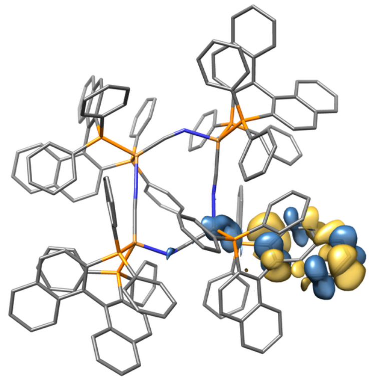
$S_0 \rightarrow T_{10}$	
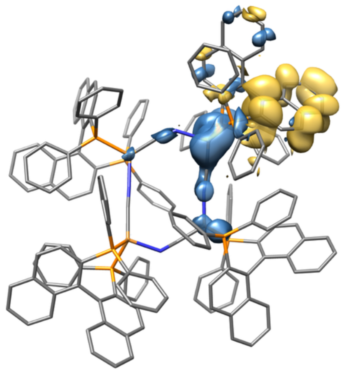	

Figure S61. Comparison of TD-DFT calculated transition density differences for vertical excitation from the DFT optimized ground state S_0 to selected excited singlet states S_n and excited triplet states T_n for **CuRB**. Loss of electron density shown in blue, gain in yellow.

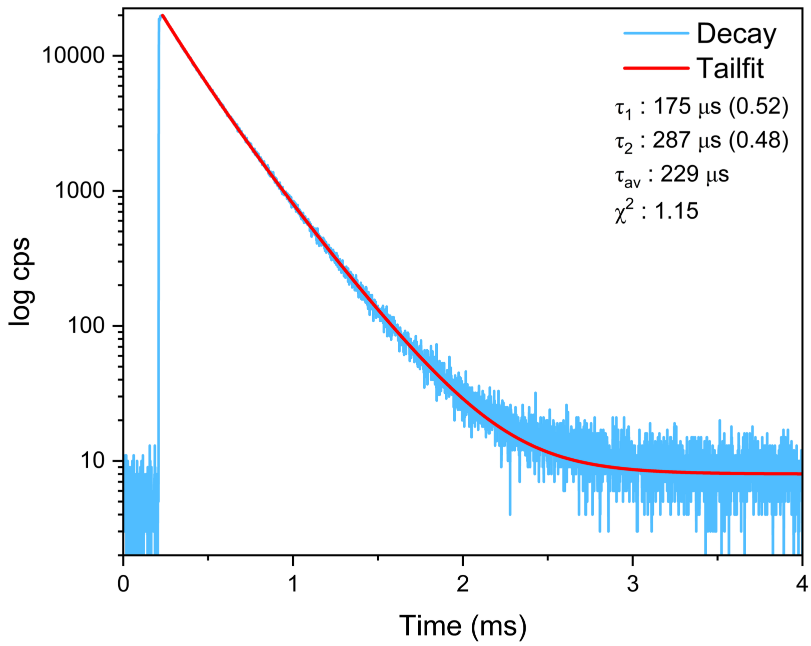

Figure S62. Emission lifetime decay of **CuRB** in THF at 545 nm and 25 °C.

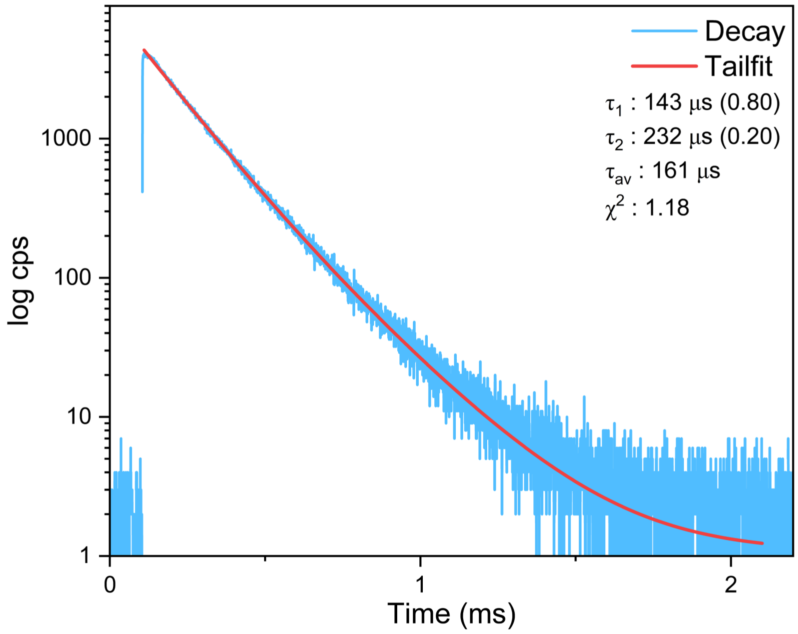

Figure S63. Emission lifetime decay of **CuRB** in toluene at 545 nm and 25 °C.

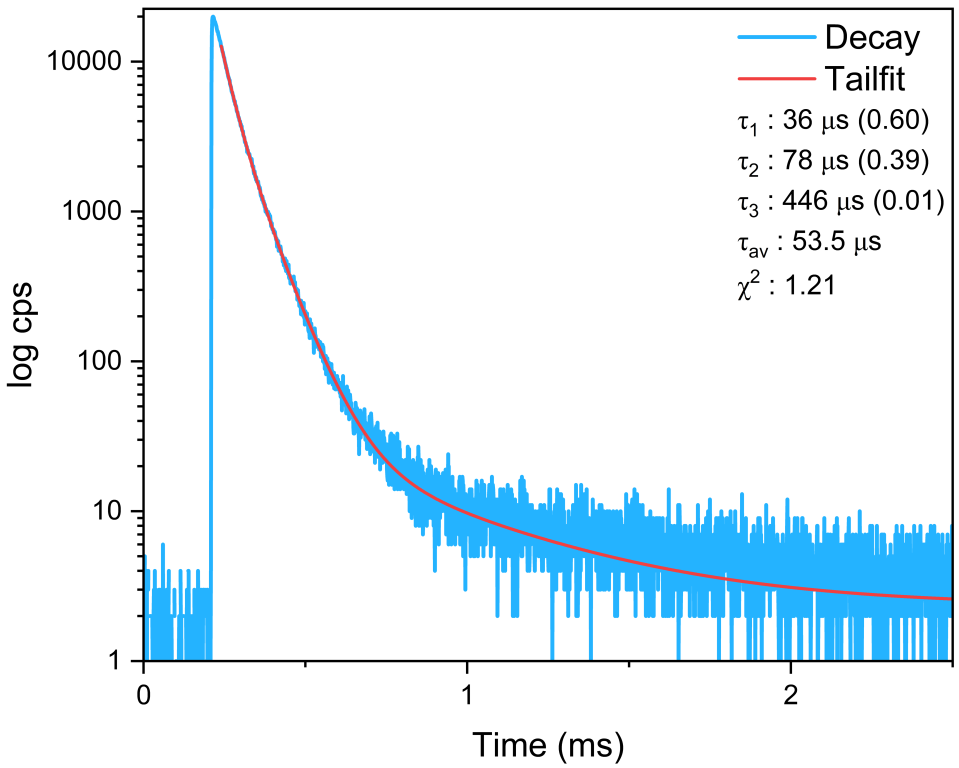

Figure S64. Emission lifetime decay of microcrystalline **CuRB** at 545 nm and 25 °C.

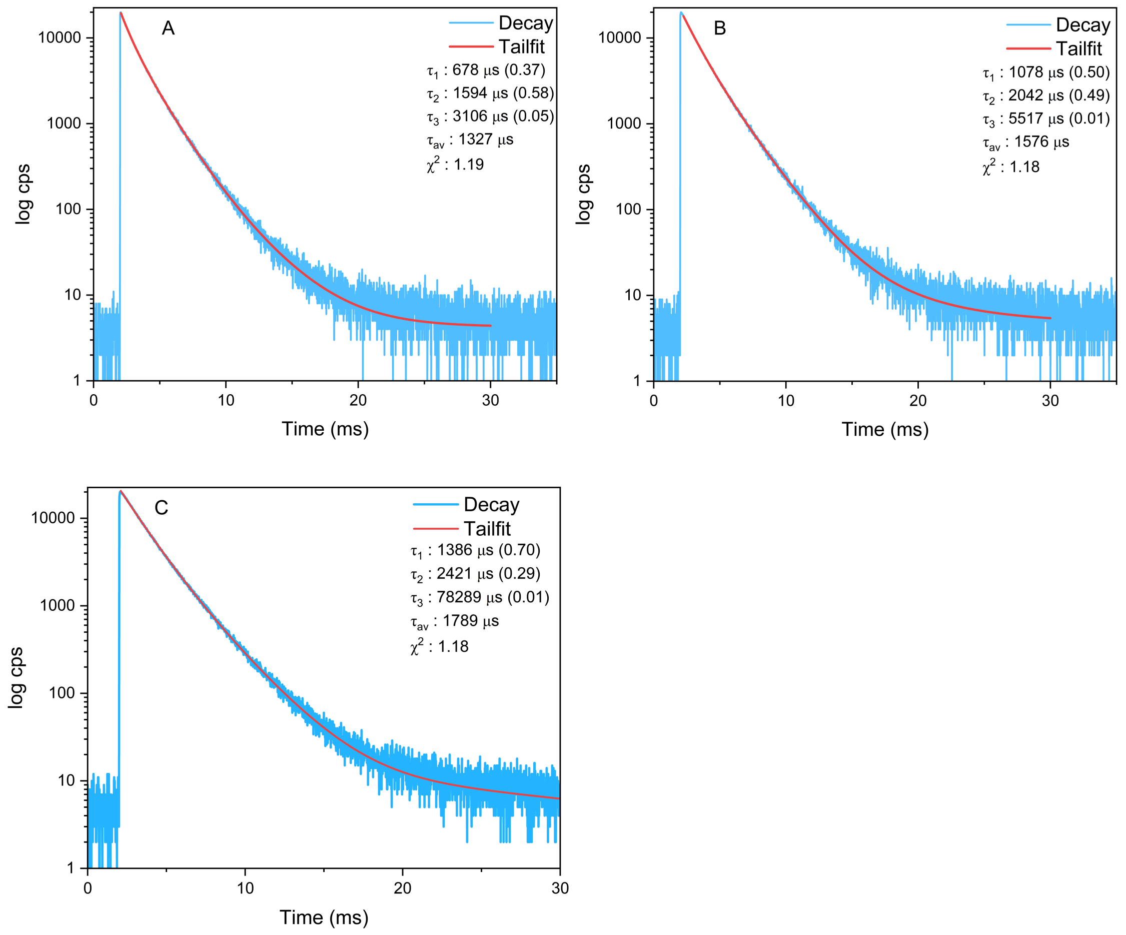

Figure S65. Emission lifetime decay of microcrystalline **CuRB** at (A) 545, (B) 577 and (C) 645 nm at –196 °C.

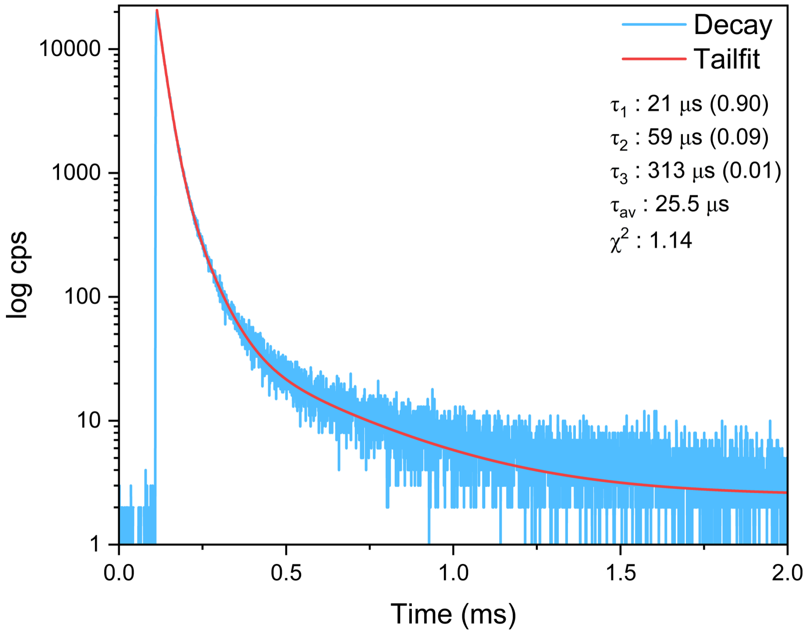

Figure S66. Emission lifetime decay of ***g*CuRB** at 590 nm and 25 °C.

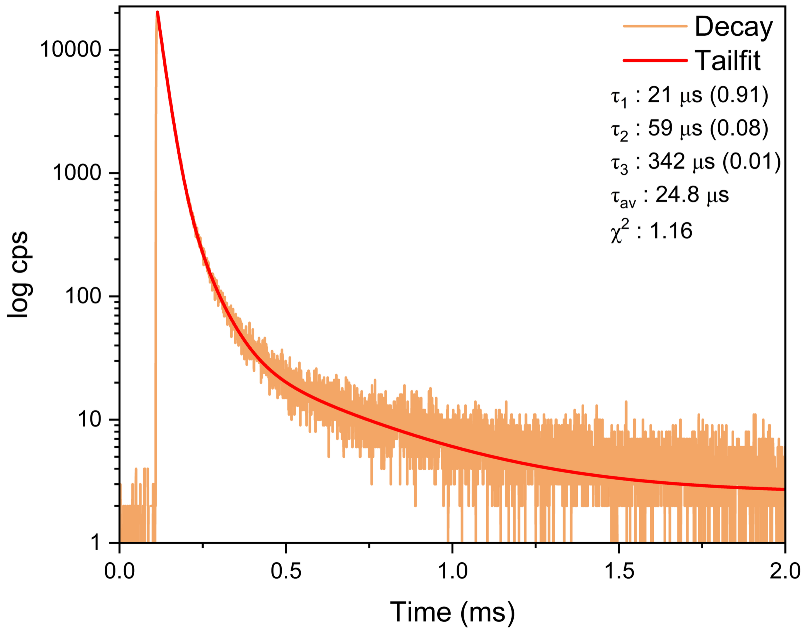

Figure S67. Emission lifetime decay of ***g*CuSB** at 591 nm and 25 °C.

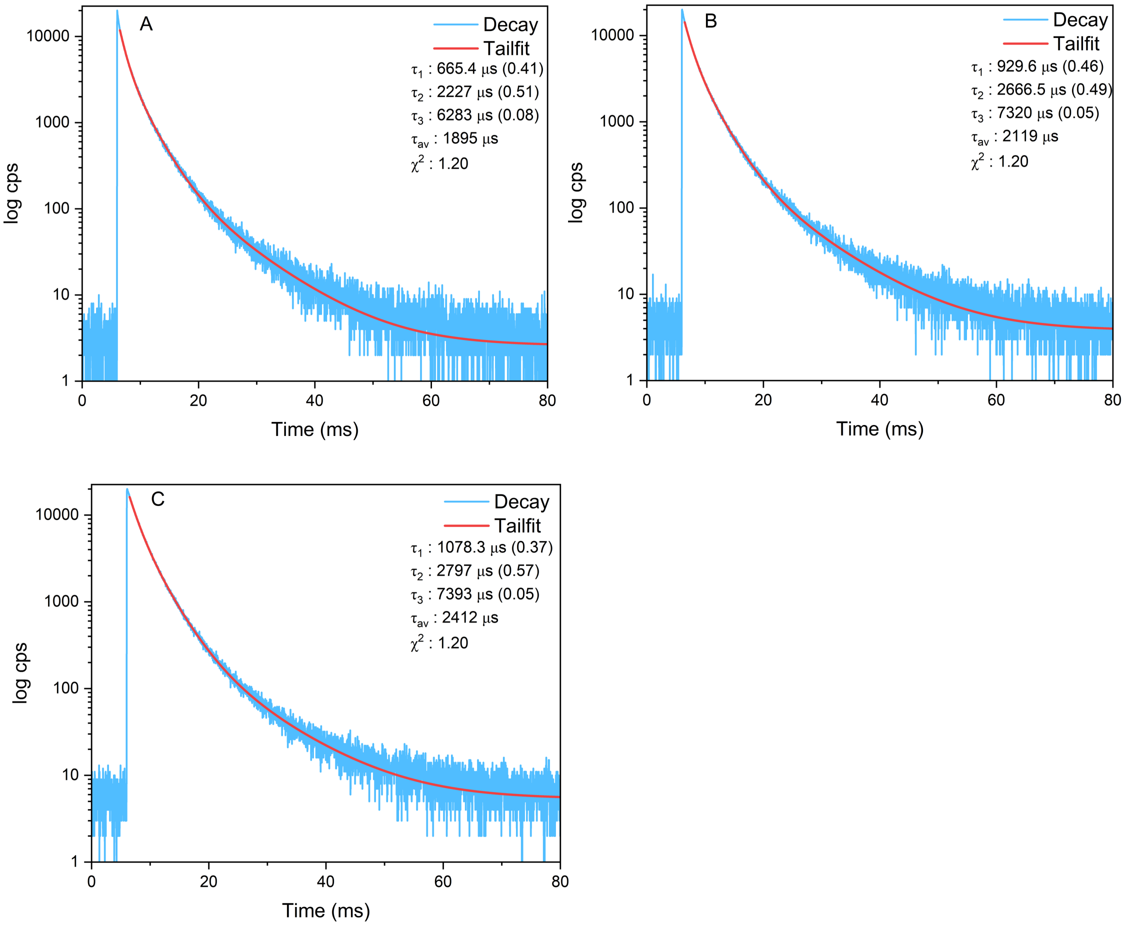

Figure S68. Emission lifetime decay of ***g*CuRB** at (A) 548 (lifetime recorded at 520 nm), (B) 578 and (C) 630 nm (lifetime recorded at 690 nm) at –196 °C.

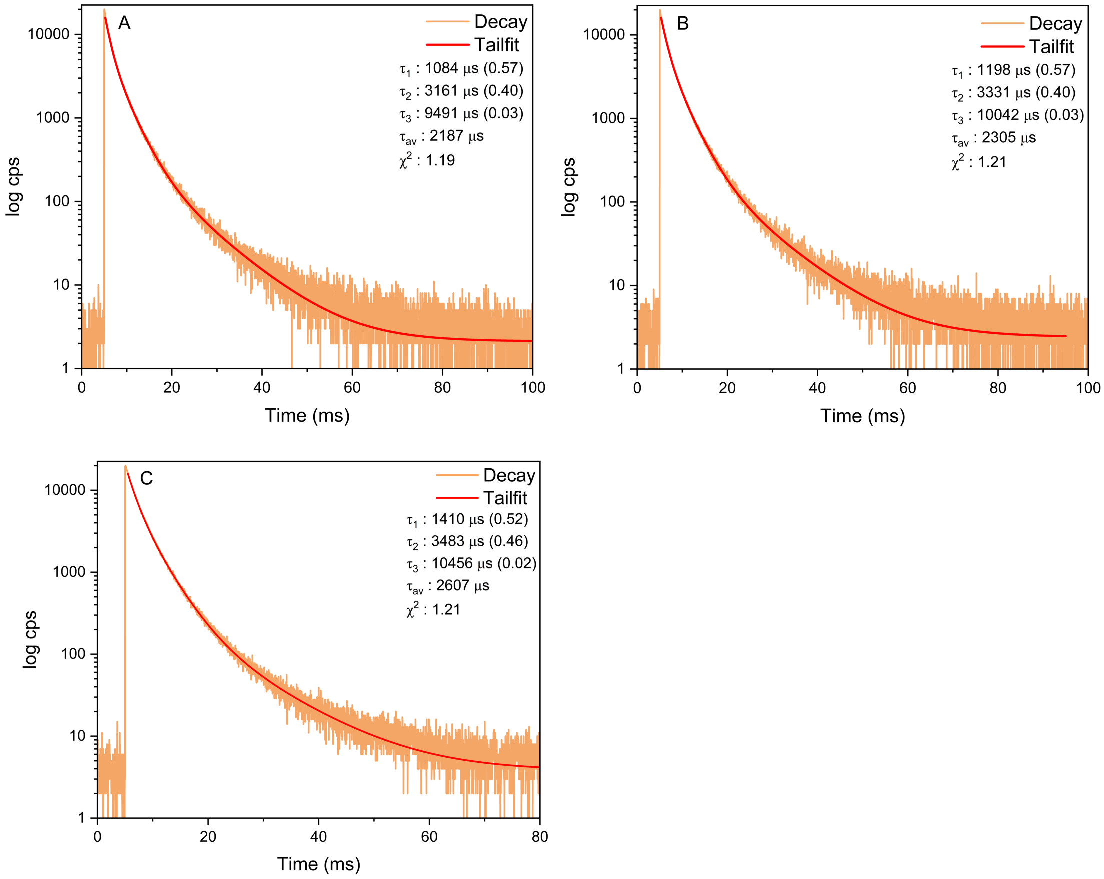

Figure S69. Emission lifetime decay of ***g*CuSB** at (A) 547 (lifetime recorded at 520 nm), (B) 580 and (C) 630 nm (lifetime recorded at 690 nm) at –196 °C.

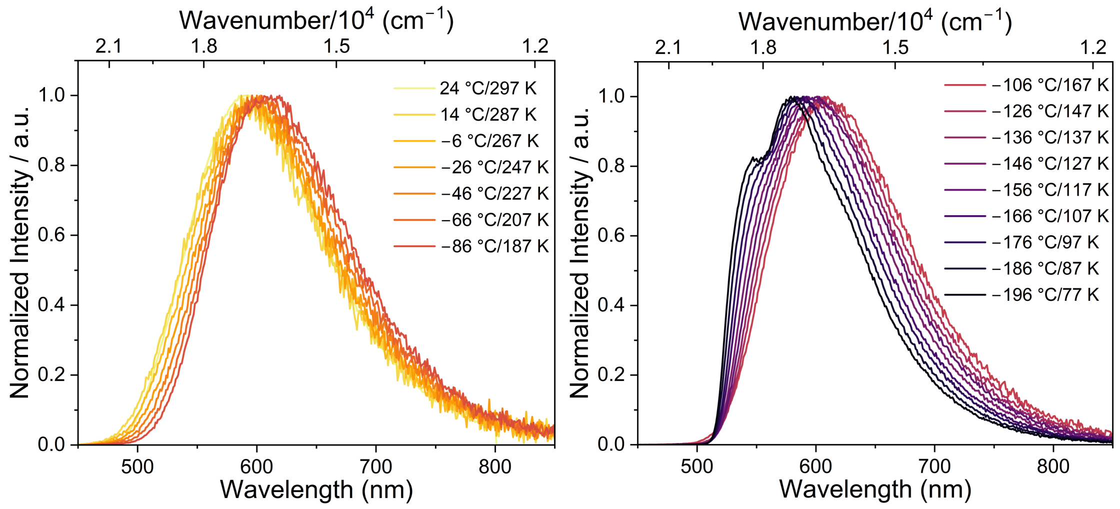

Figure S70. Variable temperature emission spectra of ***g*CuRB** from 24 °C (297 K) to –196 °C (77 K).

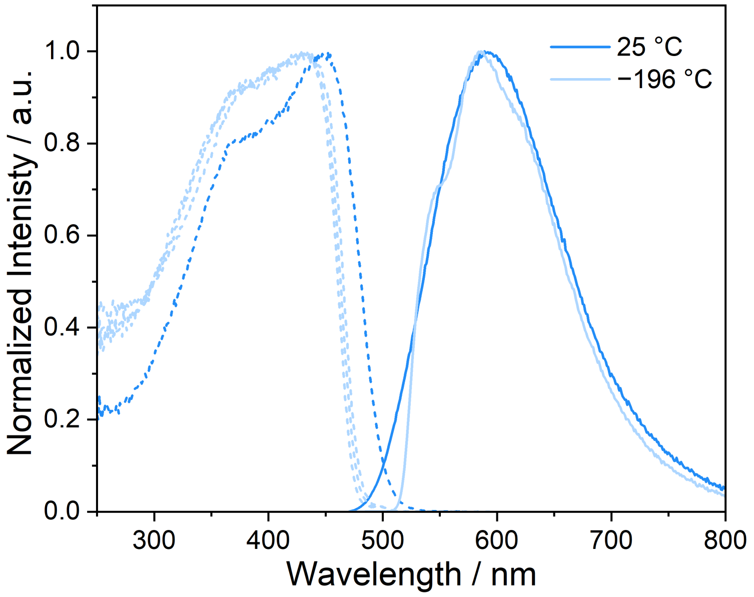

Figure S71. Excitation (left, dotted lines) and emission (right, solid lines) spectra of microcrystalline ***ds*CuRB** at 25 and –196 °C.

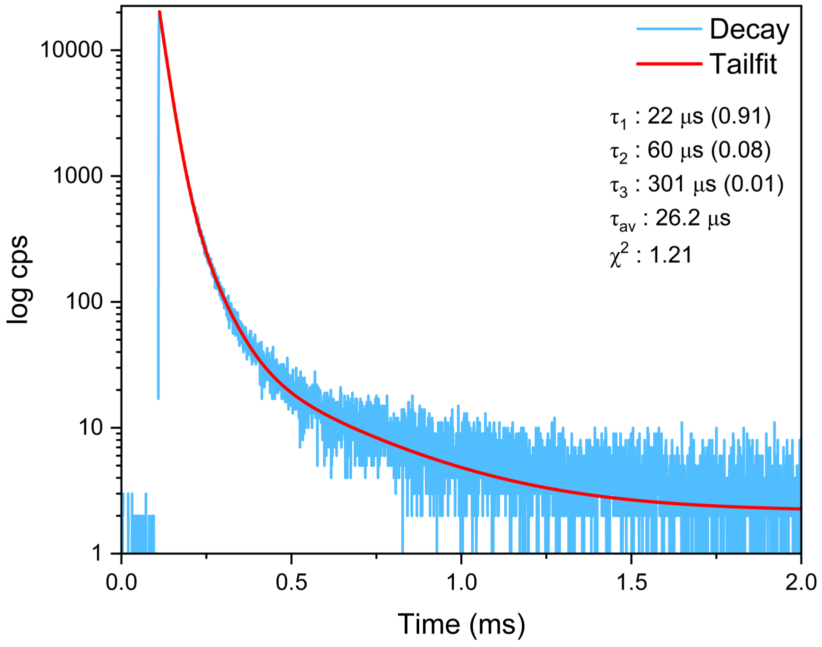

Figure S72. Emission lifetime decay of ***ds*CuRB** at 593 nm and 25 °C.

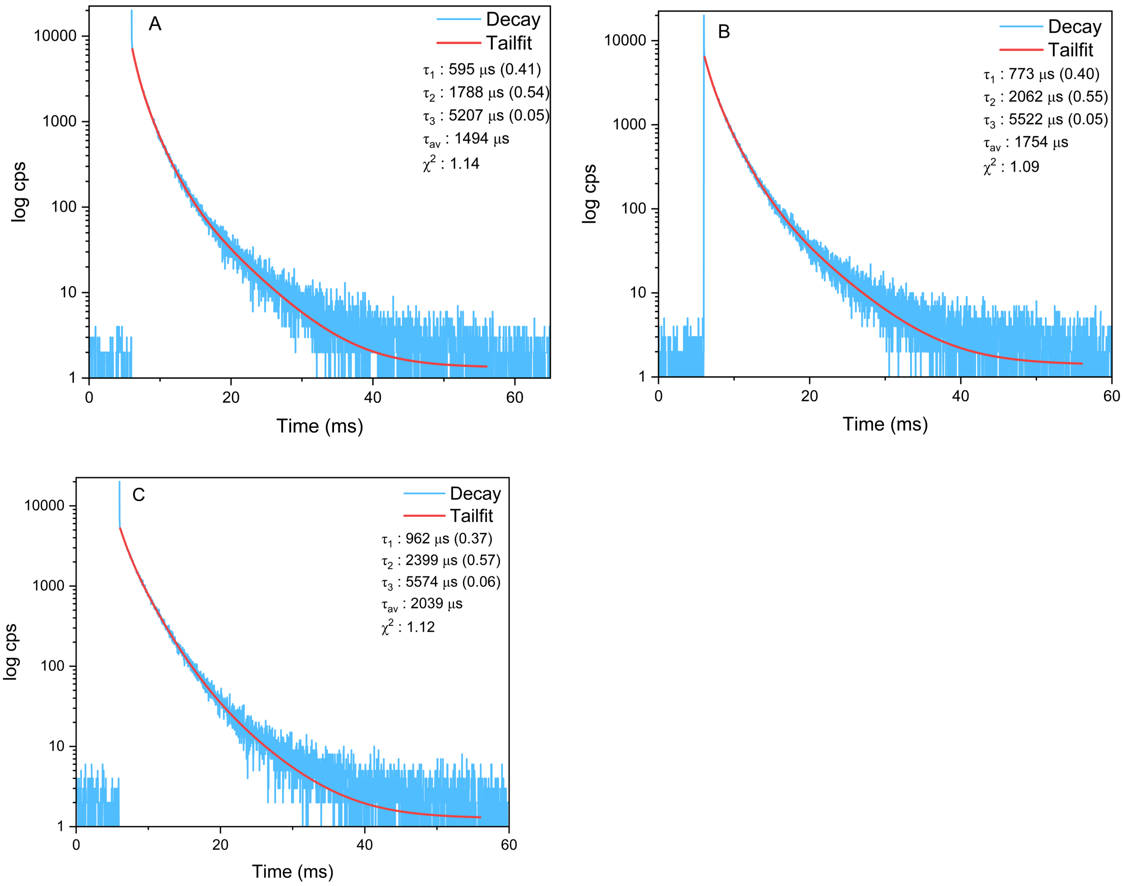

Figure S73. Emission lifetime decay of ***ds*CuRB** at (A) 547 nm (lifetime recorded at 520 nm), (B) 589 nm and (C) 623 nm (lifetime recorded at 690 nm) at –196 °C.

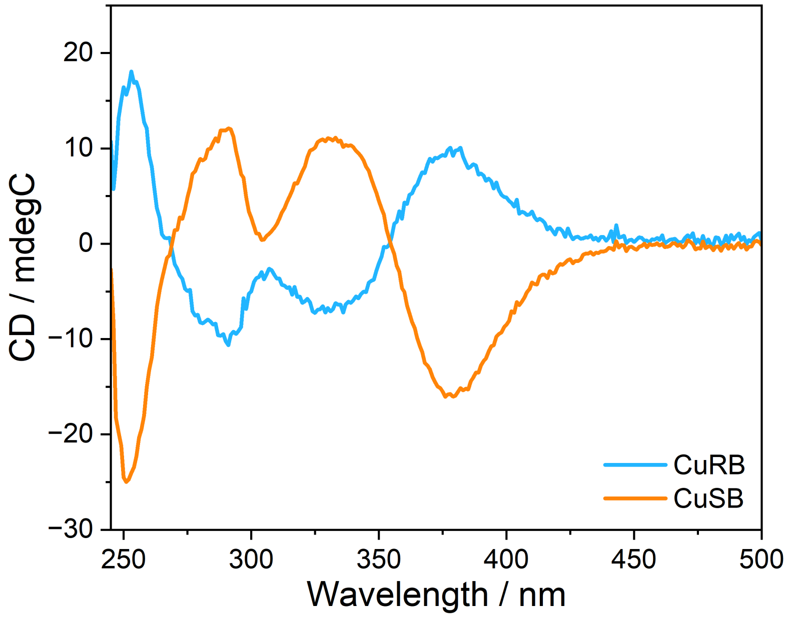

Figure S74. CD spectra of **CuRB** and **CuSB** in THF.

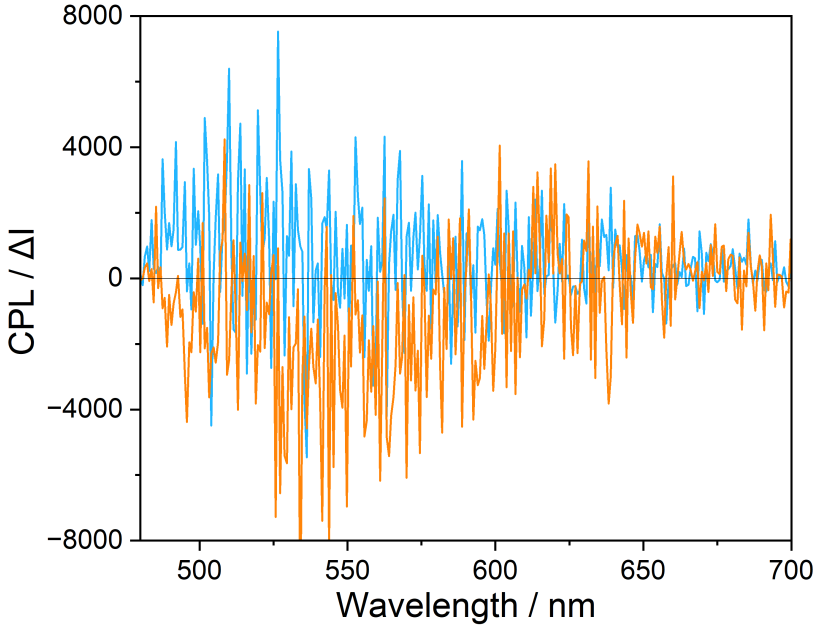

Figure S75. CPL spectra of microcrystalline **CuRB** and **CuSB**.

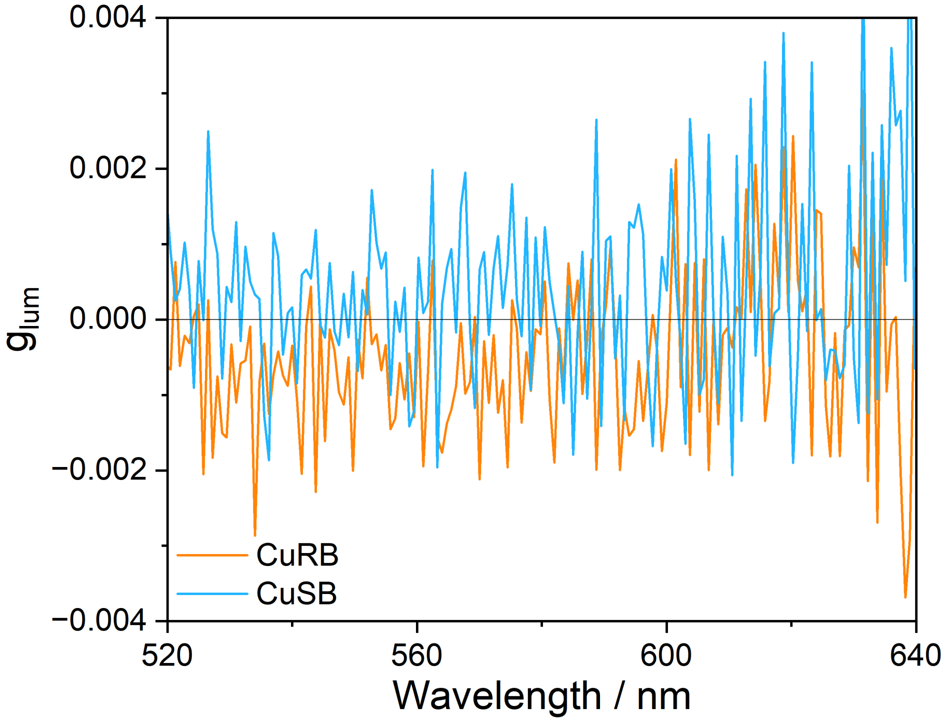

Figure S76. Dissymmetry factors g_{lum} of the photoluminescence of microcrystalline **CuRB** and **CuSB**.

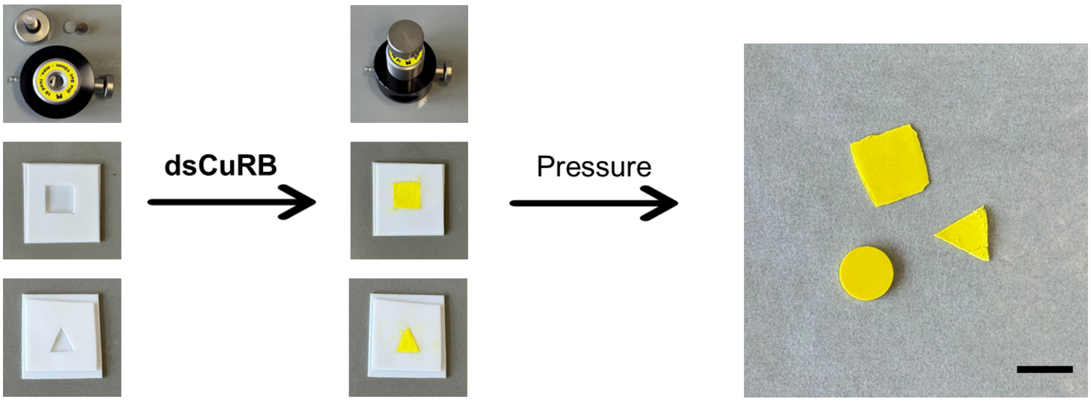

Figure S77. Procedure to obtain powder pellets of ***ds*CuRB**. A 10 mm pellet pressing die set or polytetrafluoroethylene modules with square or triangular shape were used.

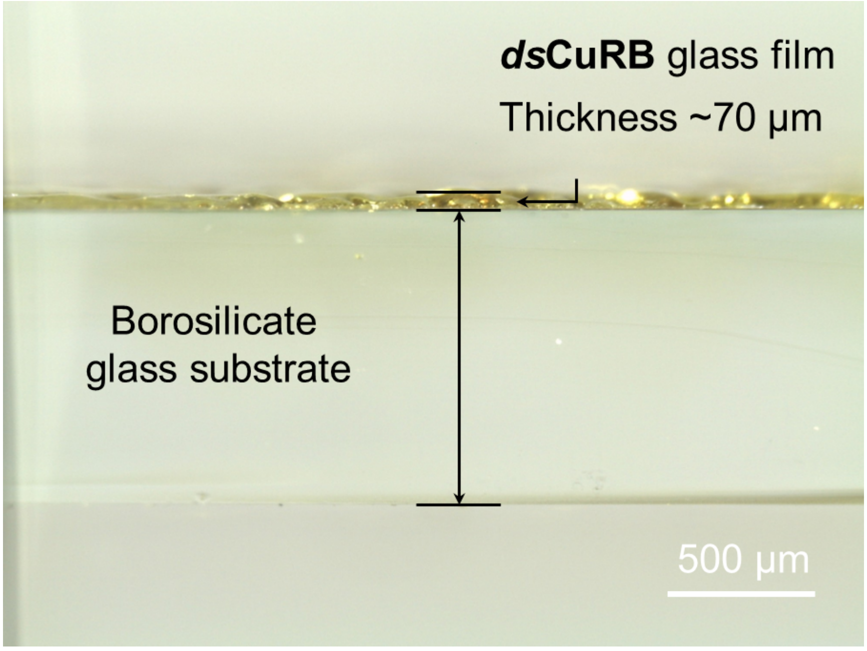

Figure S78. A cross-sectional optical microscopy image of the ***ds*CuRB** glass film. The thickness of the glass film is estimated to be ~70 µm.

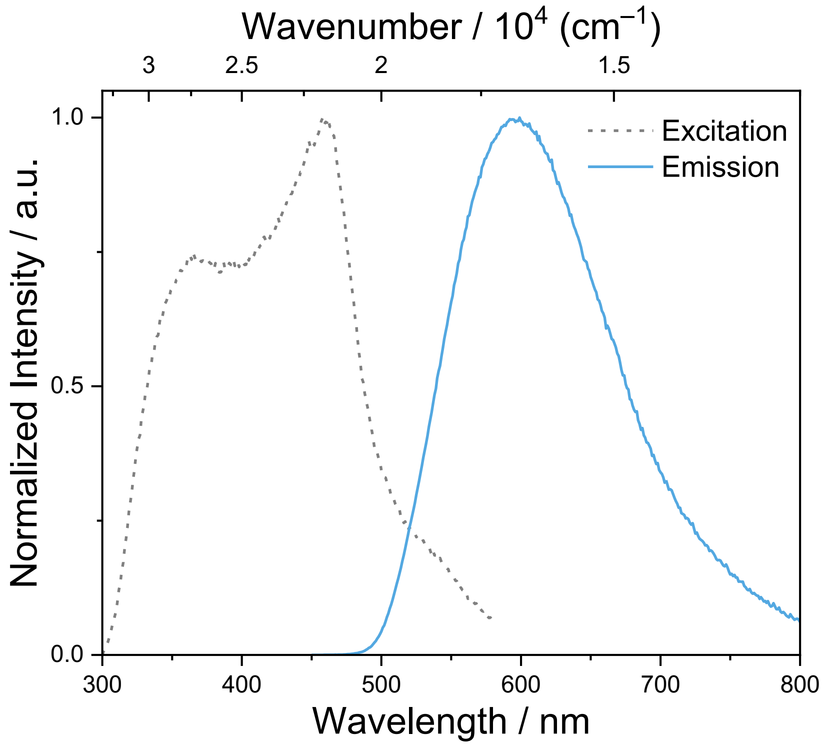

Figure S79. Excitation (left, dotted lines) and emission (right, solid lines) spectra of ***ds*****CuRB** film at 25 °C.

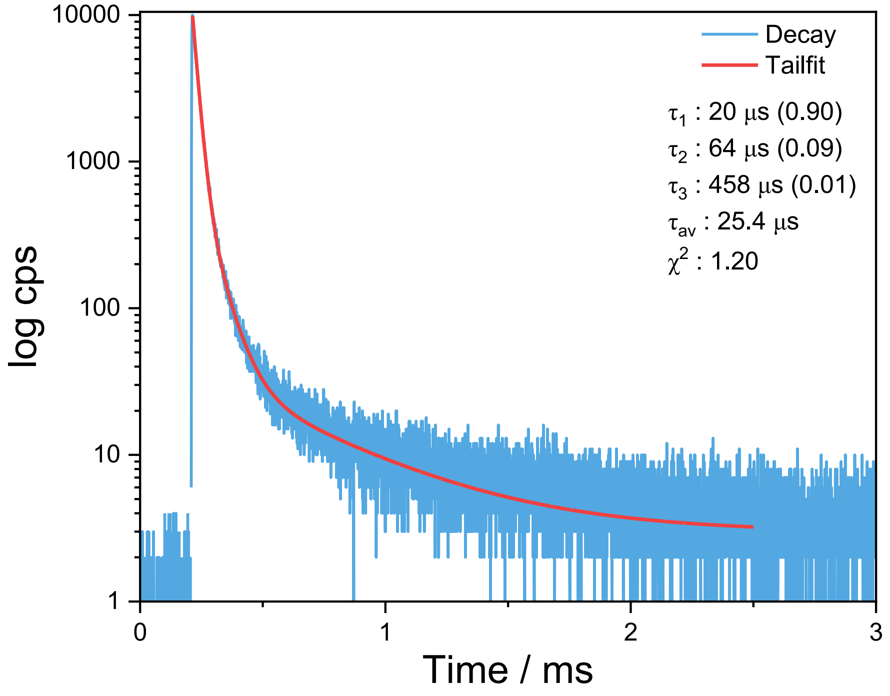

Figure S80. Emission lifetime decay of ***ds*****CuRB** film at 590 nm and 25 °C.

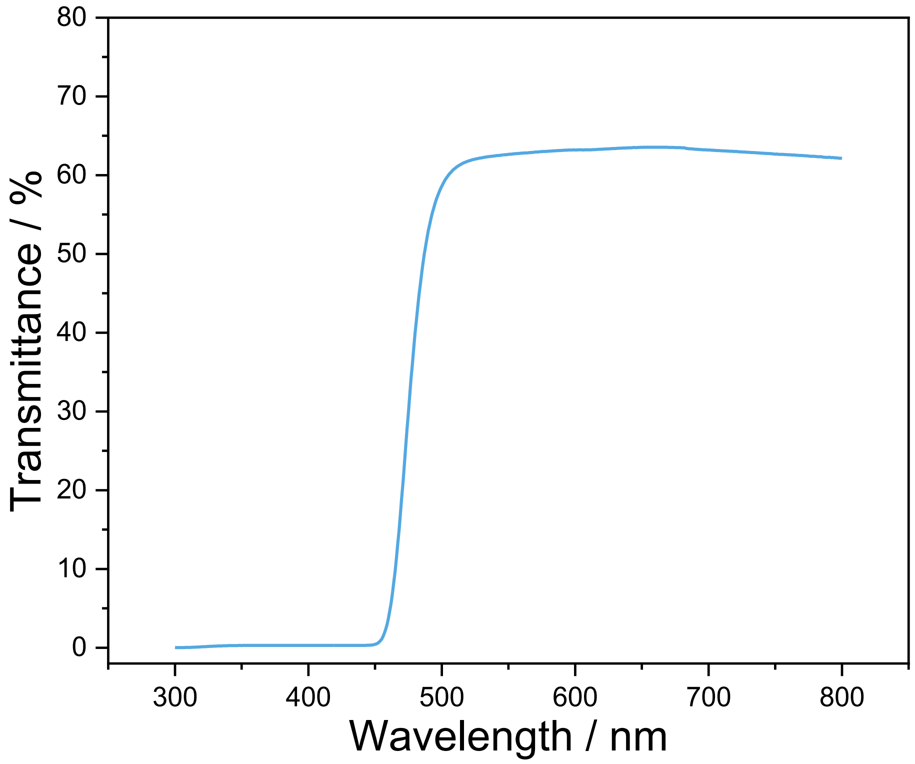

Figure S81. Transmittance of ***ds*CuRB** film with thickness of ~70 μm from 300 nm to 800 nm at 25 °C.

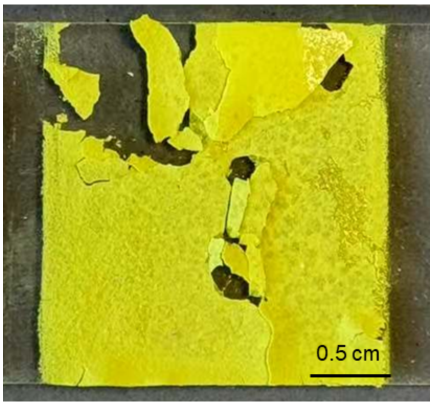

Figure S82. *ds*CuRB glass film on a borosilicate glass substrate after exposure to toluene/MeOH vapor for 2 days. A vial containing the neat glass film was sealed inside a 120 mL bottle with 20 mL solvent (toluene/methanol with a volume ratio of 1/4). The initially transparent glass film became opaque and exhibited significant peeling and fragmentation due to the formation of solvent-containing **CuRB** microcrystals.

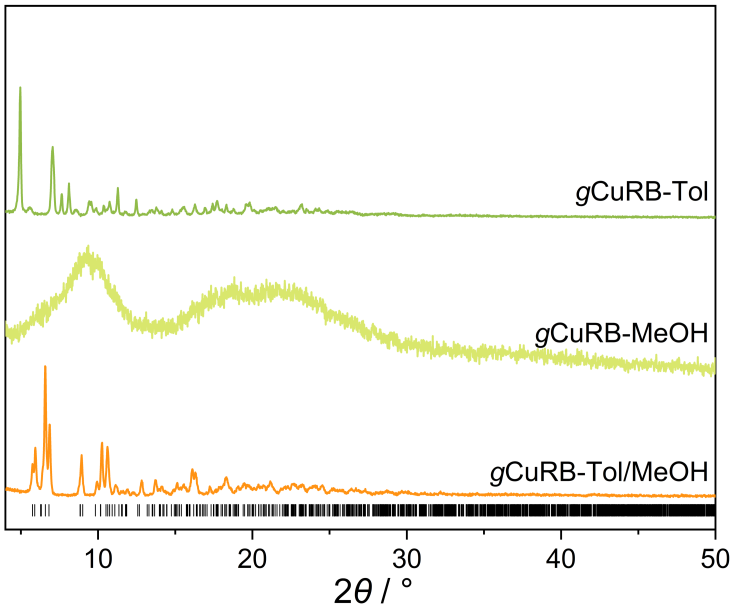

Figure S83. PXRD patterns of ***g*CuRB** treated with different vapor for 2d. Black tick marks indicate the positions of the allowed Bragg reflections calculated from the crystal structure of **CuRB**.

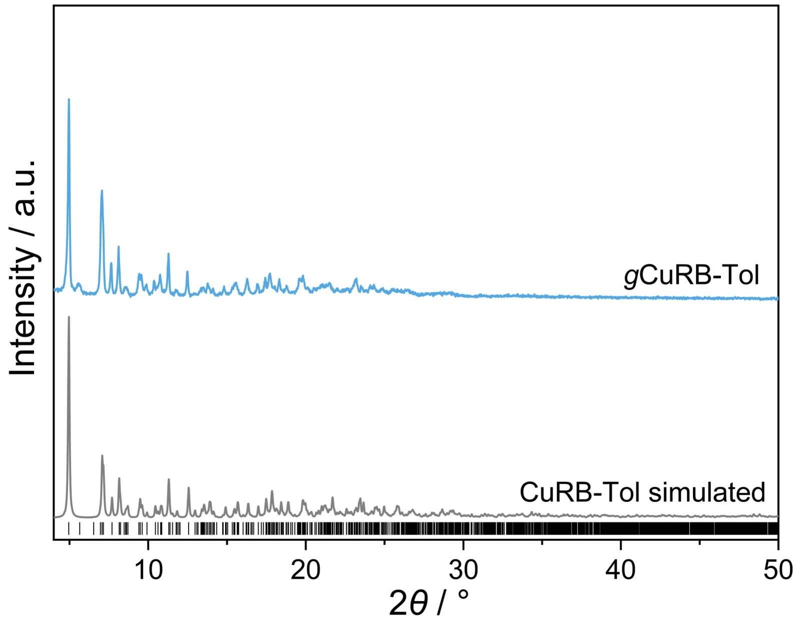

Figure S84. PXRD patterns of ***g*CuRB-Tol** and simulated **CuRB-Tol**. Black tick marks indicate the positions of the allowed Bragg reflections calculated from the crystal structure of **CuRB-Tol**.

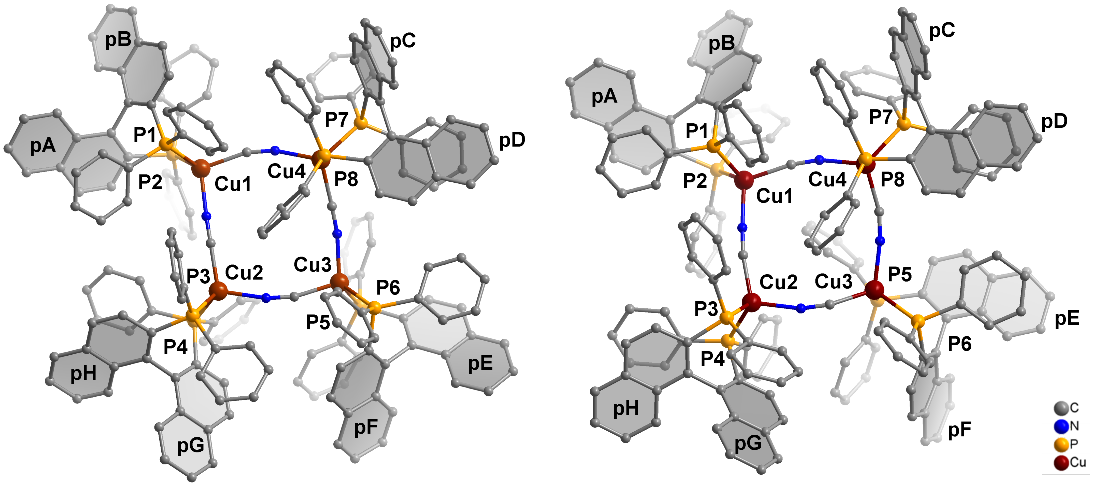

Figure S85. Crystal structures (ball and stick model) of **CuRB** (left) and **CuRB-Tol** (right). pA to pH represent the planes of the naphthalene rings.

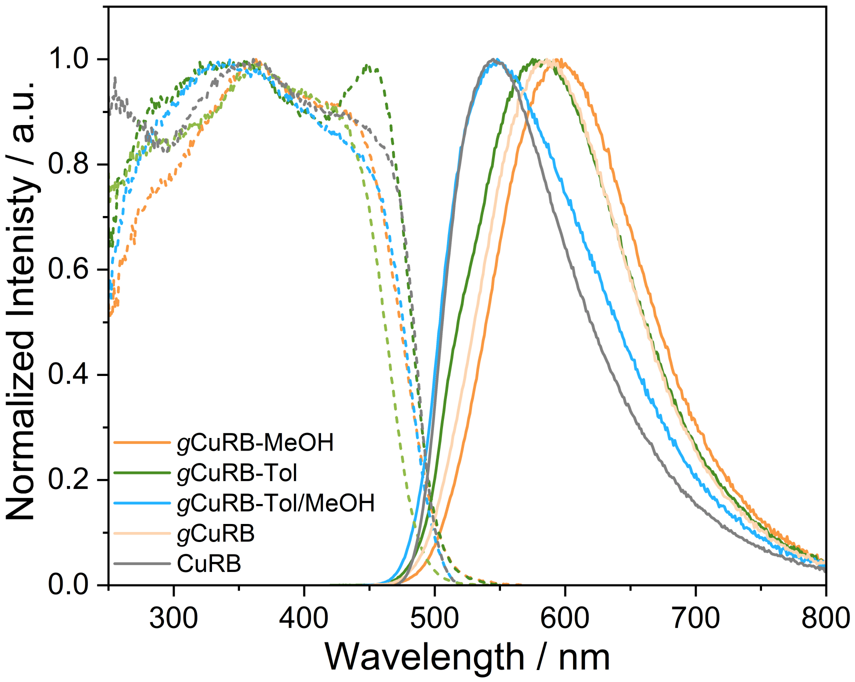

Figure S86. Excitation (left, dotted lines) and emission (right, solid lines) spectra of ***g*CuRB**, **CuRB** and ***g*CuRB** treated with methanol, toluene and toluene/methanol vapor.

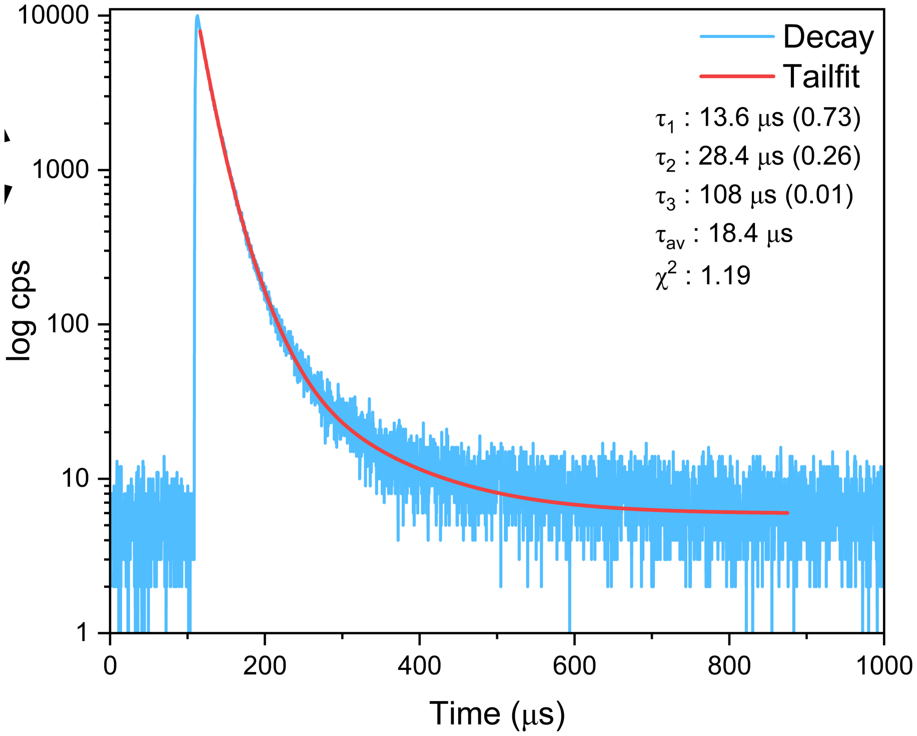

Figure S87. Emission lifetime decay of ***g*CuRB-Tol/MeOH** at 546 nm and 25 °C.

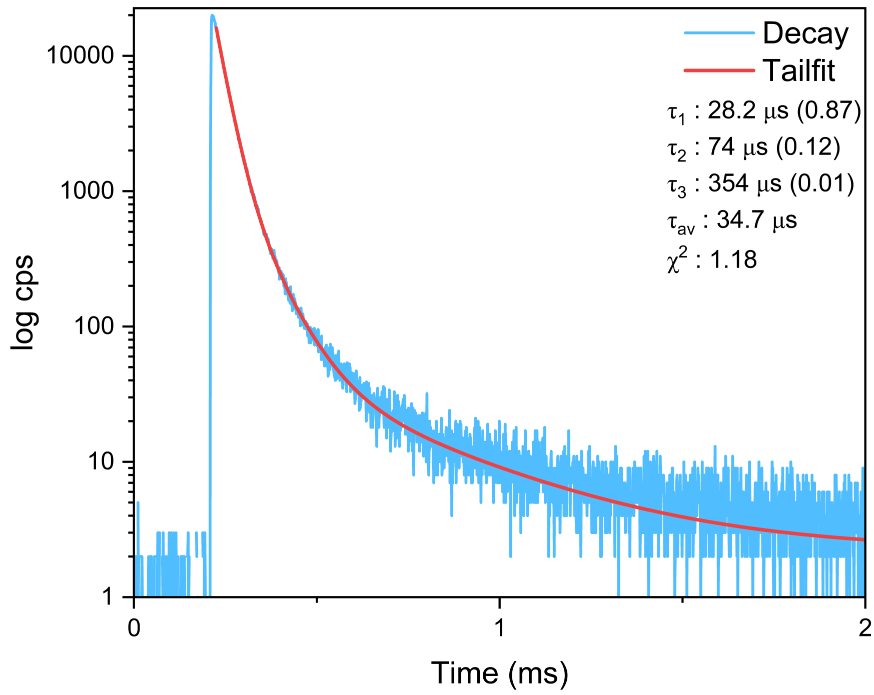

Figure S88. Emission lifetime decay of ***g*CuRB-Tol** at 570 nm and 25 °C.

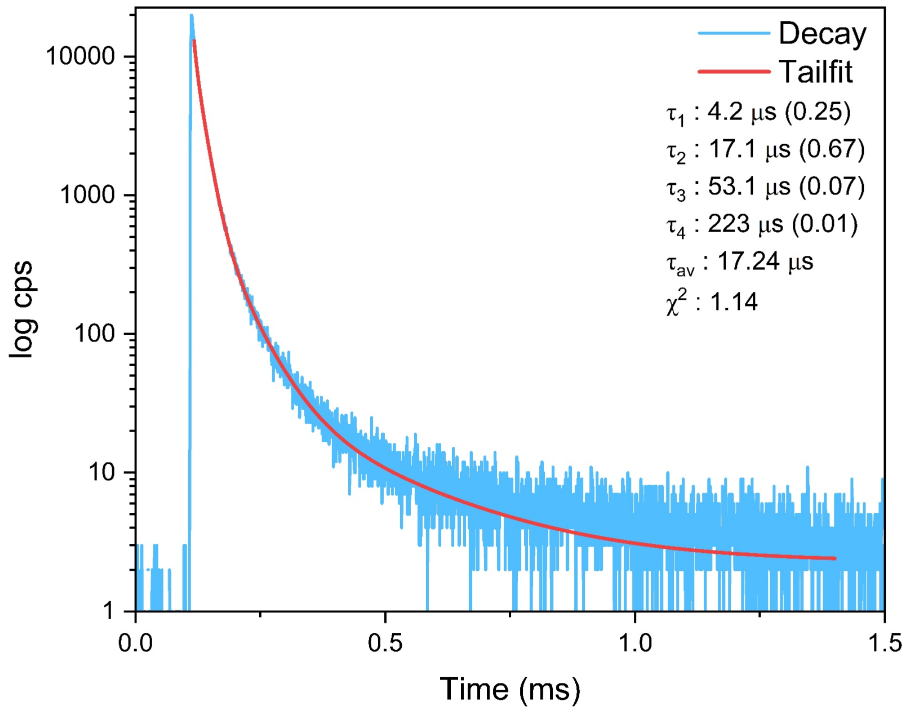

Figure S89. Emission lifetime decay of ***g*CuRB-MeOH** at 595 nm and 25 °C.

Table S1. Crystallographic data of **CuRB** from single crystal X-ray diffraction measurements.

Compound	**CuRB**
CCDC number	2486565
Empirical formula	$C_{194}H_{144}Cu_4N_4O_3P_8$
Formula weight	3081.04
Temperature/K	100
Crystal system	triclinic
Space group	$P1$
a / Å	15.8012(14)
b / Å	17.0403(14)
c / Å	17.7149(15)
α / °	108.238(3)
β / °	107.382(3)
γ / °	104.514(3)
Volume / Å^3	3999.7(6)
Z	1
ρ_{calc} g/cm^3	1.279
μ /mm^{-1}	0.662
F(000)	1596.0
Crystal size/mm^3	0.368 × 0.261 × 0.244
Radiation	Mo Kα (λ = 0.71073)
2θ range for data collection/°	4.072 to 58.582
Index ranges	-21 ≤ h ≤ 21, -23 ≤ k ≤ 23, -24 ≤ l ≤ 24
Reflections collected	42933
Independent reflections	42933 [R_{int} = 0.0962, R_{sigma} = 0.0556]
Data/restraints/parameters	42933/132/1948
Goodness-of-fit on F^2	1.082
Final R indexes [I>=2σ (I)]	R_1 = 0.0663, wR_2 = 0.1525
Final R indexes [all data]	R_1 = 0.0867, wR_2 = 0.1700
Largest diff. peak/hole / e Å^{-3}	0.88/-0.75
Flack parameter	0.064(11)

Table S2. Crystallographic data of **CuSB** from single crystal X-ray diffraction measurements.

Compound	**CuSB**
CCDC number	2486566
Empirical formula	$C_{194}H_{144}Cu_4N_4O_4P_8$
Formula weight	3097.04
Temperature/K	100
Crystal system	Triclinic
Space group	*P*1
a / Å	15.7993(6)
b / Å	17.0476(5)
c / Å	17.7149(6)
α / °	108.1910(10)
β / °	107.3870(10)
γ / °	104.5320(10)
Volume / Å^3	4001.9(2)
Z	1
ρ_{calc} g/cm^3	1.285
μ /mm^{-1}	0.663
F(000)	1604.0
Crystal size/mm^3	0.305 × 0.254 × 0.208
Radiation	MoKα (λ = 0.71073)
2θ range for data collection/°	4.152 to 61.18
Index ranges	-22 ≤ h ≤ 22, -24 ≤ k ≤ 24, -25 ≤ l ≤ 25
Reflections collected	274910
Independent reflections	48807 [R_{int} = 0.0513, R_{sigma} = 0.0457]
Data/restraints/parameters	48807/69/1966
Goodness-of-fit on F^2	1.028
Final R indexes [I>=2σ (I)]	R_1 = 0.0348, wR_2 = 0.0860
Final R indexes [all data]	R_1 = 0.0412, wR_2 = 0.0898
Largest diff. peak/hole / e Å^{-3}	0.61/-0.55
Flack parameter	0.023(4)

Table S3. Crystallographic data of ***meso*CuB** from single crystal X-ray diffraction measurements.

Compound	***meso*CuB**
CCDC number	2486564
Empirical formula	$C_{66}H_{56}CuNP_2$
Formula weight	988.59
Temperature/K	100.00
Crystal system	tetragonal
Space group	$I4_1/a$
a / Å	39.8056(11)
b / Å	39.8056(11)
c / Å	13.4078(6)
α / °	90
β / °	90
γ / °	90
Volume / Å^3	21244.5(15)
Z	16
ρ_{calc} g/cm^3	1.236
μ /mm^{-1}	1.459
F(000)	8288.0
Crystal size/mm^3	0.14 × 0.12 × 0.1
Radiation	CuKα (λ = 1.54178)
2θ range for data collection/°	6.28 to 137.754
Index ranges	-48 ≤ h ≤ 48, -48 ≤ k ≤ 47, -13 ≤ l ≤ 16
Reflections collected	172482
Independent reflections	9817 [R_{int} = 0.0888, R_{sigma} = 0.0287]
Data/restraints/parameters	9817/0/506
Goodness-of-fit on F^2	1.067
Final R indexes [I>=2σ (I)]	R_1 = 0.0842, wR_2 = 0.1995
Final R indexes [all data]	R_1 = 0.0961, wR_2 = 0.2110
Largest diff. peak/hole / e Å^{-3}	1.03/-0.49

Table S4. Crystallographic data of **CuRB-Tol** from single crystal X-ray diffraction measurements.

Compound	**CuRB-Tol**
CCDC number	2543814
Empirical formula	$C_{230}H_{188}Cu_4N_4OP_8$
Formula weight	3525.75
Temperature/K	100.00
Crystal system	monoclinic
Space group	$P2_1$
a / Å	13.5209(8)
b / Å	21.6712(15)
c / Å	31.291(2)
α / °	90
β / °	90.961(2)
γ / °	90
Volume / $Å^3$	9167.4(10)
Z	2
ρ_{calc} g/cm^3	1.277
μ /mm^{-1}	0.586
F(000)	3680.0
Crystal size/mm^3	0.12 × 0.1 × 0.1
Radiation	MoKα (λ = 0.71073)
2*θ* range for data collection/°	1.302 to 56.566
Index ranges	-18 ≤ h ≤ 18, -28 ≤ k ≤ 287, -41 ≤ l ≤ 41
Reflections collected	485729
Independent reflections	45406 [R_{int} = 0.0718, R_{sigma} = 0.0374]
Data/restraints/parameters	45406/4513/2345
Goodness-of-fit on F^2	1.101
Final R indexes [I>=2σ (I)]	R_1 = 0.0690, wR_2 = 0.1754
Final R indexes [all data]	R_1 = 0.0731, wR_2 = 0.1788
Largest diff. peak/hole / e $Å^{-3}$	2.95/-0.487
Flack parameter	0.0550(18)

Table S5. Unit cell parameters and corresponding R_{wp}, R_{exp} and χ values determined by the Pawley refinement of as-synthesized microcrystalline **CuRB** and **CuSB**.

Compound	**CuRB**	**CuSB**
Crystal system	Triclinc	Triclinc
Space group	*P*1	*P*1
a / Å	15.628(7)	15.624(4)
b / Å	17.238(9)	17.218(4)
c / Å	18.151(9)	18.180(4)
α / °	110.5(3)	110.61(1)
β / °	107.5(2)	107.56(1)
γ / °	103.6(2)	103.65(1)
Volume / $Å^3$	4032(4)	4030(2)
R_{wp}	5.59	5.40
R_{exp}	3.39	3.23
χ	1.65	1.67

Table S6. Elemental analysis data of the metal complexes in this study.

Compound	Composition	Calculated			Experimental		
		%C	%H	%N	%C	%H	%N
**CuRB**	$Cu_4(CN)_4$(*R*-BINAP)$_4$·2toluene·3H_2O	75.5	4.9	1.8	75.5	4.9	1.9
***ds*CuRB**	$Cu_4(CN)_4$(*R*-BINAP)$_4$	75.9	4.5	2.0	75.6	4.5	2.1
***g*CuRB**	$Cu_4(CN)_4$(*R*-BINAP)$_4$	75.9	4.5	2.0	75.4	4.5	2.0
**CuSB**	$Cu_4(CN)_4$(*S*-BINAP)$_4$·2toluene·4H_2O	75.0	4.9	1.8	76.8	4.8	2.0
***ds*CuSB**	$Cu_4(CN)_4$(*S*-BINAP)$_4$	75.9	4.5	2.0	75.7	4.6	2.0
***g*CuSB**	$Cu_4(CN)_4$(*S*-BINAP)$_4$	75.9	4.5	2.0	75.7	4.7	2.0

Table S7. Summary of the parameters of the cyanide vibration in the crystalline and glassy metal complexes in this study.

Compound	Position of ν_{CN} / cm^{-1}	FWHM / cm^{-1}
**CuRB**	2120	22.0
***ds*CuRB**	2119	27.5
***g*CuRB**	2119	30.5
**CuSB**	2120	20.5
***ds*CuSB**	2120	29.0
***g*CuSB**	2119	31.0

Table S8. EXAFS fitting parameters of **CuRB**. R^2=0.005

Path	*Path degeneracy*	S_0^2	ΔE_0 / eV	R / Å	σ^2
Cu-C	1.0	1.0	11.8	1.95±0.02	0.003
Cu-N	1.0	1.0	11.8	2.08±0.02	0.002
Cu-P	2.0	1.0	11.8	2.31±0.03	0.009
Cu-C	1.0	1.0	11.8	3.13±0.06	0.005
Cu-N	1.0	1.0	11.8	3.14±0.05	0.006
Cu-C	2.0	1.0	11.8	3.33±0.05	0.004

Table S9. EXAFS fitting parameters of **CuSB**. R^2=0.006

Path	*Path degeneracy*	S_0^2	ΔE_0 / eV	R / Å	σ^2
Cu-C	1.0	1.0	8.8	1.94±0.02	0.004
Cu-N	1.0	1.0	8.8	2.08±0.01	0.008
Cu-P	2.0	1.0	8.8	2.27±0.01	0.009
Cu-C	1.0	1.0	8.8	3.15±0.08	0.003
Cu-N	1.0	1.0	8.8	3.05±0.04	0.005
Cu-C	2.0	1.0	8.8	3.38±0.05	0.005

Table S10. EXAFS fitting parameters of ***ds*CuRB**. R^2=0.010

Path	*Path degeneracy*	S_0^2	ΔE_0 / eV	R / Å	σ^2
Cu-C	1.0	1.0	2.4	1.90±0.02	0.004
Cu-N	1.0	1.0	2.4	2.07±0.05	0.008
Cu-P	2.0	1.0	2.4	2.25±0.02	0.009
Cu-C	1.0	1.0	2.4	3.15±0.04	0.008
Cu-N	1.0	1.0	2.4	3.02±0.02	0.005
Cu-C	2.0	1.0	2.4	3.34±0.03	0.002

Table S11. EXAFS fitting parameters of ***ds*CuSB**. R^2=0.011

Path	*Path degeneracy*	S_0^2	ΔE_0 / eV	R / Å	σ^2
Cu-C	1.0	1.0	3.3	1.89±0.01	0.004
Cu-N	1.0	1.0	3.3	2.01±0.01	0.009
Cu-P	2.0	1.0	3.3	2.25±0.01	0.009
Cu-C	1.0	1.0	3.3	3.20±0.02	0.003
Cu-N	1.0	1.0	3.3	3.06±0.01	0.005
Cu-C	2.0	1.0	3.3	3.32±0.03	0.003

Table S12. EXAFS fitting parameters of ***g*CuRB**. R^2=0.011

Path	*Path degeneracy*	S_0^2	ΔE_0 / eV	R / Å	σ^2
Cu-C	1.0	1.0	0.6	1.91±0.01	0.003
Cu-N	1.0	1.0	0.6	2.03±0.02	0.009
Cu-P	2.0	1.0	0.6	2.24±0.01	0.008
Cu-C	1.0	1.0	0.6	3.26±0.01	0.006
Cu-N	1.0	1.0	0.6	3.05±0.01	0.005
Cu-C	2.0	1.0	0.6	3.36±0.03	0.003

Table S13. EXAFS fitting parameters of ***g*CuSB**. R^2=0.006

Path	*Path degeneracy*	S_0^2	ΔE_0 / eV	R / Å	σ^2
Cu-C	1.0	1.0	10.6	1.94±0.01	0.001
Cu-N	1.0	1.0	10.6	2.11±0.01	0.001
Cu-P	2.0	1.0	10.6	2.28±0.01	0.009
Cu-C	1.0	1.0	10.6	3.13±0.03	0.003
Cu-N	1.0	1.0	10.6	3.10±0.03	0.003
Cu-C	2.0	1.0	10.6	3.32±0.04	0.008

Table S14. TD-DFT calculated vertical electronic transitions from the DFT optimized ground state S_0 of **CuRB** to the first 40 singlet and 15 triplet excited states.

Transition			Energy (eV)	Energy (cm^{-1})	Wavelength (nm)	Oscillator strength f
0-1A	→	1-1A	2.948	23775.2	420.6	0.0467
0-1A	→	2-1A	3.021	24366.2	410.4	0.0767
0-1A	→	3-1A	3.090	24920.2	401.3	0.0472
0-1A	→	4-1A	3.104	25033.9	399.5	0.0783
0-1A	→	5-1A	3.137	25299.5	395.3	0.0473
0-1A	→	6-1A	3.247	26192.8	381.8	0.0458
0-1A	→	7-1A	3.269	26365.3	379.3	0.0240
0-1A	→	8-1A	3.282	26474.6	377.7	0.0023
0-1A	→	9-1A	3.296	26587.5	376.1	0.0042
0-1A	→	10-1A	3.326	26822.9	372.8	0.0461
0-1A	→	11-1A	3.329	26853.0	372.4	0.0189
0-1A	→	12-1A	3.336	26906.8	371.7	0.0097
0-1A	→	13-1A	3.350	27022.4	370.1	0.0098
0-1A	→	14-1A	3.406	27473.7	364.0	0.0120
0-1A	→	15-1A	3.444	27773.7	360.1	0.0024
0-1A	→	16-1A	3.445	27789.4	359.8	0.0080
0-1A	→	17-1A	3.467	27964.6	357.6	0.0105
0-1A	→	18-1A	3.478	28048.0	356.5	0.0021
0-1A	→	19-1A	3.489	28137.7	355.4	0.0095
0-1A	→	20-1A	3.498	28210.9	354.5	0.0072
0-1A	→	21-1A	3.510	28313.5	353.2	0.0010
0-1A	→	22-1A	3.520	28391.6	352.2	0.0037
0-1A	→	23-1A	3.530	28470.5	351.2	0.0123
0-1A	→	24-1A	3.539	28544.6	350.3	0.0250
0-1A	→	25-1A	3.548	28618.0	349.4	0.0136
0-1A	→	26-1A	3.553	28655.1	349.0	0.0221
0-1A	→	27-1A	3.604	29066.7	344.0	0.0045
0-1A	→	28-1A	3.618	29181.1	342.7	0.0019
0-1A	→	29-1A	3.635	29314.5	341.1	0.0041
0-1A	→	30-1A	3.638	29345.3	340.8	0.0060
0-1A	→	31-1A	3.642	29376.4	340.4	0.0125
0-1A	→	32-1A	3.646	29403.9	340.1	0.0102
0-1A	→	33-1A	3.647	29414.9	340.0	0.0035
0-1A	→	34-1A	3.668	29583.6	338.0	0.0010
0-1A	→	35-1A	3.682	29696.6	336.7	0.0015

0-1A	→	36-1A	3.685	29718.3	336.5	0.0094
0-1A	→	37-1A	3.696	29810.3	335.5	0.0124
0-1A	→	38-1A	3.702	29854.8	335.0	0.0137
0-1A	→	39-1A	3.718	29985.6	333.5	0.0068
0-1A	→	40-1A	3.723	30032.0	333.0	0.0035
0-1A	→	1-3A	2.619	21120.6	473.5	
0-1A	→	2-3A	2.668	21522.7	464.6	
0-1A	→	3-3A	2.684	21645.2	462.0	
0-1A	→	4-3A	2.715	21895.6	456.7	
0-1A	→	5-3A	2.721	21949.3	455.6	
0-1A	→	6-3A	2.723	21960.9	455.4	
0-1A	→	7-3A	2.737	22072.6	453.1	
0-1A	→	8-3A	2.745	22136.6	451.7	
0-1A	→	9-3A	2.932	23644.2	422.9	
0-1A	→	10-3A	2.989	24110.9	414.7	
0-1A	→	11-3A	3.037	24495.2	408.2	
0-1A	→	12-3A	3.048	24582.3	406.8	
0-1A	→	13-3A	3.136	25296.4	395.3	
0-1A	→	14-3A	3.213	25914.3	385.9	
0-1A	→	15-3A	3.237	26110.5	383.0	

Table S15. Variable temperature lifetime measurements of ***g*****CuRB**.

Temp (K)	λ_{em} / nm	/ τ μs	τ_1 / μs	τ_2 / μs	τ_3 / μs	τ_4 / μs
77	548	1895	665.4 (0.41)	2227 (0.51)	6283.6 (0.08)	
	578	2119	929.6 (0.46)	2666.5 (0.49)	7320 (0.05)	
	630	2412	1078.3 (0.37)	2797 (0.57)	7393.5 (0.05)	
87	550	1886	771.6 (0.47)	2373 (0.48)	6456.8 (0.05)	
	582	1976	696 (0.38)	2288 (0.55)	6349 (0.07)	
	630	2436	1217 (0.41)	2922.7 (0.54)	7508.2 (0.05)	
97	555	1690	510 (0.41)	2048 (0.52)	5916 (0.07)	
	586	1960	722.5 (0.39)	2295.7 (0.54)	6122.1 (0.07)	
	635	2401	948 (0.28)	2604 (0.65)	6470 (0.07)	
107	555	1486	378.1 (0.41)	1798 (0.51)	5147.4 (0.08)	
	591	1855	623 (0.38)	2200.4 (0.55)	5850 (0.07)	
	640	2310	1098 (0.36)	2644 (0.58)	6581 (0.06)	
117	559	1375	366 (0.42)	1662 (0.49)	4602 (0.09)	
	595	1820	691 (0.42)	2308.7 (0.54)	6466.5 (0.04)	
	648	2231	941 (0.32)	2546.3 (0.63)	6428.4 (0.05)	
127	562	1190	264 (0.43)	1431.5 (0.48)	4046.7 (0.09)	
	600	1700	578.5 (0.38)	2055 (0.55)	5341 (0.07)	
	651	2051	876.5 (0.37)	2495 (0.60)	6545 (0.03)	
137	560	866	137.4 (0.45)	1006.7 (0.42)	3115.2 (0.07)	
	600	1451	431.3 (0.40)	1805 (0.53)	4849 (0.07)	
	651	1741	485 (0.30)	1975 (0.62)	4867 (0.08)	
147	565	613	94 (0.52)	745 (0.37)	2547 (0.11)	

	605	1254	399 (0.46)	1705 (0.49)	4823 (0.05)	
	660	1422	364 (0.37)	1741.3 (0.56)	4371.3 (0.07)	
	565	320	68.4 (0.62)	479.5 (0.30)	1767.8 (0.08)	
167	610	430	40.5 (0.47)	307.9 (0.30)	1145.3 (0.20)	3172.9 (0.03)
	670	498	40.8 (0.45)	331.3 (0.30)	1299.4 (0.22)	3466 (0.03)
	565	223	31 (0.45)	161 (0.35)	601.1 (0.17)	1715.3 (0.03)
187	615	207	26 (0.50)	157 (0.32)	623 (0.15)	1841 (0.03)
	670	204	30 (0.55)	177 (0.30)	730.4 (0.13)	2026 (0.02)
207	605	116	21.4 (0.50)	100 (0.35)	356.6 (0.13)	1072.4 (0.02)
227	600	76.5	29.6 (0.67)	135 (0.29)	510.4 (0.04)	
247	598	48	23 (0.69)	87 (0.29)	325 (0.02)	
267	595	32	18 (0.70)	52 (0.29)	432 (0.01)	
287	591	26	18 (0.81)	49 (0.18)	415 (0.01)	

Table S16. Photophysical parameters of the samples measured in this study

Sample	*T* / °C	λ_{em} / nm	τ / μs	τ_{av} / μs	Φ	k_r (×10^3) / s^{-1}	k_{nr} (×10^3) / s^{-1}
**CuRB** (THF)	25	545	175 (0.52), 287 (0.48)	229	0.07	0.31	4.06
**CuRB** (Toluene)	25	545	143 (0.80), 232 (0.20)	161	0.45	2.8	3.41
**CuRB** (microcrystalline)	25	545	36 (0.60), 78 (0.39), 446 (0.01)	53.5	0.05	0.93	17.8
	–196	545	678 (0.37), 1594 (0.58), 3106 (0.05)	1327			
		577	1078 (0.50), 2042 (0.49), 5517 (0.01)	1576	0.61	0.39	0.25
		645	1386 (0.70), 2421 (0.29) 78289 (0.01)	1789			
***ds*CuRB**	25	593	22 (0.91), 60 (0.08), 301 (0.01)	26.2	0.11	4.2	33.9
	–196	547	595 (0.41), 1788 (0.54), 5207 (0.05)	1494			
		589	773 (0.40), 2062 (0.55), 5522 (0.05)	1754	0.42	0.24	0.33
		623	962 (0.37), 2399 (0.57), 5574 (0.06)	2039			
***g*CuRB**	25	590	21 (0.90), 59 (0.09), 313 (0.01)	25.5	0.07	2.8	37.8
	–196	548	665.4 (0.41), 2227 (0.51), 6283 (0.08)	1895			
		578	929.6 (0.46), 2666.5 (0.49), 7320 (0.05)	2119	0.42	0.18	0.25
		630	1078.3 (0.37), 2797 (0.57), 7393 (0.05)	2412			
***g*CuSB**	25	591	21 (0.91), 59 (0.08), 342 (0.01)	24.8	0.08	3.23	37.2
	–196	547	1084 (0.57), 3161 (0.40), 9491 (0.03)	2187			
		580	1198 (0.57), 3331 (0.40), 10042 (0.03)	2305	0.43	0.19	0.25
		630	1410 (0.52), 3483 (0.46), 10456 (0.02)	2607			
***g*CuRB-Tol/MeOH**	25	546	13.6 (0.73), 28.4 (0.26), 108 (0.01)	18.4			
***g*CuRB-MeOH**	25	595	4.2 (0.25), 17.1 (0.67), 53.1 (0.07), 223 (0.01)	17.2			
***g*CuRB-Tol**	25	570	28.2 (0.87), 74 (0.12), 354 (0.01)	34.7			

Table S17. Comparison of selected bond angles and dihedral angles between **CuRB** and **CuRB-Tol**.

Compound	CuRB	CuRB-Tol
∠P1-Cu1-P2 / °	96.66(7)	98.93(7)
∠P3-Cu2-P4 / °	99.90(7)	100.27(8)
∠P5-Cu3-P6 / °	97.47(7)	99.79(8)
∠P7-Cu4-P8 / °	98.36(8)	88.83(7)
∠pA-pB / °	70.3(3)	75.81(13)
∠pC-pD / °	80.2(3)	75.16(19)
∠pE-pF / °	71.7 (2)	71.7(3)
∠pG-pH / °	73.9(2)	77.8(3)

References

[1] P. Juhás, T. Davis, C. L. Farrow, S. J. L. Billinge, *J. Appl. Crystallogr.* **2013**, *46*, 560-566.

[2] D. A. Keen, *J. Appl. Crystallogr.* **2001**, *34*, 172-177.

[3] A. K. Soper, E. R. Barney, *J. Appl. Crystallogr.* **2011**, *44*, 714-726.

[4] B. Ravel, M. Newville, *J. Synchrotron Rad.* **2005**, *12*, 537-541.

[5] F. Neese, *WIREs Comput. Mol. Sci.* **2022**, *12*.

[6] A. D. Becke, *Phys. Rev. A Gen. Phys.* **1988**, *38*, 3098-3100.

[7] F. Weigend, R. Ahlrichs, *Phys. Chem. Chem. Phys.* **2005**, *7*, 3297-3305.

[8] F. Weigend, *Phys. Chem. Chem. Phys.* **2006**, *8*, 1057-1065.

[9] D. A. Pantazis, X. Y. Chen, C. R. Landis, F. Neese, *J. Chem. Theory Comput.* **2008**, *4*, 908-919.

[10] D. A. Pantazis, F. Neese, *J. Chem. Theory Comput.* **2009**, *5*, 2229-2238.

[11] D. A. Pantazis, F. Neese, *Theor. Chem. Acc.* **2012**, *131*.

[12] D. A. Pantazis, F. Neese, *J. Chem. Theory Comput.* **2011**, *7*, 677-684.

[13] J. D. Rolfes, F. Neese, D. A. Pantazis, *J. Comput. Chem.* **2020**, *41*, 1842-1849.

[14] S. Grimme, J. Antony, S. Ehrlich, H. Krieg, *J. Chem. Phys.* **2010**, *132*, 154104.

[15] S. Grimme, S. Ehrlich, L. Goerigk, *J. Comput. Chem.* **2011**, *32*, 1456-1465.

[16] J. P. Perdew, K. Burke, M. Ernzerhof, *Phys. Rev. Lett.* **1996**, *77*, 3865-.

[17] C. Adamo, V. Barone, *J. Chem. Phys.* **1999**, *110*, 6158-6170. 3868

[18] E. F. Pettersen, T. D. Goddard, C. C. Huang, G. S. Couch, D. M. Greenblatt, E. C. Meng, T. E. Ferrin, *J. Comput. Chem.* **2004**, *25*, 1605-1612.

[19] J. B. Weiss, JBWeiss-chem/xPDFsim: 0.0.3, https://doi.org/10.5281/zenodo.17313574
